# B‒N covalent bond-involved π-extension of multiple resonance emitters enables high-performance narrowband electroluminescence

**DOI:** 10.1093/nsr/nwae115

**Published:** 2024-03-23

**Authors:** Xingyu Huang, Jiahui Liu, Yulin Xu, Guohao Chen, Manli Huang, Mingxin Yu, Xialei Lv, Xiaojun Yin, Yang Zou, Jingsheng Miao, Xiaosong Cao, Chuluo Yang

**Affiliations:** Shenzhen Key Laboratory of New Information Display and Storage Materials, College of Materials Science and Engineering, Shenzhen University, Shenzhen 518060, China; Shenzhen Key Laboratory of New Information Display and Storage Materials, College of Materials Science and Engineering, Shenzhen University, Shenzhen 518060, China; College of Physics and Optoelectronic Engineering, Shenzhen University, Shenzhen 518060, China; Shenzhen Key Laboratory of New Information Display and Storage Materials, College of Materials Science and Engineering, Shenzhen University, Shenzhen 518060, China; Shenzhen Key Laboratory of New Information Display and Storage Materials, College of Materials Science and Engineering, Shenzhen University, Shenzhen 518060, China; Shenzhen Key Laboratory of New Information Display and Storage Materials, College of Materials Science and Engineering, Shenzhen University, Shenzhen 518060, China; Shenzhen Key Laboratory of New Information Display and Storage Materials, College of Materials Science and Engineering, Shenzhen University, Shenzhen 518060, China; Shenzhen Key Laboratory of New Information Display and Storage Materials, College of Materials Science and Engineering, Shenzhen University, Shenzhen 518060, China; Shenzhen Key Laboratory of New Information Display and Storage Materials, College of Materials Science and Engineering, Shenzhen University, Shenzhen 518060, China; Shenzhen Key Laboratory of New Information Display and Storage Materials, College of Materials Science and Engineering, Shenzhen University, Shenzhen 518060, China; Shenzhen Key Laboratory of New Information Display and Storage Materials, College of Materials Science and Engineering, Shenzhen University, Shenzhen 518060, China; Shenzhen Key Laboratory of New Information Display and Storage Materials, College of Materials Science and Engineering, Shenzhen University, Shenzhen 518060, China; Shenzhen Key Laboratory of New Information Display and Storage Materials, College of Materials Science and Engineering, Shenzhen University, Shenzhen 518060, China; College of Physics and Optoelectronic Engineering, Shenzhen University, Shenzhen 518060, China

**Keywords:** narrowband electroluminescence, thermally activated delayed fluorescence (TADF), multiple resonance emitters, ultra-high external quantum efficiency

## Abstract

Multi-boron-embedded multiple resonance thermally activated delayed fluorescence (MR-TADF) emitters show promise for achieving both high color-purity emission and high exciton utilization efficiency. However, their development is often impeded by a limited synthetic scope and excessive molecular weights, which challenge material acquisition and organic light-emitting diode (OLED) fabrication by vacuum deposition. Herein, we put forward a B‒N covalent bond-involved π-extension strategy via post-functionalization of MR frameworks, leading to the generation of high-order B/N-based motifs. The structurally and electronically extended π-system not only enhances molecular rigidity to narrow emission linewidth but also promotes reverse intersystem crossing to mitigate efficiency roll-off. As illustrated examples, ultra-narrowband sky-blue emitters (full-width at half-maximum as small as 8 nm in *n*-hexane) have been developed with multi-dimensional improvement in photophysical properties compared to their precursor emitters, which enables narrowband OLEDs with external quantum efficiencies (EQE_max_) of up to 42.6%, in company with alleviated efficiency decline at high brightness, representing the best efficiency reported for single-host OLEDs. The success of these emitters highlights the effectiveness of our molecular design strategy for advanced MR-TADF emitters and confirms their extensive potential in high-performance optoelectronic devices.

## Teaser

This research article introduces a chemical strategy to create advanced organic light-emitting materials with high efficiency and ultra-narrow emission bands, paving the way for more power-efficient displays with incredible color purity.

## INTRODUCTION

Narrowband organic emitting materials have recently gained considerable attention to meet the demands of high-definition organic light-emitting diodes (OLEDs) [1–3]. A promising approach involves the utilization of multiple resonance thermally activated delayed fluorescence (MR-TADF) materials [4]. Typically, an MR-TADF emitter features a flat polycyclic aromatic structure with electron-deficient boron (B) and electron-rich nitrogen (N) atoms strategically positioned in an *ortho*/*para* manner. This configuration not only promises narrow emission spectra with high photoluminescence quantum yields (*Φ*_PL_s) by reducing vibronic coupling between the ground (S_0_) and the lowest excited singlet (S_1_) states, but also facilitates atomically distributed frontier molecular orbitals (FMOs), resulting in a reduced singlet–triplet gap (Δ*E*_ST_). However, creating MR-TADF materials with ultranarrow emission bands remains challenging. Moreover, MR-TADF devices have typically suffered from significant triplet-involved bimolecular quenching processes, mainly due to inefficient reverse intersystem crossing (RISC) of the emitters, leading to substantial efficiency losses at high brightness levels [5–8].

**Scheme 1.**
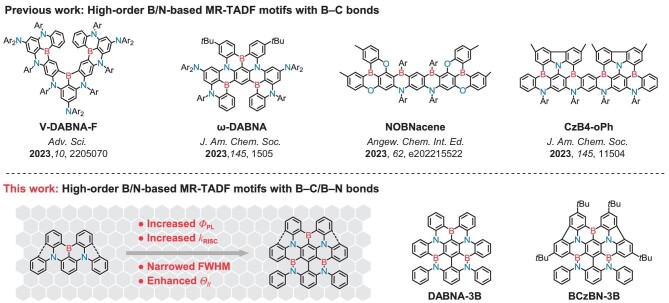
Molecular design concept.

In response to these challenges, recent efforts have focused on incorporating high-order B/N-based MR-TADF motifs containing multiple B atoms. This strategy has been demonstrated to further narrow the emission linewidth and reduce Δ*E*_ST_, thereby enhancing color purity and expediting RISC for MR-TADF emitters [9]. Such improvement is attributed to the promotion of short-range charge transfer (SRCT) and the suppression of structural relaxation and vibronic coupling within π-extended frameworks [10]. Leveraging this strategy, a wealth of research has aimed to fine-tune the optoelectronic properties of MR emitters, resulting in impressive full-width at half-maximum (FWHM) values, excellent external quantum efficiencies (EQE), and reduced efficiency roll-off at high brightness [11–21]. However, the design scope has primarily revolved around double B-embedded MR-TADF structures due to the synthetic challenges associated with multi-borylated products [22]. Thus far, there have been only a few instances of evaporation-type OLEDs based on MR-TADF emitters featuring three or more B atoms (Scheme 1). For instance, V-shaped and ω-shaped MR emitters containing three B atoms, namely V-DABNA-F and ω-DABNA, were reported by Hatakeyama *et al*., enabling the fabrication of vacuum-deposited blue and green OLEDs with maximum EQE (EQE_max_) values of up to 31.1% and very low efficiency roll-off [23,24]; linearly extended multi-borylated MR-TADF acene emitters such as α-3BNMes and NOBNacene were reported by Zysman-Colman *et al*., both of which enabled deep-blue emission (CIE_y_ ≤ 0.1) featuring high color purity [25,26]. More recently, Hatakeyama *et al*. developed a one-shot multiple borylation method for constructing acenes possessing four to eight B atoms, delivering ultra-narrowband sky-blue emission with FWHMs as small as 12–16 nm. However, the excessively high molecular weight disabled corresponding device characterization, in spite of the quadruple-borylated derivative CzB4-oPh [27]. For the construction of long-wavelength emitters, Zhang *et al*. hybridized *para* B-π-N and *para* B-π-B arranged patterns in a quadruple-borylated MR framework, affording orange-red electroluminescence with a high EQE_max_ of 35.8% [28]. Despite the above progress, it is clear that the conservative approach of introducing B atoms as B‒C bonds limited molecular design and increased the synthesis difficulty of multi-borylated MR-TADF emitters. Therefore, it is imperative to explore alternative architectures and synthetic methodologies for incorporating B-based fragments into molecular frameworks.

In this context, we wish to present a B‒N covalent bond-involved π-extension strategy that introduces three B atoms into the traditional B‒C bond-based MR-TADF framework. Recently, Duan *et al*. demonstrated that the inclusion of easily accessible B‒N/B‒O bonds could induce the MR property and achieve narrowband emission with desirable *Φ*_PL_ in a series of double-boron embedded emitters [29–31]. In this work, the B‒N bond-based fragments are installed via post-functional modification of the halogenated parent emitters **DABNA** and **BCzBN**. This approach offers high flexibility in molecular design, yielding novel high-order B/N-based MR-TADF motifs with compacted structures. The B‒N segments not only function as pincers to enhance molecular rigidity, but also act as fusion units to increase electronic delocalization and reduce Δ*E*_ST_. The proof-of-concept emitters, **DABNA-3B** and **BCzBN-3B**, featuring a compact MR-TADF framework with three B atoms and four N atoms, exhibit significant improvements across various metrics compared to their parent emitters, including increased *Φ*_PL_, accelerated RISC rate (*k*_RISC_), reduced FWHM of emission spectra, and enhanced horizontal orientation factors (*Θ*_//_). Consequently, OLEDs incorporating **DABNA-3B** and **BCzBN-3B** within a binary emitting system exhibit outstanding electroluminescence performance. They achieve high EQE_max_ values of 33.8% and 42.6%, respectively, and exhibit significantly mitigated efficiency roll-offs, along with FWHMs of 25 and 22 nm. This work not only establishes a new benchmark for device performance based on a binary emitting system but also opens up innovative avenues for the design of multi-borylated materials.

## RESULTS AND DISCUSSION

**Figure 1.**
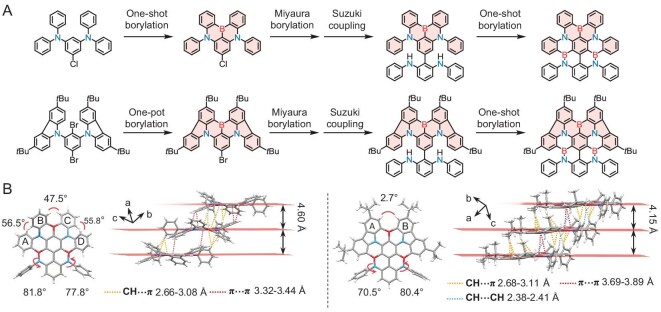
(A) Synthesis of **DABNA-3B** and **BCzBN-3B**. (B) Crystal structures and packing patterns of **DABNA-3B** and **BCzBN-3B**.

The synthetic routes toward the target compounds were established through sequential multiple borylations, as depicted in Fig. 1A, Schemes S1 and S2 (refer to Supporting Information for detailed synthesis). The initial critical step involved creating a halogenated **DABNA** or **BCzBN** core through borylation-annulation reactions. Subsequently, the 2,6-bis(phenylamino)phenyl group was incorporated using consecutive Miyaura borylation and Suzuki coupling reactions. The final products, **DABNA-3B** and **BCzBN-3B**, were obtained through a single-step, lithium-free borylation-annulation reaction with high yields. Both molecular structures were comprehensively characterized by nuclear magnetic resonance spectroscopy (^1^H NMR, ^13^C NMR, ^1^H-^1^H COSY and ROESY), high-resolution mass spectrometry (HRMS) and elemental analysis, as shown in Figs S1–S22 of the Supplementary data. The thermal properties of the emitters were investigated by using thermo-gravimetric analysis (TGA) and differential scanning calorimetry (DSC) (Fig. S23), revealing high decomposition temperatures (*T*_d_, corresponding to 5% weight loss) of 479°C for **DABNA-3B** and 518°C for **BCzBN-3B**, as well as glass transition temperatures (*T*_g_) above 300°C. This study presents a rare instance of post-synthetic annulative π-extension on MR frameworks, which leads to the efficient synthesis of higher-order MR emitters that are anticipated to facilitate the development of boron-doped graphenoid structures [24,32–34].

Single crystals of **DABNA-3B** and **BCzBN-3B** were obtained through the gradual evaporation of a toluene solution at ambient temperature [CCDC 2307956, 2307957]. X-ray crystallography revealed that both compounds crystallized in the triclinic *P*1 space group (Fig. 1B, Tables S1 and S2). Due to the presence of multiple [4]helicene moieties, **DABNA-3B** exhibited a quasi-planar configuration with partial twist at its molecular edges—the dihedral angle between the terminal phenyl rings of the N, B, N-based [4]helicene was 47.5°, while for the B, N, B-based [4]helicenes, the angles were 56.5° and 55.8°, respectively. According to previous studies, this twisted geometry was postulated to be conducive to an increased spin-orbit coupling (SOC) effect between singlet and triplet excited states, potentially enhancing the *k*_RISC_ [17,35,36]. Conversely, **BCzBN-3B** possessed a dihedral angle of only 2.7° between the terminal phenyl rings of its N, B, N-based [4]helicene, indicating a much flatter structure. For both emitters, the overall quasi-planar shaped molecular structures made them tend to align parallel on the substrate, which were conducive to higher *Θ*_//_ factors. In addition, they exhibited substantial dihedral angles between the suspended phenyl rings and the MR plane (70.5°–81.8°), which may weaken the intermolecular interactions between luminescent cores. As a result, we observed relatively large face-to-face distances for both **DABNA-3B** (4.60 Å) and **BCzBN-3B** (4.15 Å). Overall, these expansive intermolecular distances may reduce aggregation-induced quenching (ACQ) in the solid state for both **DABNA-3B** and **BCzBN-3B** [37–40].

**Figure 2.**
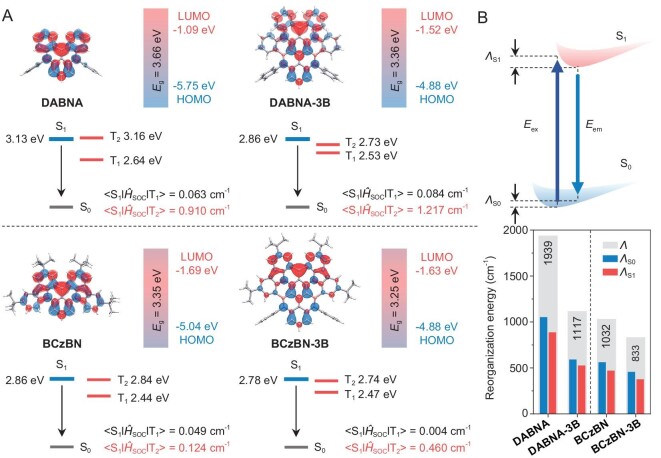
(A) Distributions of FMOs with energy gap (*E*_g_) values, as well as singlet/triplet state energy levels and SOC matrix elements. (B) Theoretical estimation of the total reorganization energies (*Λ*).

To get a deep insight into the structural and electronic properties of the emitters, density functional theory (DFT) and time-dependent DFT (TD-DFT) calculations were performed at B3LYP-D3(BJ)/6–31G(d, p) level (Tables S3–S11). Figure 2A illustrates the electron distributions of the highest occupied molecular orbital (HOMO) and the lowest unoccupied molecular orbital (LUMO) wave functions, which remained spatially alternative on the entire skeletons for **DABNA-3B** and **BCzBN-3B**, indicating their distinct MR feature. Since the non-bonding character hindered the effective conjugation length, the π-extension only slightly decreased the HOMO-LUMO energy gaps (*E*_g_) compared with those of the parent molecules (3.36 eV for **DABNA-3B** vs. 3.66 eV for **DABNA**; 3.25 eV for **BCzBN-3B** vs. 3.35 eV for **BCzBN**). The well-preserved short-range charge separation along with extended electronic delocalization jointly led to smaller Δ*E*_ST_ values of **DABNA-3B** and **BCzBN-3B** (0.33 and 0.31 eV) than those of parent molecules (0.49 and 0.42 eV), as revealed by TD-DFT calculations. Besides, it is noted that both **DABNA-3B** and **BCzBN-3B** presented high T_2_ states lying between S_1_ and T_1_ states. Despite the similar orbital parentage between S_1_ and T_1_, the natural transition orbital (NTO) distribution analysis suggested obvious orbital angular momentum variation in T_2_ states (Fig. S24), offering higher SOC matrix elements (SOC = <S_n_|*Ĥ*_SOC_|T_n_>) of S_1_–T_2_ than those of S_1_–T_1_. Therefore, the RISC should benefit from the second-order spin-vibronic coupling process. These results aligned well with recent theoretical studies that emphasized the importance of the high-lying triplet excited state in facilitating the energy upconversion of MR-TADF emitters [41,42]. Notably, the <S_1_|*Ĥ*_SOC_|T_2_> values of **DABNA-3B** and **BCzBN-3B** (1.217 and 0.460 cm^−1^) were much higher than those of **DABNA** and **BCzBN** (0.910 and 0.124 cm^−1^), which would give rise to enhanced *k*_RISC_ values.

To further investigate the impact of B‒N bond-based fusion heterocycles on the structural relaxation during the excitation-emission process, the reorganization energy (*Λ*) of **DABNA-3B** and **BCzBN-3B** were calculated and analyzed using the Molecular Materials Property Prediction Package (MOMAP), and compared with those of **DABNA** and **BCzBN**. As shown in Fig. 2B, the B‒N bond-involved π-extension leads to a remarkable decrease of *Λ* (1117 cm^−1^ for **DABNA-3B** vs. 1939 cm^−1^ for **DABNA**; 833 cm^−1^ for **BCzBN-3B** vs. 1032 cm^−1^ for **BCzBN**), indicative of the suppressed geometry changes during the transitions. The relationships of *Λ* with vibrational modes for the S_1_→S_0_ transition are further depicted in Fig. S25. Obviously, the incorporation of supplemental fusing moieties diminished the vibration strength at high-frequency regions (above 500 cm^−1^), which represents the suppressed structural deformation on a molecular level as well as atomic level. These above results indicate that the fusion of B‒N bond-based heterocycles into the parent cores could effectively enhance molecular rigidity and consequently narrow the emissive bandwidth.

**Figure 3.**
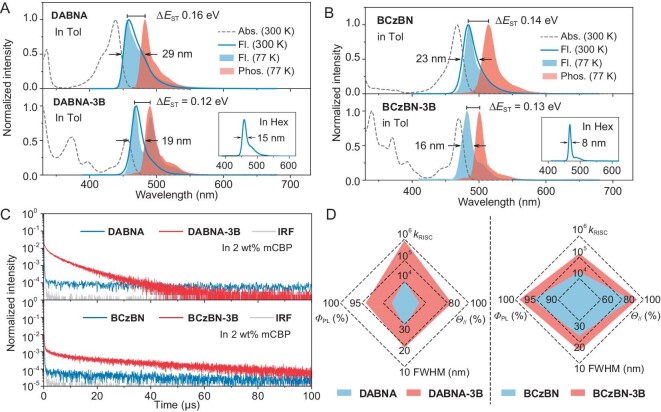
Absorption (Abs.: 300 K), fluorescence (Fl.: 300 and 77 K) and phosphorescence (Phos.: 77 K) spectra of (A) **DABNA** and **DABNA-3B** as well as (B) **BCzBN** and **BCzBN-3B** in toluene solutions at 1.0 × 10^−5^ mol L^−1^, with fluorescence spectra recorded in *n*-hexane solutions at 1.0 × 10^−5^ mol L^−1^ as the inset. (C) Transient PL spectra (300 K) of 2 wt% emitter: mCBP films, with IRF (instrument response function) for comparison. (D) Radar diagram showing the key photophysical properties of the emitters (the outer area is better). FWHM = full-width at half-maximum (in toluene); *k*_RISC_ = reverse intersystem crossing rate (in mCBP); *Φ*_PL_ = photoluminescence quantum yield (in mCBP); *Θ*_//_ = horizontal orientation factors (in corresponding emitting layer).

The photophysical properties of the emitters were examined in dilute toluene solution (1.0 × 10^−5^ mol L^−1^), as shown in Fig. 3. Compared to their respective parent emitters, the absorption spectra of **DABNA-3B** and **BCzBN-3B** exhibited more pronounced n-π*/π-π* transitions below 420 nm. However, they maintained similarly intense short-range charge transfer (SRCT) bands above 420 nm, peaking at 455 and 470 nm for **DABNA-3B** and **BCzBN-3B**, respectively. The optical band gaps (*E*_g_) were estimated at 2.62 eV for **DABNA-3B** and 2.55 eV for **BCzBN-3B**, based on the absorption threshold. These values were slightly lower compared to those of **DABNA** and **BCzBN**, which aligned with theoretical predictions. The fluorescence spectra showed sky-blue emissions with peak wavelengths at 470 and 482 nm for **DABNA-3B** and **BCzBN-3B**, respectively. The relatively small Stokes shifts, 15 nm for **DABNA-3B** and 12 nm for **BCzBN-3B**, suggested minimal molecular conformational relaxation between S_1_ and S_0_, corroborating the calculated results mentioned above. Additionally, both **DABNA-3B** and **BCzBN-3B** exhibited extremely narrow FWHM values of 19 nm/0.11 eV and 16 nm/0.09 eV, respectively. These narrowed emission spectra, compared to the parent molecules (29 nm/0.17 eV for **DABNA** and 23 nm/0.12 eV for **BCzBN**), implied significantly reduced structural vibrations due to enhanced rigidity. When different solvents were used, ranging from *n*-hexane to acetonitrile, the new emitters demonstrated similar solvatochromic effects with only slight bathochromic shifts in emission and a modest increase in FWHM, as shown in Fig. S26 and Table S12. This indicated that the introduction of the B‒N bond-based fusing unit did not alter the MR characteristics. Of particular note, the spectral FWHM of **BCzBN-3B** in an *n*-hexane solution was as narrow as 8 nm/0.05 eV (Fig. 3B), representing one of the narrowest emission bands observed for pure organic emitters. Based on the fluorescence and phosphorescence maxima at 77 K, the Δ*E*_ST_ values for **DABNA-3B** and **BCzBN-3B** were determined to be 0.13 and 0.10 eV, respectively, which were smaller than those of the parent molecules (0.15 eV for **DABNA** and 0.14 eV for **BCzBN**). Such small Δ*E*_ST_s, along with the potential for spin-vibronic coupling mechanisms typical of organoboron emitters, were expected to promote the exciton upconversion from T_1_ to S_1_.

To delve into the solid-state photophysical characteristics, we prepared doped films by blending the emitters into a host matrix of 3,3’-bis(carbazol-9-yl)biphenyl (mCBP) with an optimized doping concentration of 2 wt%. This ratio ensured efficient energy transfer while minimizing bimolecular collisions. The fluorescence spectra of these films, as depicted in Fig. S27, exhibited a modest redshift and slight broadening in FWHM, 28 nm/0.15 eV for **DABNA-3B** and 24 nm/0.12 eV for **BCzBN-3B**, compared to the spectra in solution, likely due to the increased polarity of the host matrix. Notably, the films containing B‒N-embedded emitters demonstrated higher *Φ*_PL_ compared to their parent emitters (94% for **DABNA-3B** vs 88% for **DABNA**; 99% for **BCzBN-3B** vs 96% for **BCzBN**). Such increase was attributed to reduced nonradiative decay from suppressed structural vibrations. The temperature-dependent transient photoluminescence (PL) spectra of the doped films demonstrated pronounced TADF behavior of the emitters, with delayed fluorescence components intensifying as temperatures rose (Fig. S28). At 300 K, the delayed fluorescence lifetimes for **DABNA-3B** and **BCzBN-3B** were significantly reduced to 6.10 μs and 25.4 μs, respectively, compared to their parent molecules **DABNA** (93.7 μs) and **BCzBN** (67.0 μs) (Fig. 3C, Table S13). Based on the *Φ*_PL_s and transient PL decay profiles, the key rate constants were estimated and listed in Table 1. The emitters displayed notably high radiative decay rates (*k*_r_), within the range of 3.80 × 10^7^ to 1.50 × 10^8^ s^−1^, in alignment with the desirable attributes of MR-TADF emitters. Concurrently, the non-radiative decay rates for **DABNA-3B** and **BCzBN-3B** were substantially lower than their parent compounds, attributed to increased molecular rigidity and lower reorganization energy. More importantly, the calculated *k*_RISC_ rates for **DABNA-3B** and **BCzBN-3B** were 8.63 × 10^5^ s^−1^ and 1.46 × 10^5^ s^−1^, respectively. These rates signified improvements of 77-fold for **DABNA-3B** and 7-fold for **BCzBN-3B** over their parent molecules (0.11 × 10^5^ s^−1^ for **DABNA**; 0.20 × 10^5^ s^−1^ for **BCzBN**). The enhanced spin-flipping exciton conversion can be credited to the combined effect of the small Δ*E*_ST_ and large SOC effects, corroborated by theoretical predictions. Remarkably, the *k*_RISC_ of **DABNA-3B** approached 10^6^ s^−1^, which surpassed the benchmark deep-blue emitter *ν*-DABNA (2.0 × 10^5^ s^−1^) and represented one of the highest reported values for MR-TADF molecules without heavy atom effect (Table S14) [9,43]. This rapid triplet dynamics underscored the impact of molecular geometry distortion and significant SOC values, as supported by crystal structure analysis and theoretical outcomes. In Fig. 3D, the radar diagram succinctly compared the photophysical properties of the emitters, where a more extensive outer area corresponded to better performance. The properties charted included FWHM, *Φ*_PL_, *k*_RISC_, and *Θ*_//_ (discussed further below). The radar diagram clarified that **DABNA-3B** and **BCzBN-3B** outclassed **DABNA** and **BCzBN** in all measured aspects, confirming that the photophysical superiority was a direct result of the B‒N covalent bond-involved π-extension.

**Table 1.** Summary of photophysical properties.

Compound	*λ*_abs_^a^ [nm]	*λ*_em_^a^ [nm]	FWHM^a^ [nm/eV]	∆*E*_ST_^a^ [eV]	*Φ*_PL_^b^ [%]	*k*_r_^b^ [10^7^ s^−1^]	*k*_nr_^b^ [10^6^ s^−1^]	*k*_RISC_^b^ [10^5^ s^−1^]
**DABNA**	439	458	29/0.17	0.15	88	9.60	13.09	0.11
**DABNA-3B**	455	470	19/0.11	0.13	94	9.98	6.37	8.63
**BCzBN**	467	484	23/0.12	0.14	96	14.79	6.16	0.20
**BCzBN-3B**	470	482	16/0.09	0.10	99	3.80	0.38	1.46

a Peak of absorption (*λ*_abs_), fluorescence (*λ*_em_, 300 K) spectra, full-width at half-maximum (FWHM) of fluorescence, and S_1_-T_1_ energy gap (Δ*E*_ST_) measured in 1 × 10^−5^ mol L^−1^ toluene solutions. ^b^Absolute photoluminescence quantum yield (*Φ*_PL_), rate constants of singlet radiative decay (*k*_r_), non-radiative decay (*k*_nr_), reverse intersystem crossing (*k*_RISC_) measured in 2 wt% emitter: mCBP films; note: the quantum yield and lifetimes were measured under deoxygenated conditions.

**Figure 4.**
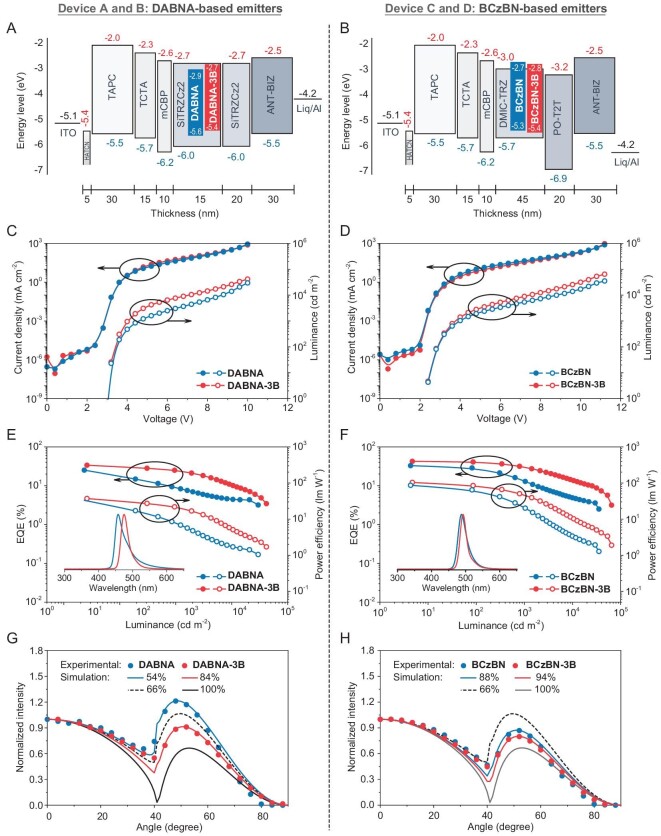
Device structures and energy level diagrams of (A) **DABNA**-core emitters and (B) **BCzBN**-core emitters. (C) Current density-voltage-luminance curves and (E) EQE-luminance and PE-luminance curves (inset: EL spectra at a luminance of 1000 cd m^−2^) of **DABNA**-core emitter-based OLEDs. (D) Current density-voltage-luminance curves and (F) EQE-luminance and PE-luminance curves (inset: EL spectra at a luminance of 1000 cd m^−2^) of **BCzBN**-core emitter-based OLEDs. Angle-dependent PL spectra for (G) **DABNA**-core emitters doped in SiTrzCz2 and (H) **BCzBN**-core emitters doped in DMIC-TRZ, compared with simulated curves (lines) with different horizontal dipole ratios *Θ*_//_. *Θ*_//_ = 100% for fully horizontal dipoles, and 66% for the isotropic dipole orientation.

We subsequently examined the electroluminescent (EL) performances using devices with a single host. The optimized device configuration comprised the following layers: indium tin oxide (ITO)/dipyrazino[2,3-f:2',3'-h]quinoxaline-2,3,6,7,10,11-hexacarbonitrile (HAT-CN, 5 nm)/1,1-bis[(di-4-tolylamino)phenyl]cyclohexane (TAPC, 30 nm)/tris(4-carbazolyl-9-ylphenyl)amine (TCTA, 15 nm)/mCBP (10 nm)/emissive layer (EML)/hole blocking layer (HBL, 20 nm)/1-(4-(10-([1,1′-biphenyl]-4-yl)anthracen-9-yl)phenyl)-2-ethyl-1H-benzo[d]imidazole (ANT-BIZ, 30 nm)/8-hydroxyquinolinato lithium (Liq, 2 nm)/Al. Specifically, for devices utilizing **DABNA** and **DABNA-3B**, the EML and HBL comprised 2 wt% emitters in 9,9'-(6-(3-(triphenylsilyl)phenyl)-1,3,5-triazine-2,4-diyl)bis(9H-carbazole) (SiTrzCz2) (15 nm) and SiTrzCz2 (20 nm), respectively. In contrast, devices employing **BCzBN** and **BCzBN-3B** contained EMLs and HBLs of 2 wt% emitters in 5-(3-(4,6-diphenyl-1,3,5-triazin-2-yl)phenyl)-7,7-dimethyl-5,7-dihydroindeno[2,1-*b*]carbazole (DMIC-TRZ) (45 nm) and tris(benzene-3,1-diyl)tris(diphenylphosphine oxide) (PO-T2T, 20 nm), respectively. The chosen host materials, SiTrzCz2 [44] and DMIC-TRZ [45], exhibited well-aligned HOMO/LUMO energy levels with adjacent layers, suitable triplet energy, and favorable spectral overlap between host emission and dopant absorption. The concentration was precisely optimized within a low doping level to avoid undesirable concentration quenching, and the PL profiles of the EMLs basically resembled those of the corresponding mCBP films (Fig. S29). The OLED structures, device performances and the molecular structures of materials used in OLEDs are depicted in Fig. 4A–F and Fig. S30, with key performance data summarized in Table 2.

**Table 2.** Summary of OLED performances.

Emitter	*V*_on_^a^ [V]	*λ*_EL_^b^ [nm]	FWHM^c^ [nm/eV]	*L*_max_^d^ [cd m^−2^]	EQE_max/100/1000_^e^ [%]	PE_max/100/1000_^e^ [lm W^−1^]	CE_max/100/1000_^e^ [cd A^−1^]
**DABNA**	3.0	457	35/0.20	29 693	25.3/19.9/9.2	33.2/21.3/7.8	33.8/28.1/11.5
**DABNA-3B**	3.2	475	25/0.14	41 634	33.8/30.6/25.1	37.2/32.3/21.7	78.7/76.6/57.0
**BCzBN**	2.4	488	30/0.15	35 363	33.0/27.4/13.1	86.6/60.8/20.5	66.2/55.4/26.3
**BCzBN-3B**	2.4	493	22/0.11	64 666	42.6/40.4/30.5	103.8/83.6/46.4	79.3/75.1/55.0

a Turn-on voltage at 1 cd m^−2^. ^b^Electroluminescence peak wavelength. ^c^Full-width at half-maximum. ^d^Maximum luminance. ^e^External quantum efficiency, power efficiency and current efficiency: maximum, values at 100 and 1000 cd m^−2^.

Benefitting from the balanced carrier transporting ability of the host materials, all the devices exhibited low turn-on voltages (*V*_on_s, 3.0–3.2 V for **DABNA** and **DABNA-3B**; 2.4–3.2 V for **BCzBN** and **BCzBN-3B**) and fairly high luminance of over 29 000 cd m^−2^. The EL spectra displayed high color stability with minimal change in the range between 3.2 V and 8.0 V (Fig. S31), confirming the efficient host-to-emitter energy transfer and the fixation of radiative transition excitons on the emitters. Consistent with the corresponding PL spectra (Fig. S29), the devices displayed EL peaks at 475 nm and 493 nm with FWHM of 25 nm/0.14 eV and 22 nm/0.11 eV for **DABNA-3B** and **BCzBN-3B**, respectively, narrower than their parent molecules (**DABNA**, 35 nm/0.20 eV; **BCzBN**, 30 nm/0.15 eV). Notably, devices based on **DABNA-3B** and **BCzBN-3B** achieved high EQE_max_ values of 33.8% and 42.6%, power efficiency (PE_max_) of 37.2 and 103.8 lm W^−1^, and current efficiency (CE_max_) of 78.7 and 79.3 cd A^−1^, respectively. These metrics indicated a significant enhancement over their corresponding parent emitters (EQE_max_ of 25.3% and 33.0%, PE_max_ of 33.2 and 86.6 lm W^−1^, and CE_max_ of 33.8 and 66.2 cd A^−1^ for **DABNA** and **BCzBN**, respectively). In particular, **BCzBN-3B-**based devices portrayed the highest efficiency for TADF emitters in the binary emitting system, to the best of our knowledge (Table S15). To gain further insight into the excellent EL performance, angle-dependent PL spectra of the EMLs were measured to probe the orientation of the emitters. As displayed in Fig. 4G and H, the **DABNA-3B** and **BCzBN-3B** manifested higher *Θ*_//_ values than those of the parent molecules (84% for **DABNA-3B** vs 54% for **DABNA**; 94% for **BCzBN-3B** vs 88% for **BCzBN**). The preferential horizontal dipole orientation could be attributed to the enlarged molecular planarity (Fig. S32) and the higher *T*_g_ values that stabilized the molecular/dipole orientation during film deposition [46,47]. These factors, combined with the near-unity *Φ*_PL_ (99%) of the emitter, as well as the potential sensitizing ability of the DMIC-TRZ host, underpinned the extraordinary efficiency of over 40% achieved by **BCzBN-3B**-based devices.

Additionally, the devices incorporating **DABNA-3B** and **BCzBN-3B** emitters demonstrated lower efficiency declines at high brightness, with EQE_1000_ values (EQE at 1000 cd m^–2^) of 25.1% and 30.5%, respectively. This performance translated to a reduction in efficiency of 25.7% and 28.4%, contrasting sharply with the 63.6% and 60.3% reductions observed in devices with **DABNA** and **BCzBN** at the same luminance level. To elucidate the underlying mechanism of the efficiency roll-off, we modeled the EQE-current density behavior using theories of triplet-triplet annihilation (TTA) and triplet-polaron annihilation (TPA), as shown in Fig. S33. The models highlighted TTA as a significant factor affecting the efficiency roll-off. The critical current density (*J*_0_, the point at which efficiency drops to half due to TTA), was significantly higher in the **DABNA-3B** device (20.1 mA cm^−2^) compared to those with **DABNA** (4.1 mA cm^−2^). A similar trend was seen with **BCzBN-3B** (9.8 mA cm^−2^) vs **BCzBN** (2.5 mA cm^−2^). Hence, the diminished efficiency roll-off was clearly associated with the fast depletion of triplet excitons, benefitting from the efficient RISC process. These findings were a testament to the efficacy of our molecular design strategy.

## CONCLUSION

In summary, we put forward a B‒N covalent bond-involved π-extension strategy to construct multi-boron-embedded MR-TADF emitters. Synthetically, our approach marks a noteworthy example of post-synthetic annulative π-extension applied to MR frameworks. In terms of emission properties, our method enhances the spin-flip process via extended electronic delocalization and achieves narrower emission linewidth due to increased molecular rigidity. Utilizing this design, the sky-blue MR-TADF emitters **DABNA-3B** and **BCzBN-3B** exhibited outstanding photophysical performance, including FWHMs narrower than the state-of-the-art quantum dots (as small as 8 nm/0.05 eV in non-polar solvent), nearly perfect *Φ*_PL_, accelerated RISC process, and predominantly horizontal orientation of emitting dipoles. The exceptional property was further evidenced by their impressive electroluminescence efficiencies and reduced efficiency roll-off in OLEDs with binary EMLs, achieving EQE_max/1000_ of 33.8%/25.1% and 42.6%/30.5%, respectively, alongside with small FWHM values. This study not only paves the way for innovative molecular design approaches but also holds promise for further advancements in MR-TADF emitters and the continued evolution of high-performance narrowband OLEDs.

## METHODS

The detailed preparation and characterization methods of materials are available as Supplementary data.

## Supplementary Material

nwae115_Supplemental_File

## Data Availability

The data that support the findings of this study are available from the corresponding author upon reasonable request.
