# Supplementary material for: B‒N covalent bond-involved π-extension of multiple resonance emitters enables high-performance narrowband electroluminescence
Source: Natl Sci Rev. 2024 Mar 23;11(6):nwae115. doi: 10.1093/nsr/nwae115 (PMC11067958; doi:10.1093/nsr/nwae115)
Supplement: nwae115_Supplemental_File [file nwae115_supplemental_file.docx]

**Supporting Information**

**B‒N Covalent Bond Involved π-Extension of Multiple Resonance Emitters Enables High-Performance Narrowband Electroluminescence**

Xingyu Huang^+^, Jiahui Liu^+^, Yulin Xu, Guohao Chen, Manli Huang, Mingxin Yu, Xialei Lv, Xiaojun Yin, Yang Zou, Jingsheng Miao, Xiaosong Cao*, Chuluo Yang*

*Corresponding Author(s): xcao@szu.edu.cn (Xiaosong Cao), clyang@szu.edu.cn (Chuluo Yang).

^+^These authors contributed equally to this work.

**Table of Contents**

[1. Experimental Section 2](#_Toc146029345)

[1.1. Materials and Methods 2](#_Toc146029346)

[1.2. Synthesis 2](#_Toc146029347)

[1.3. Single Crystal X-ray Crystallography 6](#_Toc146029348)

[1.4. Quantum Chemical Calculations 6](#_Toc146029349)

[1.5. Thermal and Electrochemical Characterization 7](#_Toc146029350)

[1.6. Photophysical Characterization 7](#_Toc146029351)

[1.7. Analysis of Rate Constants 7](#_Toc146029352)

[1.8. Device Fabrication and Measurement 8](#_Toc146029353)

[1.9. Determination of Emitting Dipole Orientation 8](#_Toc146029353)

[2. Figures and Tables 9](#_Toc146029354)

[3. References 50](#_Toc146029355)

# 1. Experimental Section

## 1.1. Materials and Methods

All raw materials and anhydrous solvents were commercially available and used without further purification. The reactions were carried out under the protection of the high-purity argon atmosphere. All reactions were heated by metal sand bath (WATTCAS, LAB-500, https://www.wattcas.com). NMR measurements were conducted on Bruker Advance 500 (or 600) spectrometer. Tetramethylsilane was used as the internal standard and CDCl_3_ as the solvent. The high-resolution mass spectrometry (HRMS) for the compounds was conducted on a Thermo Scientific LTQ Orbitrap XL with ESI ion source. Elemental analysis was performed on a Vario EL cube elemental analyzer.

## 1.2. Synthesis

**Scheme S1.** Synthetic route of **DABNA-3B**.

***Synthesis of compound 1-1*:** A 100 mL Schlenk tube with a magnetic stir bar was charged with 1,3-dibromo-5-chlorobenzene (5.40 g, 20.0 mmol), diphenylamine (7.40 g, 44.0 mmol), Pd(OAc)_2_ (0.23 g, 1.0 mmol), *t*Bu_3_PHBF_4_ (0.87 g, 3.0 mmol), *t*BuONa (5.70 g, 60.0 mmol) and toluene (50 mL) under argon atmosphere. The resulting mixture was stirred at 110 °C for 18 h. After cooling to room temperature, the solvent was removed under vacuum and the residue was purified by column chromatography on silica gel using petroleum ether/dichloromethane (7/1, *v/v*) as eluent to give the compound **1-1** as a white solid (5.95 g, 67%). ^1^H NMR (500 MHz, CDCl_3_): *δ* (ppm) 7.23 (td, *J* = 7.5, 1.8 Hz, 8H), 7.07-7.04 (m, 8H), 7.01 (t, *J* = 7.3 Hz, 4H), 6.65 (t, *J* = 2.0 Hz, 1H), 6.57 (d, *J* = 2.0 Hz, 2H). ^13^C NMR (125 MHz, CDCl_3_): *δ* (ppm) 147.06, 135.23, 129.41, 124.78, 123.53, 116.70, 115.91.

***Synthesis of compound 1-2*:** An oven-dried Schlenk tube with a magnetic stir bar was charged with compound **2-1** (2.23 g, 5.0 mmol) and dry 1,2-dichlorobenzene (25.0 mL), and then BBr_3_ (1.93 mL, 20.0 mmol) was carefully added at room temperature under argon atmosphere. The reaction mixture was stirred at 200 °C for 24 h. After cooling to room temperature, 50.0 mL ethanol was added to quench the reaction. The mixture was filtered and the solid was washed by ethanol. The crude product **1-2** was dried under vacuum and was directly used in the next step without further purification. Yellow solid (1.67 g, 74%). ^1^H NMR (500 MHz, CDCl_3_): *δ* (ppm) 8.94 (d, *J* = 7.6 Hz, 2H), 7.72 (t, *J* = 7.7 Hz, 4H), 7.63 (t, *J* = 7.5 Hz, 2H), 7.45 (t, *J* = 7.7 Hz, 2H), 7.38 (d, *J* = 7.5 Hz, 4H), 7.30 (t, *J* = 7.3 Hz, 2H), 6.75 (d, *J* = 8.6 Hz, 2H), 6.13 (s, 2H). ^13^C NMR (125 MHz, CDCl_3_): *δ* (ppm) 147.82, 147.60, 141.86, 138.41, 135.14, 131.43, 131.27, 130.45, 129.07, 120.55, 117.36, 108.20, 105.35.

***Synthesis of compound 1-3*:** A 100 mL Schlenk tube with a magnetic stir bar was charged with compound **1-2** (1.36 g, 3.0 mmol), B_2_Pin_2_ (1.52 g, 6.0 mmol), Pd_2_(dba)_3_ (0.14 g, 0.15 mmol), X-Phos (0.14 g, 0.30 mmol), AcOK (0.88 g, 9.0 mmol) and 1,4-dioxane (25.0 mL) under argon atmosphere. The resulting mixture was stirred at 110 °C for 12 h. After cooling to room temperature, the mixture was filtered and the solid was washed by methanol and petroleum ether. The crude product **1-3** was dried under vacuum and was directly used in the next step without further purification. Green solid (1.50 g, 91%). ^1^H NMR (500 MHz, CDCl_3_): *δ* (ppm) 8.92 (d, *J* = 8.9 Hz, 2H), 7.72 (t, *J* = 7.7 Hz, 4H), 7.61 (t, *J* = 7.5 Hz, 2H), 7.41 (t, *J* = 7.4 Hz, 6H), 7.27 (s, 1H), 6.73 (d, *J* = 8.6 Hz, 2H), 6.60 (s, 2H), 1.17 (s, 12H). ^13^C NMR (125 MHz, CDCl_3_): *δ* (ppm) 147.98, 146.20, 142.38, 135.21, 131.17, 131.11, 130.79, 128.62, 119.89, 117.22, 110.82, 83.76, 24.81.

***Synthesis of compound 1-4***: A 100 mL Schlenk tube with a magnetic stir bar was charged with **1-3** (0.66 g, 1.2 mmol), compound **3** (0.34 g, 1.0 mmol), Pd(PPh_3_)_4_ (58 mg, 0.05 mmol), K_2_CO_3_ (0.55 g, 4.0 mmol) and toluene/ethanol/H_2_O (15.0 mL/5.0 mL/5.0 mL) under argon atmosphere. The resulting mixture was stirred at 80 °C for 12 h. After cooling to room temperature, the solvent was removed under vacuum. The crude product was recrystallized from dichloromethane and methanol to afford the compound **1-4** as a yellow solid (0.33 g, 49%). ^1^H NMR (600 MHz, CDCl_3_): *δ* (ppm) 8.96 (d, *J* = 7.7 Hz, 2H), 7.46-7.37 (m, 8H), 7.33-7.28 (m, 2H), 7.21 (td, *J* = 8.3, 7.5, 2.1 Hz, 8H), 7.04 (td, *J* = 8.1, 2.1 Hz, 1H), 6.97-6.91 (m, 2H), 6.90-6.85 (m, 4H), 6.82 (dd, *J* = 8.1, 2.8 Hz, 2H), 6.75 (dd, *J* = 8.6, 2.8 Hz, 2H), 6.18 (d, *J* = 3.2 Hz, 2H), 5.46 (s, 2H). ^13^C NMR (125 MHz, CDCl_3_): *δ* (ppm) 148.04, 147.70, 143.55, 141.83, 141.49, 138.14, 135.18, 131.22, 130.15, 129.31, 128.67, 128.22, 121.72, 120.39, 118.40, 117.31, 110.02, 106.89, 53.57.

***Synthesis of DABNA-3B***: An oven-dried Schlenk tube with a magnetic stir bar was charged with compound **1-4** (0.27 g, 0.4 mmol) and dry 1,2-dichlorobenzene (20.0 mL), and then BBr_3_ (0.31 mL, 3.2 mmol) was carefully added at room temperature under argon atmosphere. After stirring for 15 minutes, Et_3_N (0.44 mL, 3.2 mmol) was added. The reaction mixture was stirred at 180 °C for 36 h. After cooling to room temperature, 50.0 mL ethanol was added to quench the reaction. The solvent was removed under vacuum and the crude product was purified by column chromatography on silica gel using petroleum ether/dichloromethane (5/1, *v/v*) as eluent to give the product **DABNA-3B** as a yellow solid (0.11 g, 40%). ^1^H NMR (500 MHz, CDCl_3_): *δ* (ppm) 8.74 (d, *J* = 7.2 Hz, 2H), 8.19 (d, *J* = 8.2 Hz, 2H), 8.12 (d, *J* = 8.5 Hz, 2H), 7.74 (s, 4H), 7.68-7.61 (m, 4H), 7.59-7.48 (m, 6H), 7.36 (dt, *J* = 25.8, 7.8 Hz, 3H), 6.93 (t, *J* = 7.4 Hz, 2H), 6.84 (d, *J* = 8.3 Hz, 2H), 6.71 (d, *J* = 7.7 Hz, 2H). ^13^C NMR (125 MHz, CDCl3): *δ* (ppm) 148.86, 148.18, 147.16, 145.87, 145.80, 144.24, 134.11, 132.92, 131.40, 130.67, 130.40, 130.22, 129.85, 129.34, 128.74, 128.28, 128.04, 123.95, 123.77, 123.64, 122.74, 116.19, 109.50. HRMS: (ESI) m/z calcd for C_64_H_58_B_4_N_3_ [M+H]^+^: 695.2744; found: 695.2754. Elemental analysis: Calc. for C_48_H_29_B_3_N_4_: C, 83.05%; H, 4.21%; N, 8.07%; found: C, 83.09%; H, 4.27%; N, 8.02%.

***Synthesis of compound 3***: A 100 mL Schlenk tube with a magnetic stir bar was charged with 1,2,3-tribromobenzen (4.72 g, 15.0 mmol), aniline (2.93 g, 31.5 mmol), Pd_2_(dba)_3_ (0.28 g, 0.3 mmol), BINAP (1.4 g, 2.25 mmol), *t*BuONa (4.32 g, 45.0 mmol) and toluene (40.0 mL) under argon atmosphere. The resulting mixture was stirred at 110 °C for 14 h. After cooling to room temperature, the solvent was removed under vacuum and the crude product was purified by column chromatography on silica gel using petroleum ether/ethyl acetate (20/1, *v/v*) as eluent to give the compound **3** as a white solid (3.00 g, 59%). ^1^H NMR (500 MHz, CDCl_3_): *δ* (ppm) 7.36-7.30 (m, 4H), 7.18 (d, *J* = 7.6 Hz, 4H), 7.02 (dt, *J* = 19.5, 7.7 Hz, 3H), 6.81 (d, *J* = 8.1 Hz, 2H), 6.09 (s, 2H). ^13^C NMR (125 MHz, CDCl_3_): *δ* (ppm) 142.57, 141.97, 129.53, 127.92, 122.76, 120.69, 107.82, 102.85.

**Scheme S2.** Synthetic route of **BCzBN-3B**.

***Synthesis of compound 2-1***: A dried round bottom flask with a magnetic stir bar was charged with 3,6-di-*tert*-butyl-9*H*-carbazole (6.7 g, 24.0 mmol), 2,5-dibromo-1,3-difluorobenzene (3.08 g, 10.0 mmol), Cs_2_CO_3_ (9.38 g, 28.8 mmol) and 100.0 mL of *N,N*-dimethylformamide (DMF). The reaction mixture was stirred for 12 h at 150 °C. After cooling to room temperature, the mixture was quenched with water and extracted with dichloromethane. The combined organic phase was concentrated and the residue was purified by column chromatography on silica gel using petroleum ether/dichloromethane (10/1, *v/v*) as eluent to give the product **2-1** as a white solid.

***Synthesis of compound 2-2***: In an oven-dried Schlenk tube with a magnetic stir bar, a solution of *n*BuLi in *n*-hexane (4.0 mL, 2.5 M, 10.0 mmol) was added slowly to a solution of compound **2-1** (3.95 g, 5.0 mmol) in mesitylene (50.0 mL) at 0 °C under an argon atmosphere. After stirring for 1 h, the reaction mixture was cooled to -40 °C. After addition of BBr_3_ (2.0 mL, 20.0 mmol), the reaction mixture was stirred at room temperature for 2 h. Subsequently, *N,N*-diisopropylethylamine (3.5 mL, 20.0 mmol) was added at 0 °C and then the reaction mixture was allowed to warm to 180 °C and stirred for 12 h. After cooling to room temperature, the mixture was quenched with water, and extracted with dichloromethane. Then, the organic phases were concentrated and the residue was purified by column chromatography on silica gel using petroleum ether/dichloromethane (10/1, *v/v*) as eluent to give the product **2-2** as a yellow solid.

***Synthesis of compound 2-3***: A 100 mL Schlenk tube with a magnetic stir bar was charged with compound **2-2** (2.16 g, 3.0 mmol), B_2_Pin_2_ (1.52 g, 6.0 mmol), Pd(dppf)_2_Cl_2_ (0.11 g, 0.15 mmol), X-Phos (0.14 g, 0.30 mmol), AcOK (0.88 g, 9.0 mmol) and 1,4-dioxane (25.0 mL) under argon atmosphere. The resulting mixture was stirred at 80 °C for 12 h. After cooling to room temperature, the mixture was filtered and the solid was washed by methanol and petroleum ether. The crude product **2-3** was dried under vacuum and was directly used in the next step without further purification. Green solid.

***Synthesis of compound 2-4***: A 100 mL Schlenk tube with a magnetic stir bar was charged with compound **2-3** (0.77 g, 0.96 mmol), compound **3** (0.27 g, 0.80 mmol), Pd(PPh_3_)_4_ (46 mg, 0.04 mmol), K_2_CO_3_ (0.44 g, 3.20 mmol) and toluene/ethanol/H_2_O (15.0 mL/5.0 mL/5.0 mL) under argon atmosphere. The resulting mixture was stirred at 80 °C for 12 h. After cooling to room temperature, the solvent was removed under vacuum and then purified by column chromatography on silica gel using petroleum ether/dichloromethane (5/1, *v/v*) as eluent to give the compound **2-4** as a yellow solid (0.44 g, 61%). ^1^H NMR (600 MHz, CDCl_3_): *δ* (ppm) 9.13 (d, *J* = 1.7 Hz, 2H), 8.47 (d, *J* = 1.7 Hz, 2H), 8.33 (s, 2H), 8.22 (d, *J* = 2.0 Hz, 2H), 8.04 (d, *J* = 8.8 Hz, 2H), 7.40 (dd, *J* = 8.7, 2.0 Hz, 2H), 7.32 (t, *J* = 8.2 Hz, 1H), 7.22 (t, *J* = 7.9 Hz, 4H), 7.13 (d, *J* = 8.2 Hz, 2H), 7.08 (d, *J* = 7.8 Hz, 4H), 6.91 (t, *J* = 7.3 Hz, 2H), 5.67 (s, 2H), 1.68 (s, 18H), 1.49 (s, 18H). ^13^C NMR (125 MHz, CDCl_3_): *δ* (ppm) 145.66, 145.41, 145.11, 143.48, 142.30, 141.69, 139.69, 138.20, 129.91, 129.47, 129.07, 127.11, 124.76, 123.99, 121.58, 121.04, 119.05, 117.28, 114.26, 110.49, 109.85, 35.35, 34.88, 32.32, 31.91.

***Synthesis of BCzBN-3B***: An oven-dried Schlenk tube with a magnetic stir bar was charged with compound **2-4** (0.32 g, 0.36 mmol) and dry 1,2-dichlorobenzene (20.0 mL), and then BBr_3_ (0.28 mL, 2.88 mmol) was carefully added at room temperature under argon atmosphere. After stirring for 15 minutes, Et_3_N (0.40 mL, 2.88 mmol) was added. The reaction mixture was stirred at 180 °C for 48 h. After cooling to room temperature, 50.0 mL ethanol was added to quench the reaction. The solvent was removed under vacuum and then crude product was purified by column chromatography on silica gel using petroleum ether/dichloromethane (5/1, *v/v*) as eluent to give the corresponding product **BCzBN-3B** as a yellow solid (0.13 g, 39%). ^1^H NMR (500 MHz, CDCl_3_): *δ* (ppm) 9.27 (d, *J* = 1.4 Hz, 2H), 8.64 (d, *J* = 1.5 Hz, 2H), 8.42 (d, *J* = 1.6 Hz, 2H), 7.83 (t, *J* = 7.7 Hz, 4H), 7.72 (t, *J* = 7.6 Hz, 2H), 7.66-7.64 (m, 4H), 7.45 (t, *J* = 8.3 Hz, 1H), 6.80 (d, *J* = 8.2 Hz, 2H), 6.73 (d, *J* = 1.5 Hz, 2H), 1.75 (s, 18H), 1.32 (s, 18H).^13^C NMR (125 MHz, CDCl_3_): *δ* (ppm) 146.40, 146.07, 145.82, 145.78, 145.77, 143.75, 141.40, 140.01, 131.13, 130.16, 129.78, 129.44, 128.86, 128.21, 125.55, 124.98, 122.42, 120.73, 116.06, 35.66, 35.36, 32.51, 32.13. HRMS: (ESI) m/z calcd for C_64_H_58_B_4_N_3_ [M+H]^+^: 915.4935; Found: 915.4946. Elemental analysis: Calc. for C_64_H_57_B_3_N_4_: C, 84.05%; H, 6.28%; N, 6.13%; found: C, 84.13%; H, 6.37%; N, 6.06%.

## 1.3. Single Crystal X-ray Crystallography

X-ray single crystal data was collected on a Bruker D8 Venture diffractometer using CuKα radiation (λ = 1.54178) source. The selected crystal was kept at 150.0 K during data collection. Using Olex2^1^, the structure was solved with the ShelXT^2^ structure solution program using Intrinsic Phasing and refined with the ShelXL^3^ refinement package using Least Squares minimization. Selected crystal data are listed in **Table S1-2**. All crystallographic information in CIF format have been deposited at the Cambridge Crystallographic Data Center (CCDC) under deposition number 2307957 for **DABNA-3B** and 2307956 for **BCzBN-3B** via www.ccdc.cam.ac.uk/data_request/cif, or by emailing [data_request@ccdc.cam.ac.uk](mailto:data_request@ccdc.cam.ac.uk), or by contacting the Cambridge Crystallographic Data Center, 12 Union Road, Cambridge CB2 1EZ, UK; fax: +44 1223 336033.

## 1.4. Quantum Chemical Calculations

All of the simulation calculations were carried out with Gaussian 16 program package. Density functional theory (DFT) calculations on the geometrical and electronic properties of the ground-state were performed based on B3LYP-B3(DJ) density functional method with basis set 6-31G(d,p). Time-dependent DFT (TD-DFT) calculations were also carried out by this method.

The SOCs between S_1_ and T_n_ (n = 1, 2) states were calculated with PySOC by considering that the three T_n_ substrates (m = 1, 0, -1) are degenerate, i.e. $\left\langle S_{1} | \hat{H}_{\mathrm{soc}} | T_{1} \right\rangle=\sqrt{\sum_{m=0,\pm1} \left\langle S_{1} | \hat{H}_{\mathrm{so}} | {T_{1}}^{m} \right\rangle^{2}}$, where The $\hat{H}_{\mathrm{soc}}$ represents the interaction of the SOC.^4^ All SOCs were obtained at the TD-DFT level of theory using the B3LYP functional and the 6-31G(d,p) basis set.

The Huang-Rhys factors (HRF) and reorganization energy for S_1_→S_0_ and S_0_→S_1_ transition were conducted with the DUSHIN module in MOMAP (Molecular Materials Property Prediction Package).

## 1.5. Thermal and Electrochemical Characterization

Thermogravimetric analysis (TGA) was undertaken using TGA-Q50 Instrument (TA Instruments, America) at a heating rate of 10 °C/min from 30 °C to 800 °C under nitrogen flushing. The thermal decomposition temperatures (*T*_d_) were determined by the recorded temperature at 5% weight loss. Differential scanning calorimetry (DSC) was performed at a heating of 10 °C /min from 30 °C to 380 °C using a TA DSC-Q200 (TA Instrument, American) under nitrogen condition, and *T*_g_ was determined from the second heating scan. Cyclic voltammetry (CV) measurements were carried out on a CHI600 electrochemical analyzer (Chenhua, China) at room temperature and a scan speed of 50 mV s^-1^, with a conventional three-electrode system consisting of a glassy carbon working electrode, a platinum wire auxiliary electrode, and an Ag/AgCl standard electrode using as the reference electrode. The supporting electrolyte was 0.1 M tetrabutylammonium hexaﬂuorophosphate (Bu_4_NPF_6_) in anhydrous dichloromethane solution, and ferrocene was added as a calibrant in the whole measurement. The HOMO energy levels of the compounds were calculated according to the formula: *E*_HOMO_ (eV) = - [4.8 + (*E*_1/2(ox/red)_ - *E*_1/2(Fc+/Fc)_)] eV. The LUMO energy levels of the compounds were deduced from the HOMO levels and the UV-Vis absorption onsets of the longer wavelength.

## 1.6. Photophysical Characterization

The UV-vis absorption spectra were obtained on a Shimadzu UV-2600 spectrophotometer (Shimadzu, Japan) at room temperature with a concentration of 1.0 × 10^-5^ M. Phosphorescence spectra were measured on a Hitachi F-7100 fluorescence spectrophotometer at 77 K. The transient photoluminescence (PL) decay curves were obtained by FluoTime 300 (PicoQuant GmbH) with a Picosecond Pulsed UV-LASTER (LASTER375) as the excitation source. The solid-state PL quantum efficiencies (*Φ*_PL_s) were measured on a Hamamatsu UV-NIR absolute PL quantum yield spectrometer (C13534, Hamamatsu Photonics) equipped with an integrating sphere. The integrating sphere was purged with dry argon to maintain an inert atmosphere and all the samples were excited at 370 nm. Nanosecond time-resolved transient absorption spectra and decay kinetics were measured on LFP instrument (LP 980, Edinburgh Instruments LTD). The pump laser beam and the probe beam crossed perpendicularly through the liquid sample in a quartz cuvette (10 mm × 10 mm). A dynamic decay curve was recorded with a digital phosphor oscilloscope (TDS 3012C, Tektronix Inc.).

## 1.7. Analysis of Rate Constants

The estimation of rate constants pertaining to radiative decay (*k*_r_) and nonradiative decay (*k*_nr_) transitioning from S_1_ to S_0_, as well as the rate constants associated with intersystem crossing (*k*_ISC_) and reverse intersystem crossing (*k*_RISC_), can be accomplished by employing the subsequent equations.^5-7^

$k_{r}= \Phi_{p}k_{p}+\Phi_{d}k_{d}\approx\Phi_{p}k_{p}$…………………………………………………..Eq.(1)

$k_{\mathrm{nr}}=\frac{1-\Phi_{\mathrm{PL}}}{\Phi_{\mathrm{PL}}}k_{r}$…………………………………………………………………Eq.(2)

$k_{\mathrm{ISC}}=k_{p}-k_{r}-k_{\mathrm{nr}}$………………………………………………………….Eq.(3)

$k_{\mathrm{RISC}}= \left( k_{p}k_{d}\Phi_{d} \right)/\left( k_{\mathrm{ISC}}\Phi_{p} \right)$…………………………………………………Eq.(4)

Where *k*_p_ and *k*_d_ represent the decay rate constants for prompt and delayed fluorescence, respectively, which are in reciprocal relationship with the prompt and delayed lifetime (*τ*_p_ and *τ*_d_) experimentally determined from transient PL characteristics. *Φ*_p_ and *Φ*_d_ indicate prompt and delayed fluorescence quantum yields and can be distinguished from the total *Φ*_PL_ by comparing the integrated intensities of prompt (*r*_p_) and delayed components (*r*_d_) in the transient PL spectra. *r*_p_ and *r*_d_ were determined using *τ*_p_ and *τ*_d_ and fitting parameter (*A*_p_, *A*_d_) as follows.

$I\left( t \right){=A}_{p}e^{-\frac{1}{\tau_{p}}}{+A}_{d}e^{-\frac{1}{\tau_{d}}}$…………………………………………………….Eq.(5)

$r_{p}=A_{p}\tau_{p}/(A_{p}\tau_{p}+A_{d}\tau_{d})$ …………………………………………………Eq.(6)

$r_{d}=A_{d}\tau_{d}/(A_{p}\tau_{p}+A_{d}\tau_{d})$ …………………………………………………Eq.(7)

## 1.8. Device Fabrication and Measurement

The ITO coated glass substrates with a sheet resistance of 15 Ω square^-1^ were consecutively ultrasonicated with acetone/ethanol and dried with nitrogen gas flow, followed by 20 min ultraviolet light-ozone (UVO) treatment in a UV-ozone surface processor (PL16 series, Sen Lights Corporation). Then the sample was transferred to the deposition system. Both 8-hydroxyquinolinolato-lithium (Liq) as electron injection layer and aluminum (Al) as cathode layer were deposited by thermal evaporation at 5 × 10^-5^ Pa. The organic layers were deposited at the rates of 0.2-3 Å/s. After the organic film deposition, Liq and Al layer were deposited with rates of 0.1 and 3 Å/s, respectively. The emitting area of the device is about 0.09 cm^2^. The current density-voltage-luminance (*J*-*V*-*L*), EQE*-L* curves and electroluminescence spectra were measured using a Keithley 2400 source meter and an absolute EQE measurement system (C9920-12, Hamamatsu Photonics, Japan).

## 1.9. Determination of Emitting Dipole Orientation

The emitting dipole orientation was determined by angle-resolved and polarization-resolved PL measurements in a Hamamatsu’s established molecular orientation measurement system (C14234-11, Hamamatsu Photonics). The sample consisted of a fused silica substrate with 15-nm-thick DBFPO film doped with 1 wt% emitter. The sample was attached to a fused silica half-cylinder prism by index matching liquid. The excitation of the samples was performed with the 360-nm line of the continuous-wave laser with a fixed excitation angle of 45^o^. The angle-dependent *p*-polarized emission intensity at the peak wavelength of the PL spectrum of the emitting layer was detected. The emitting dipole orientation (Θ_//_) was then determined by least-square fitting of the measured angle-dependent *p*-polarized emission intensity with calculated results.

# 2. Figures and Tables


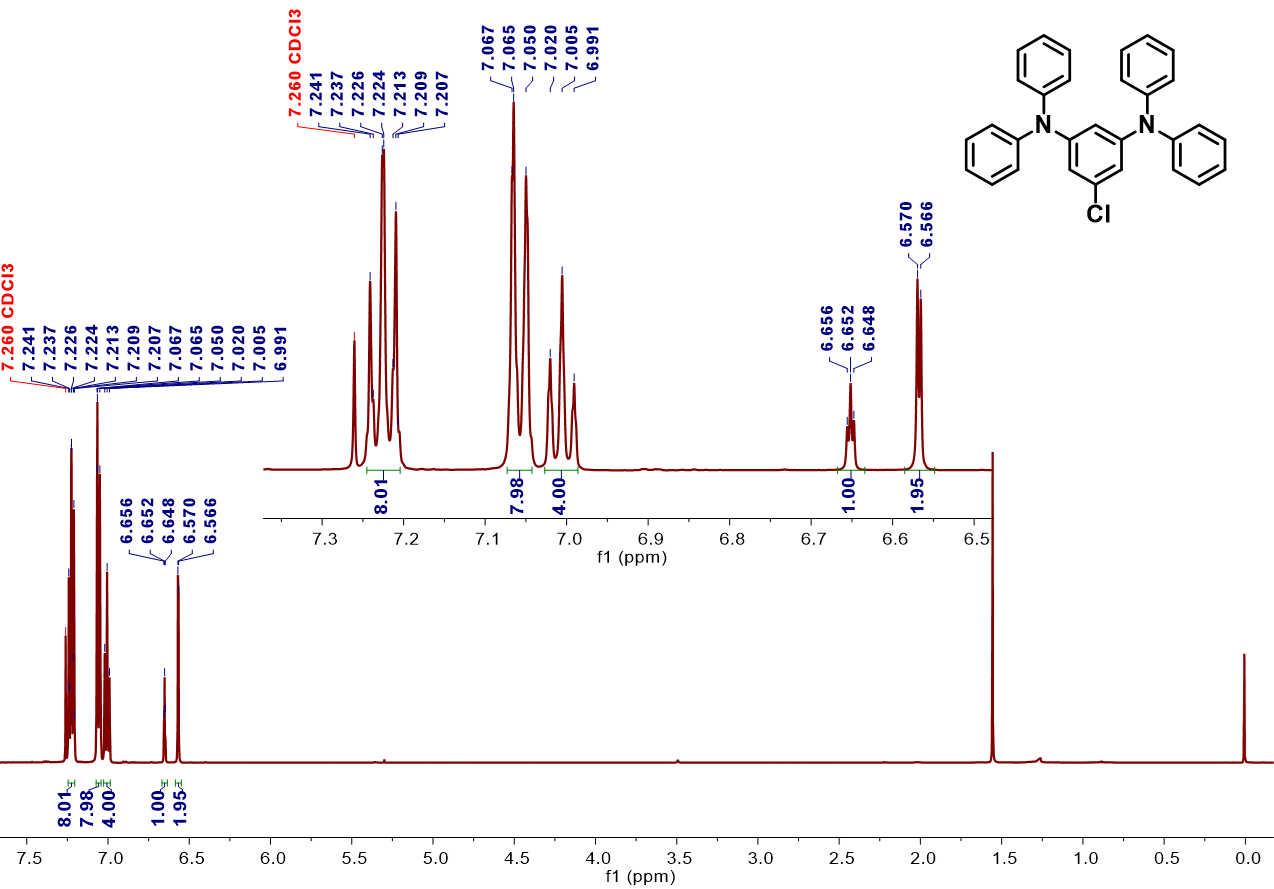


**Figure S1**. ^1^H NMR spectrum of **1-1** in CDCl_3_ (500 MHz, 25 °C).


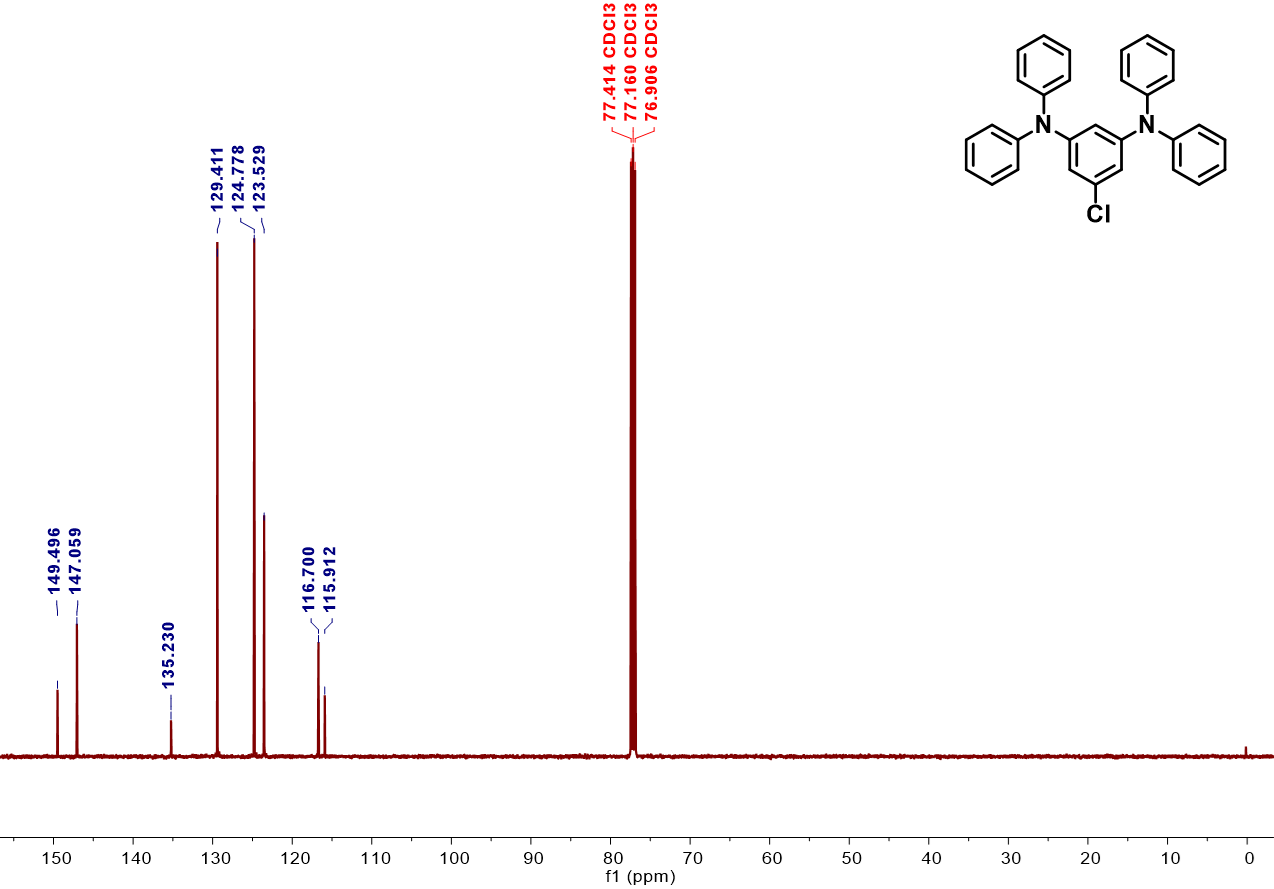


**Figure S2**. ^13^C NMR spectrum of **1-1** in CDCl_3_ (125 MHz, 25 °C).


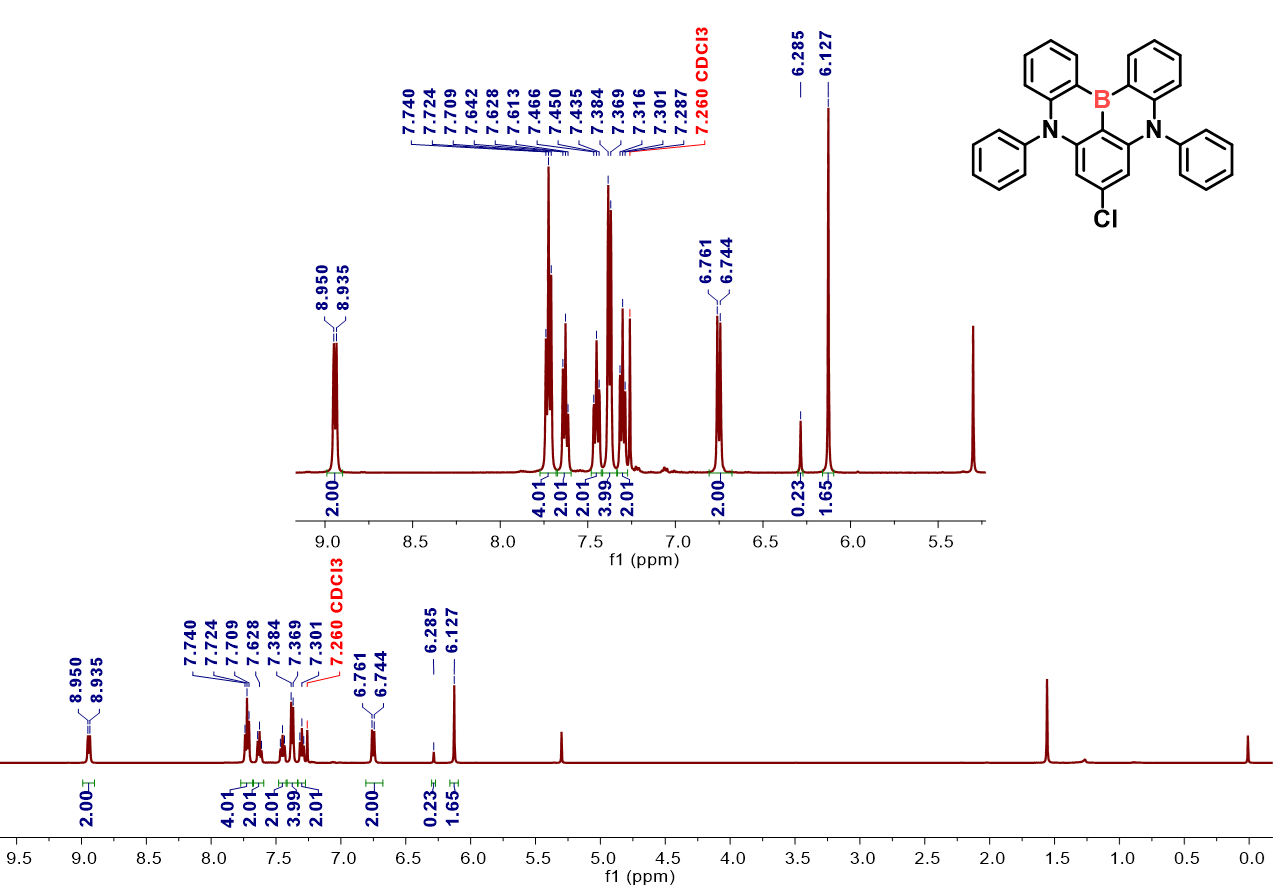


**Figure S3**. ^1^H NMR spectrum of **1-2** in CDCl_3_ (500 MHz, 25 °C).


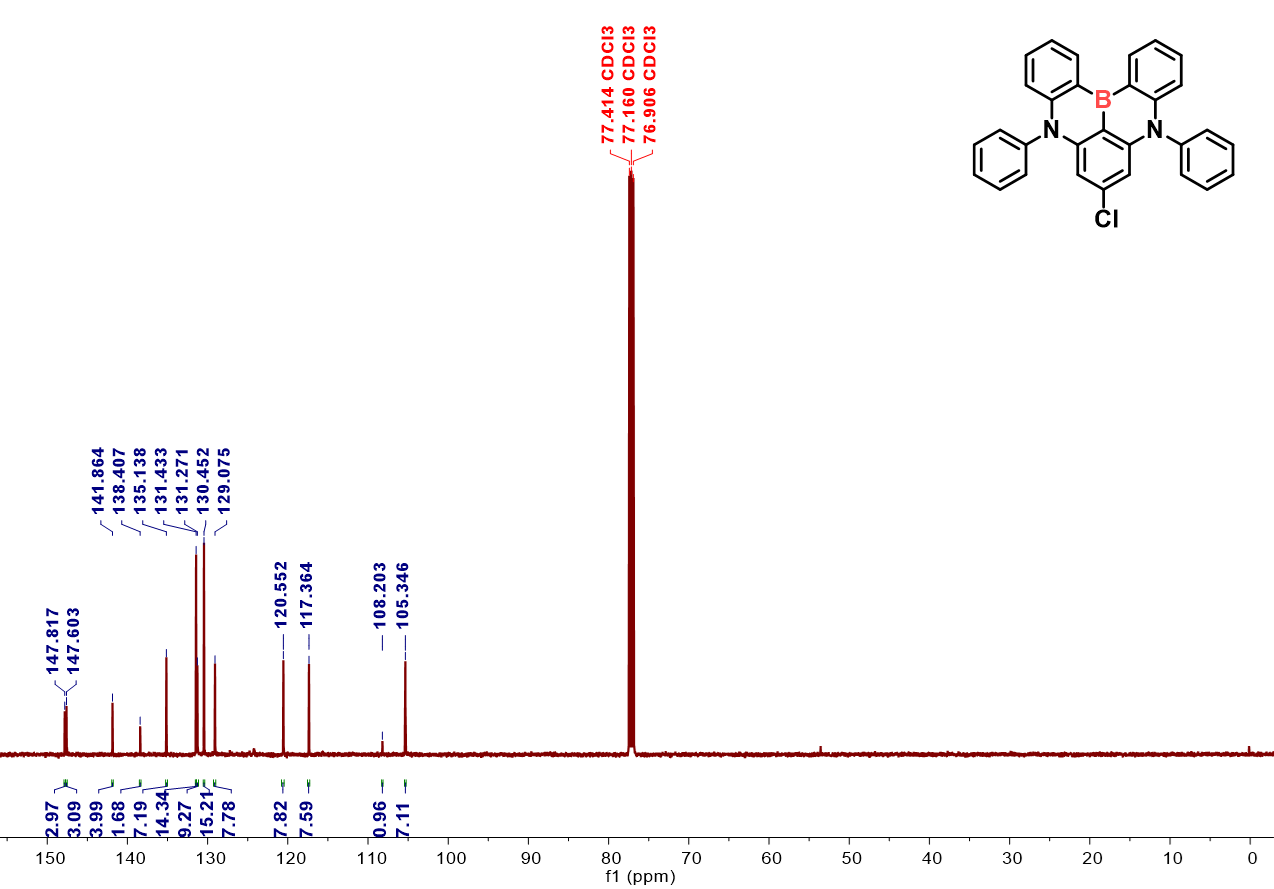


**Figure S4**. ^13^C NMR spectrum of **1-2** in CDCl_3_ (125 MHz, 25 °C).

**
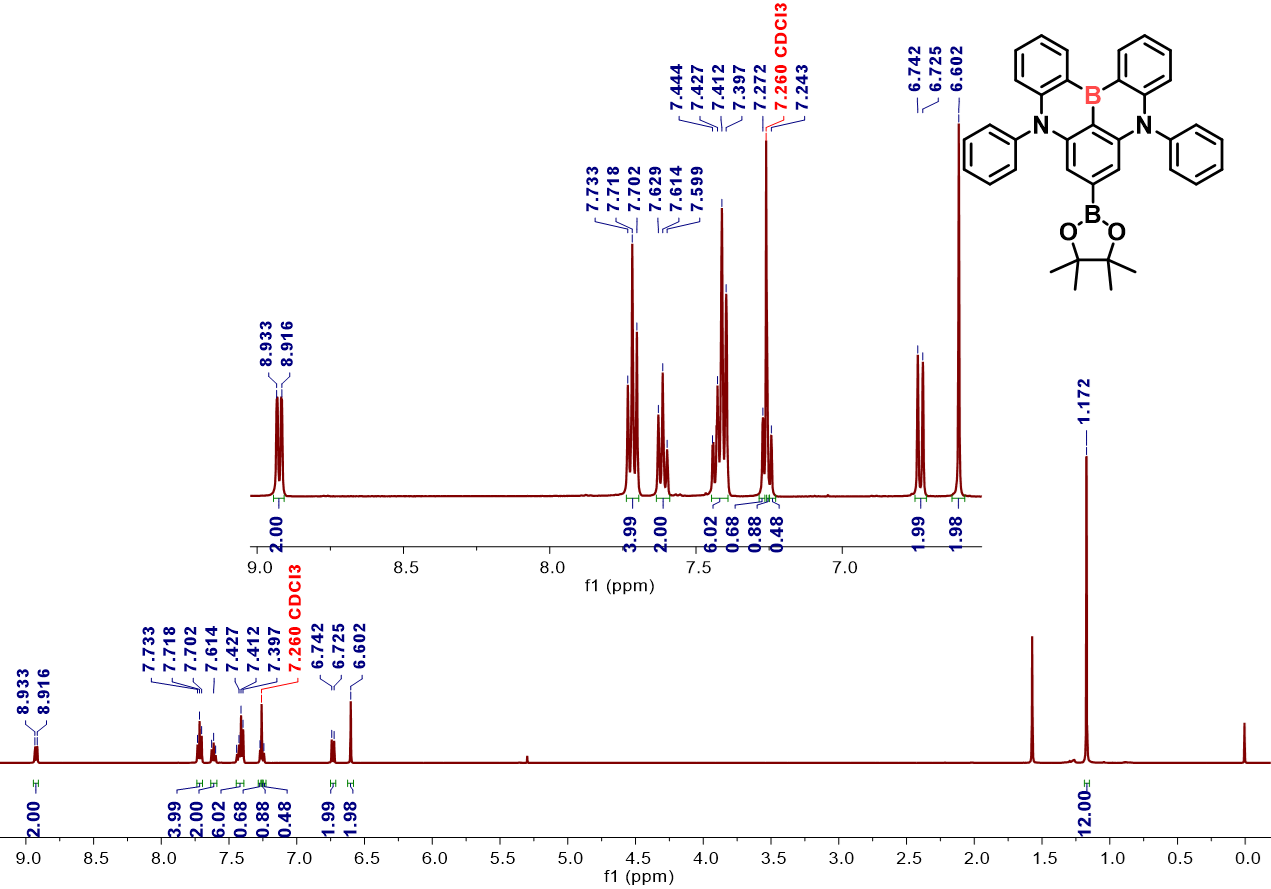
**

**Figure S5**. ^1^H NMR spectrum of **1-3** in CDCl_3_ (500 MHz, 25 °C).


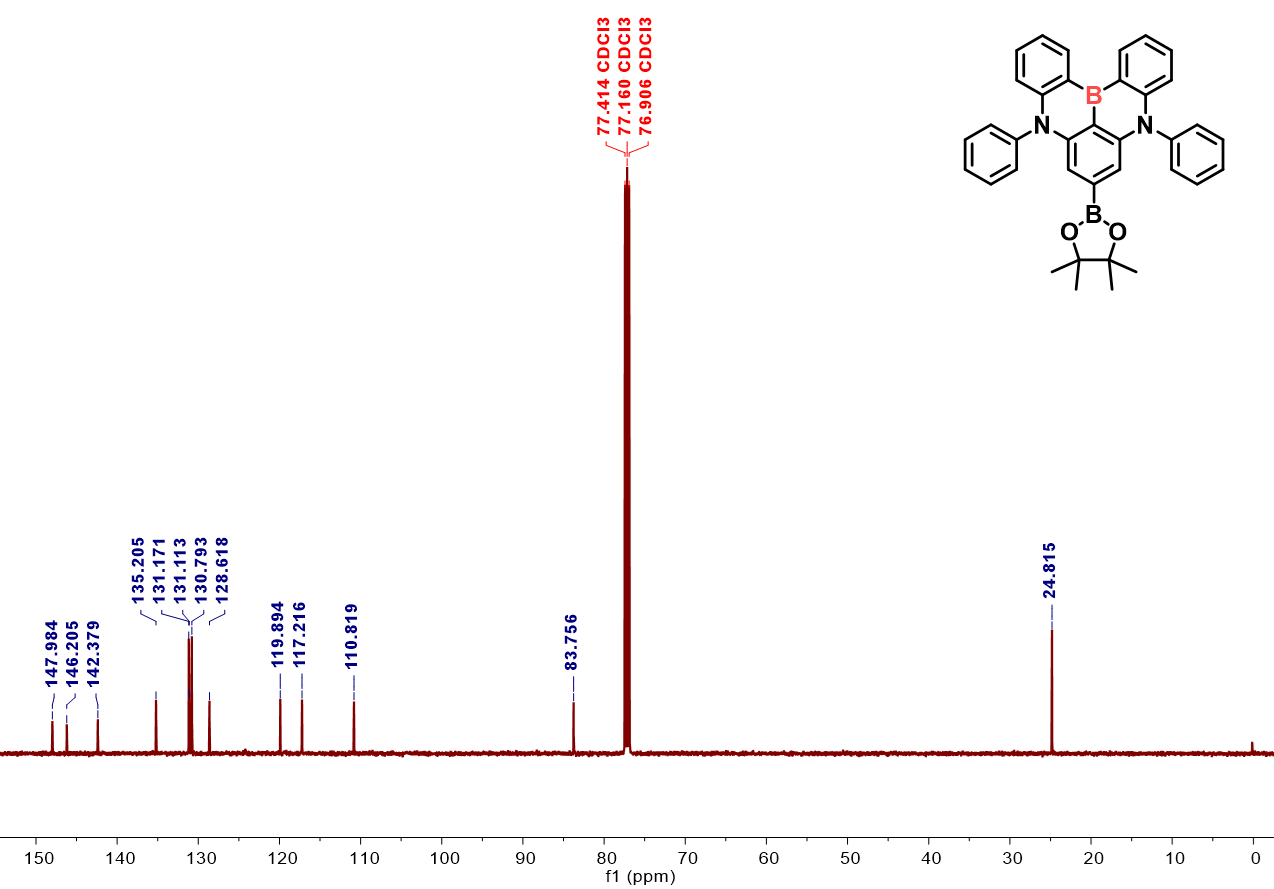


**Figure S6**. ^13^C NMR spectrum of **1-3** in CDCl_3_ (125 MHz, 25 °C).

**
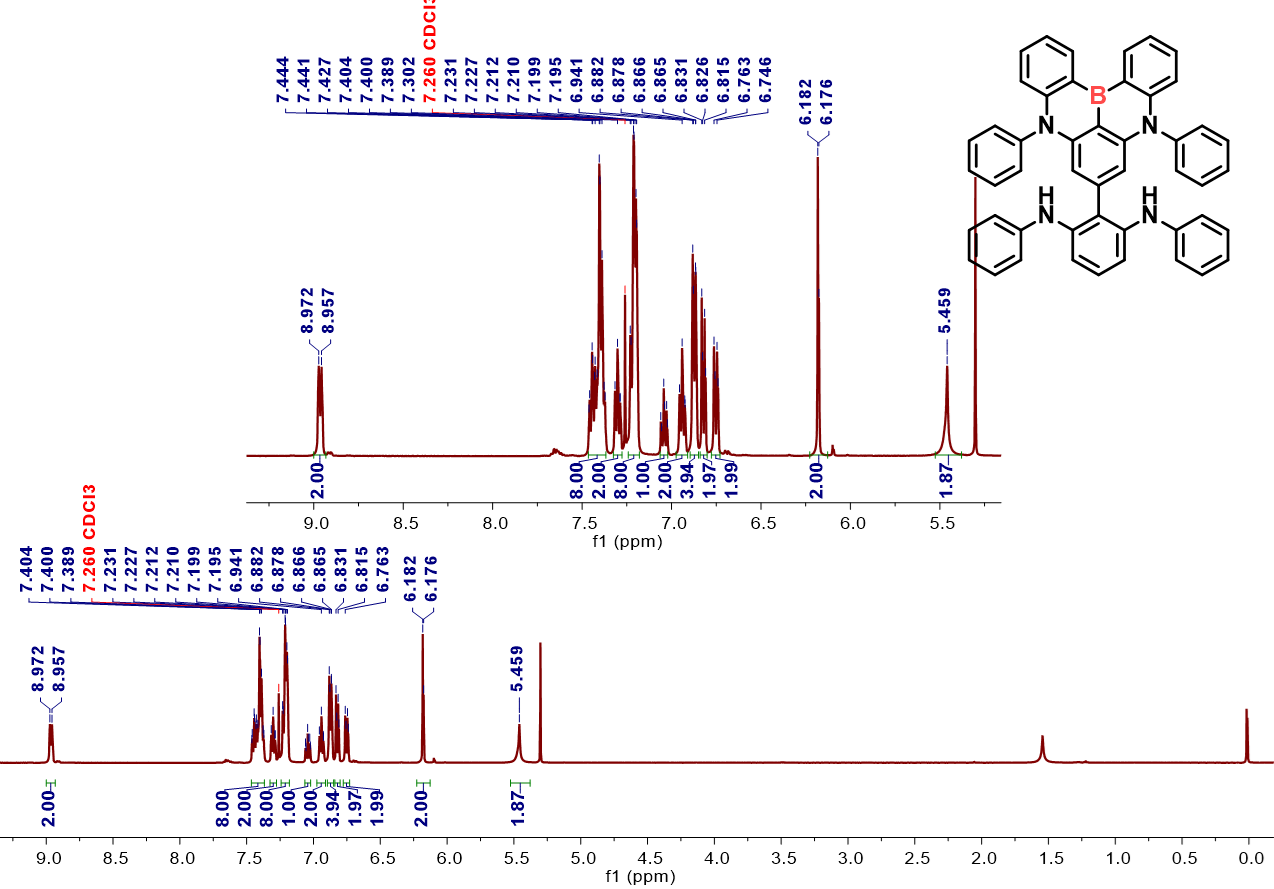
**

**Figure S7**. ^1^H NMR spectrum of **1-4** in CDCl_3_ (500 MHz, 25 °C).


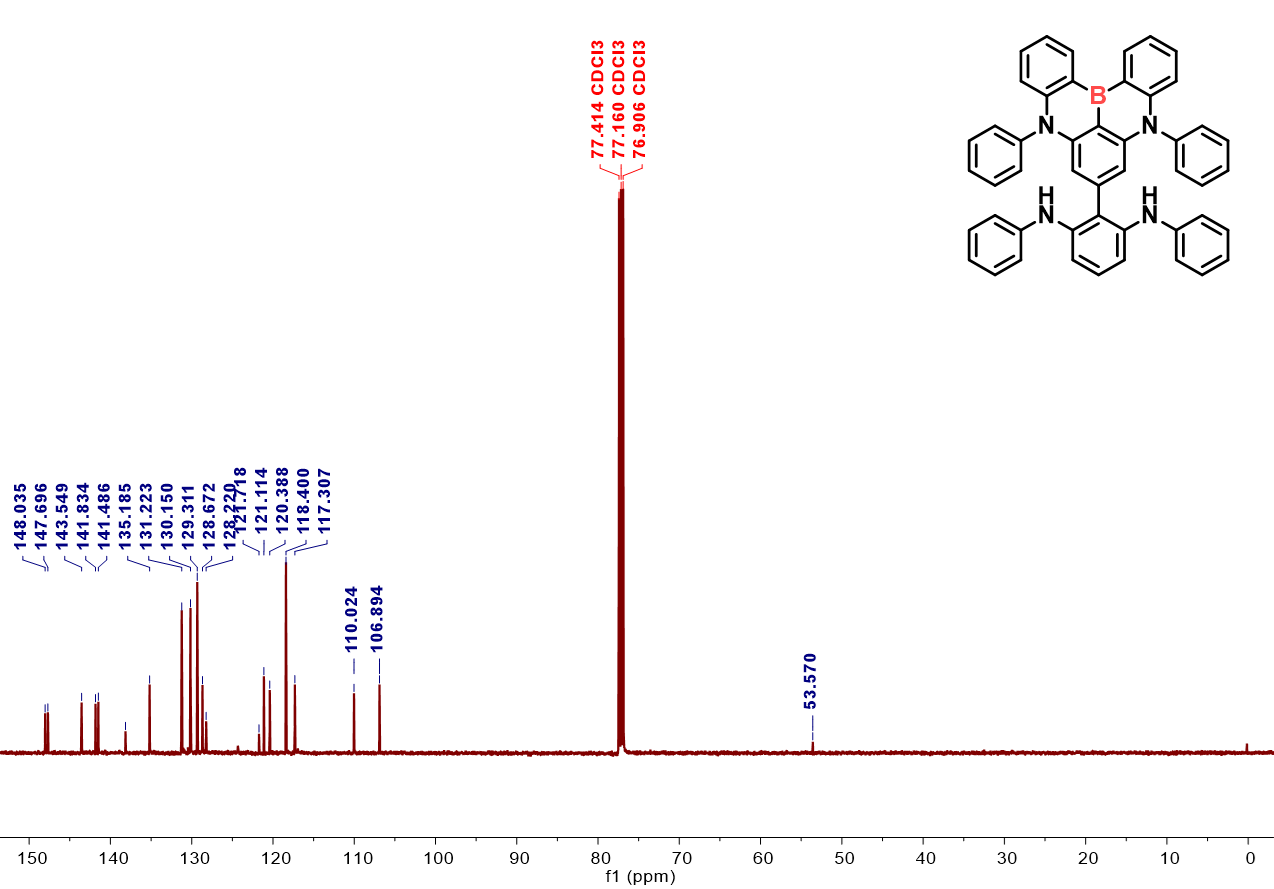


**Figure S8**. ^13^C NMR spectrum of **1-4** in CDCl_3_ (125 MHz, 25 °C).


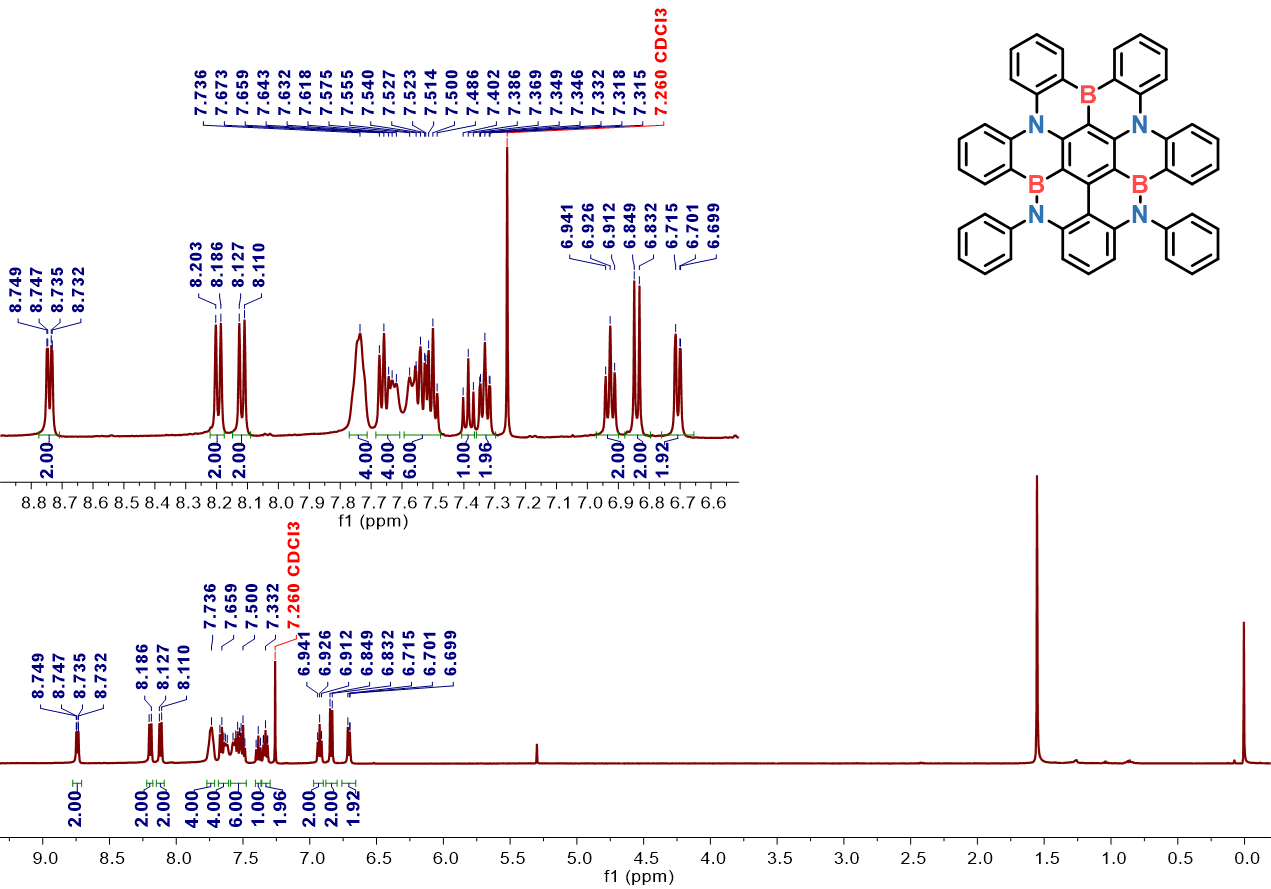


**Figure S9**. ^1^H NMR spectrum of **DABNA-3B** in CDCl_3_ (500 MHz, 25 °C).


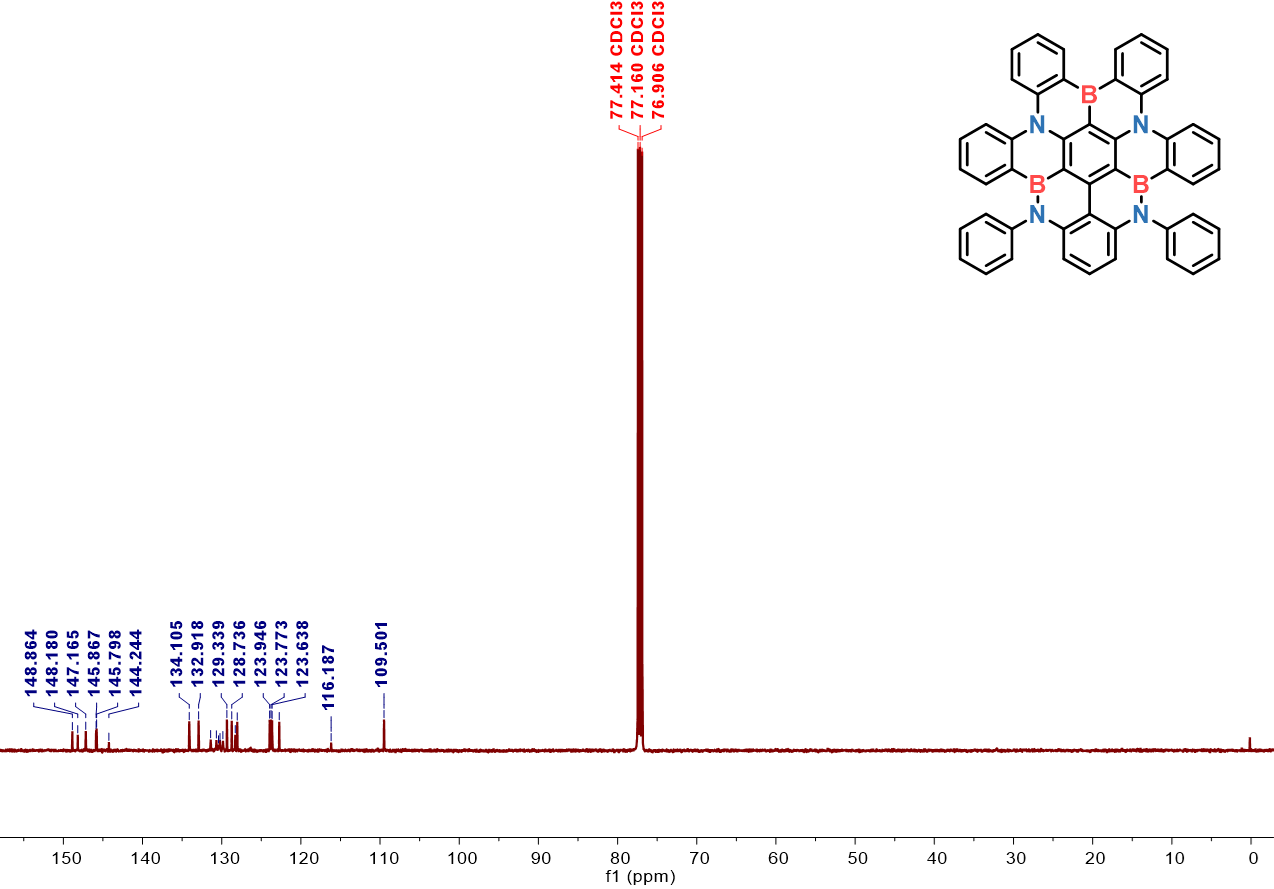


**Figure S10**. ^13^C NMR spectrum of **DABNA-3B** in CDCl_3_ (125 MHz, 25 °C).


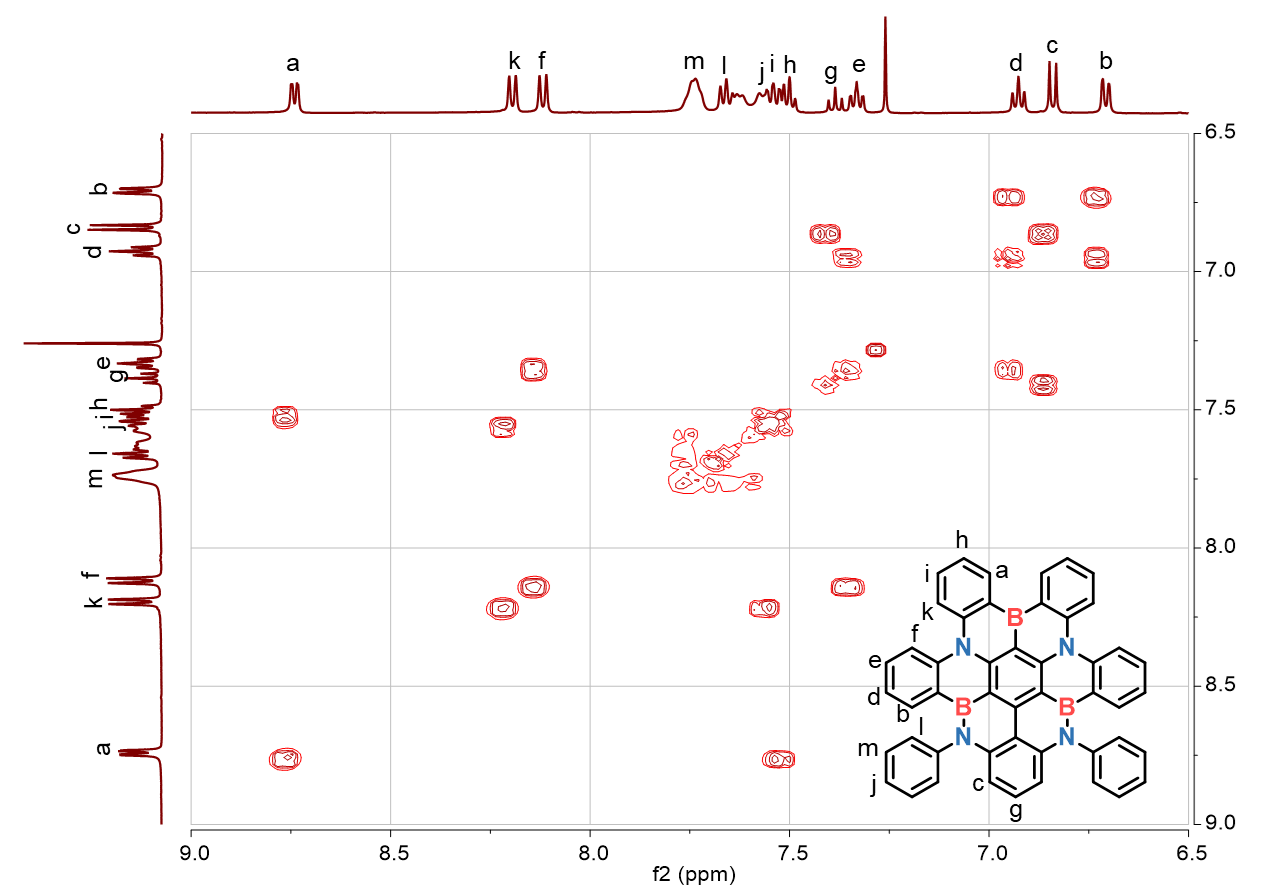


**Figure S11**. COSY spectrum of **DABNA-3B** in CDCl_3_ (500 MHz, 25 °C).


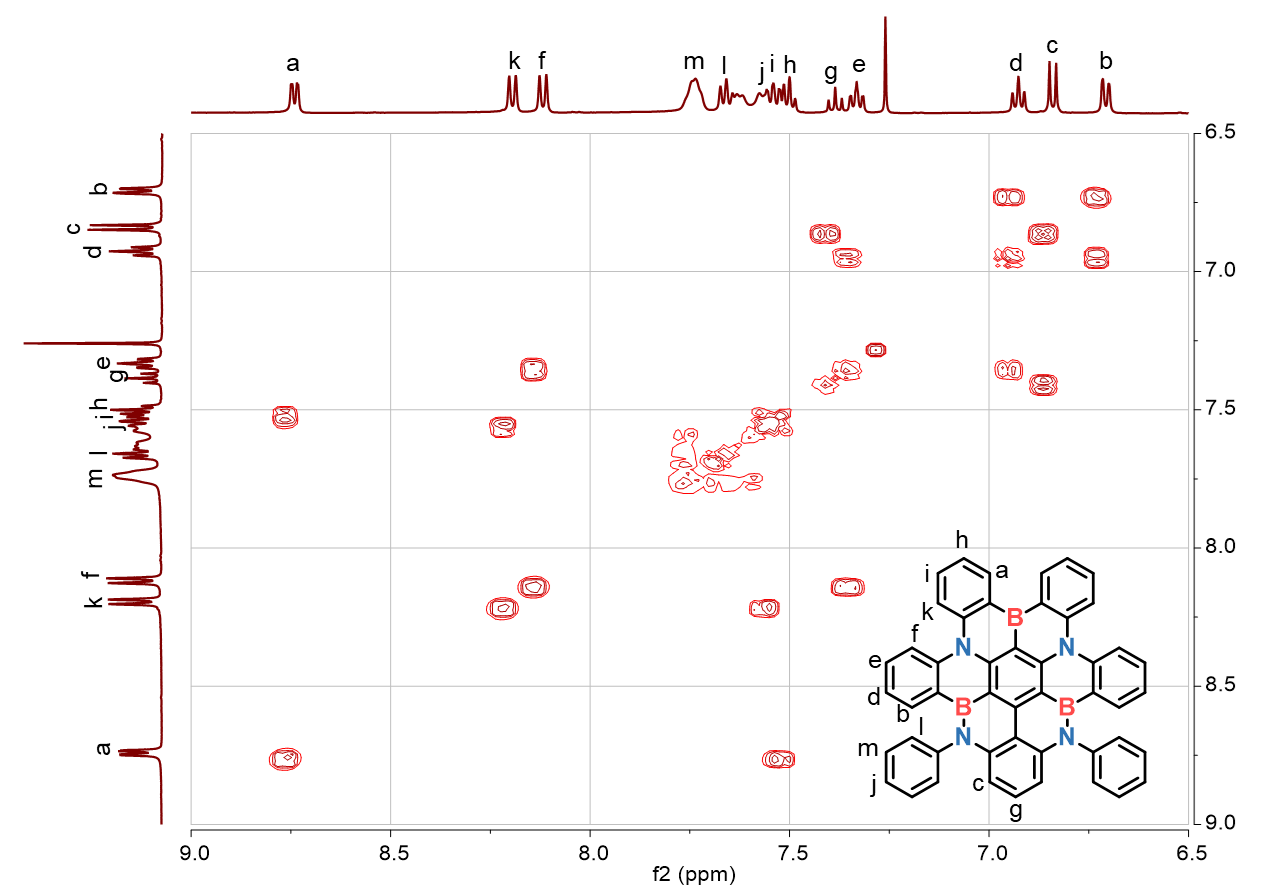


**Figure S12**. ROESY spectrum of **DABNA-3B** in CDCl_3_ (500 MHz, 25 °C).

**
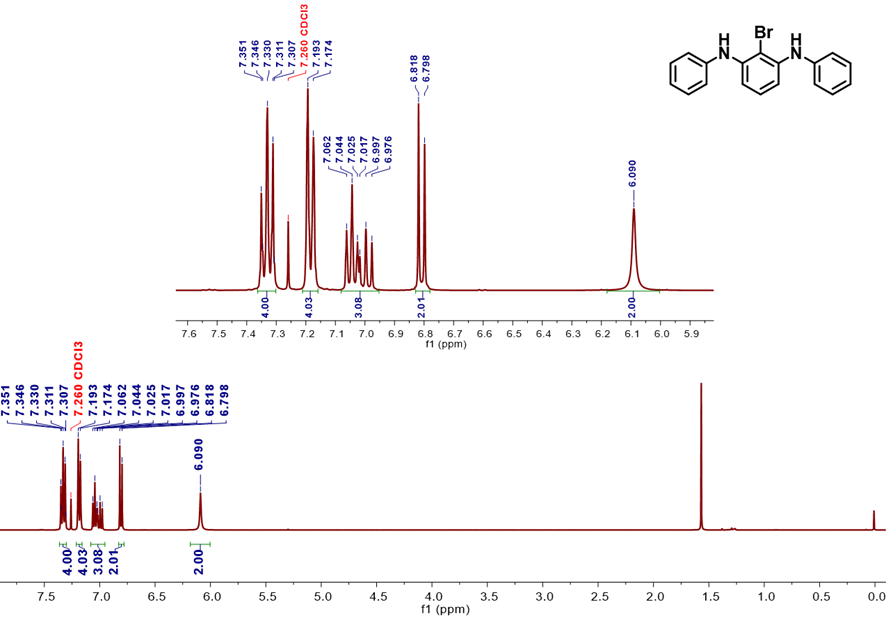
**

**Figure S13**. ^1^H NMR spectrum of **3** in CDCl_3_ (500 MHz, 25 °C).


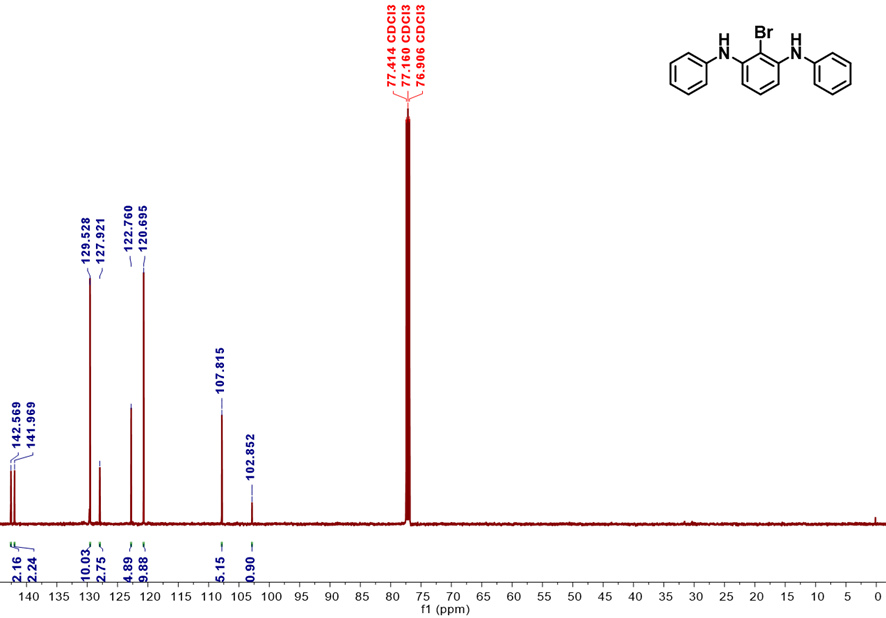


**Figure S14**. ^13^C NMR spectrum of **3** in CDCl_3_ (125 MHz, 25 °C).


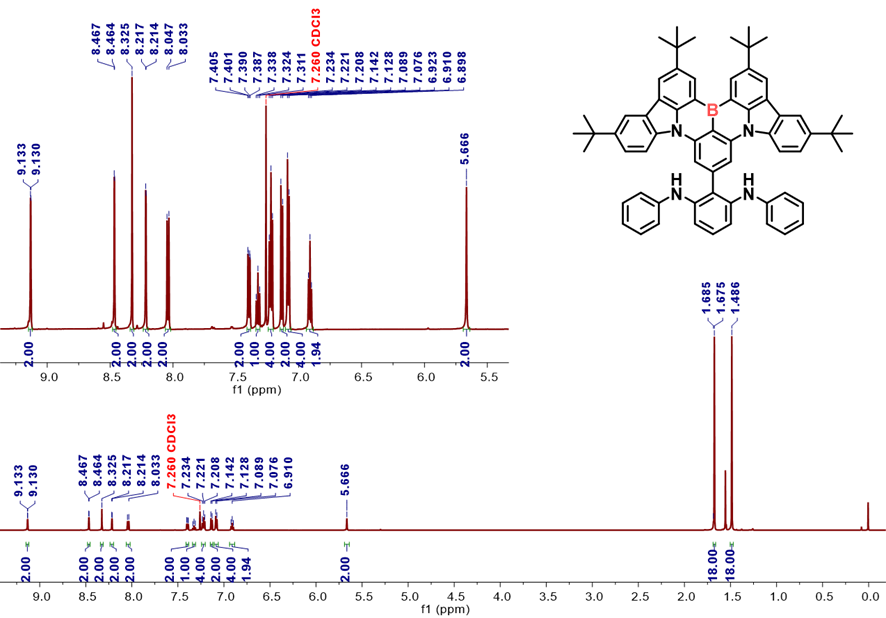


**Figure S15**. ^1^H NMR spectrum of **2-4** in CDCl_3_ (600 MHz, 25 °C).


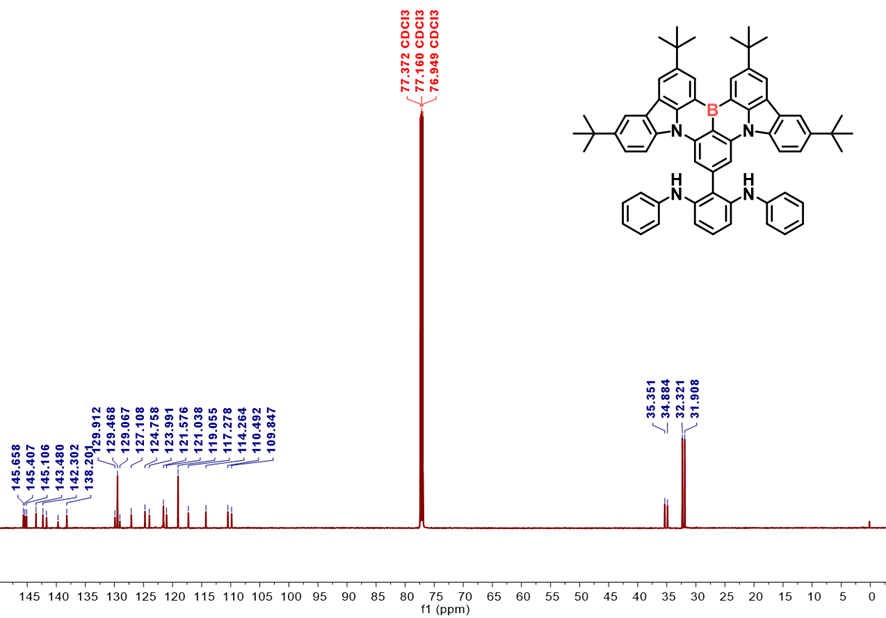


**Figure S16**. ^13^C NMR spectrum of **2-4** in CDCl_3_ (125 MHz, 25 °C).


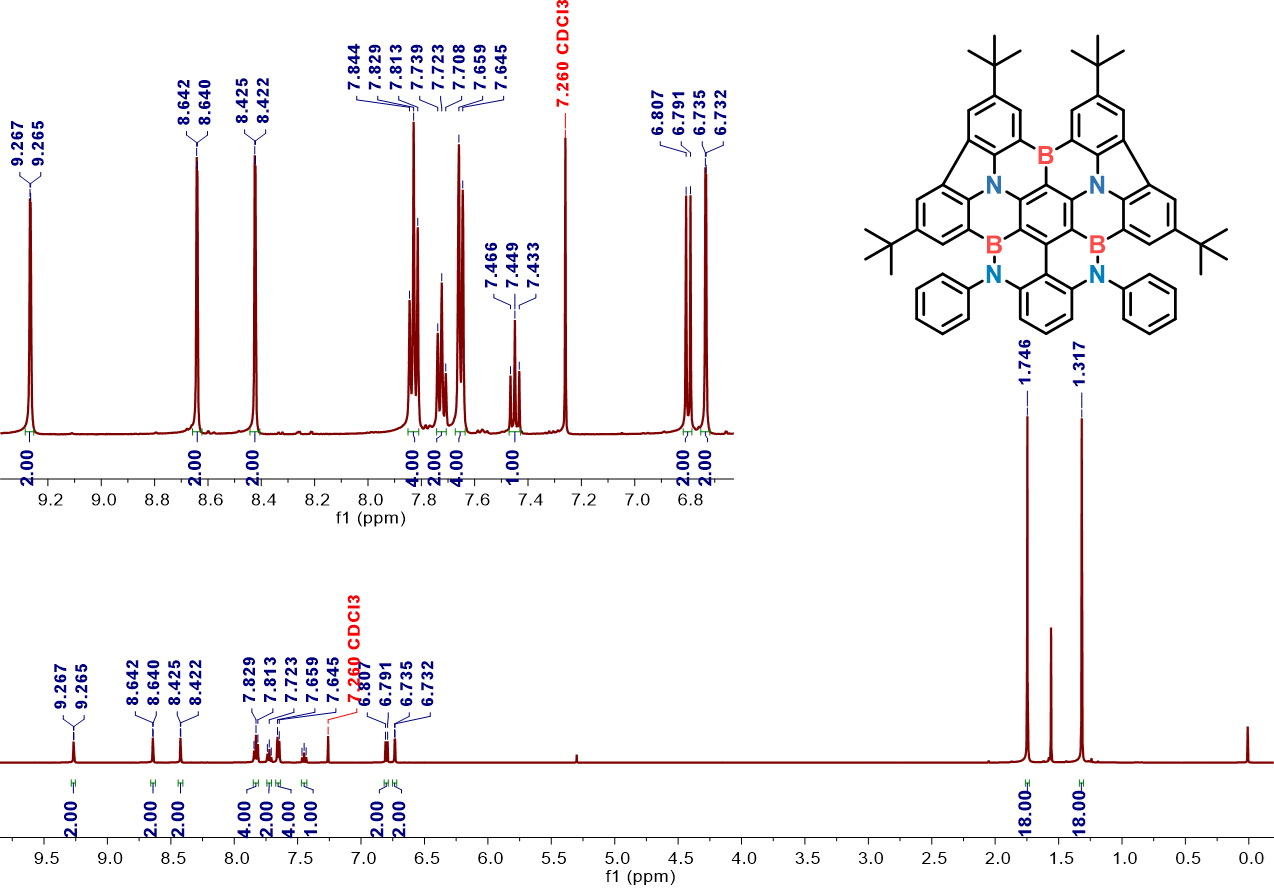


**Figure S17**. ^1^H NMR spectrum of **BCzBN-3B** in CDCl_3_ (500 MHz, 25 °C).


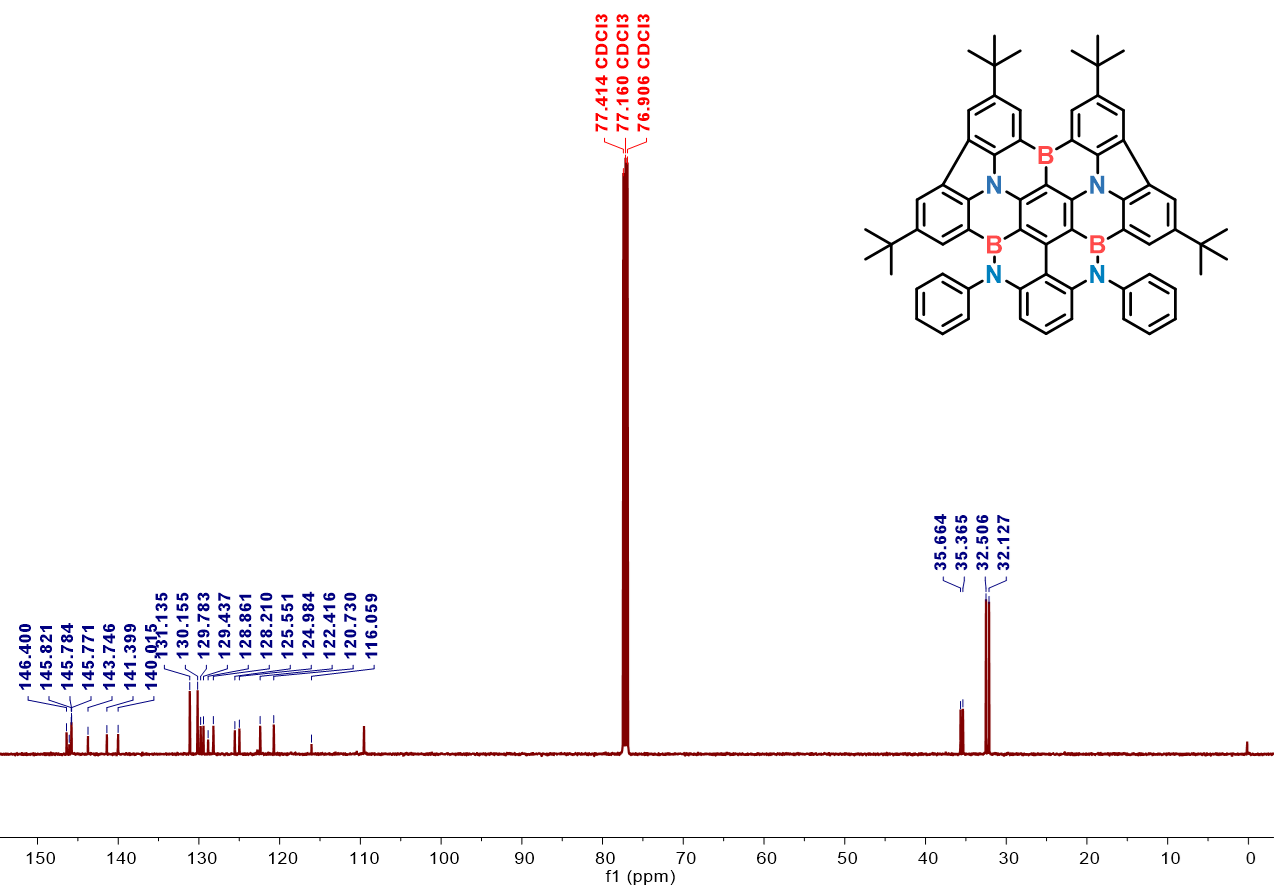


**Figure S18**. ^13^C NMR spectrum of **BCzBN-3B** in CDCl_3_ (125 MHz, 25 °C).


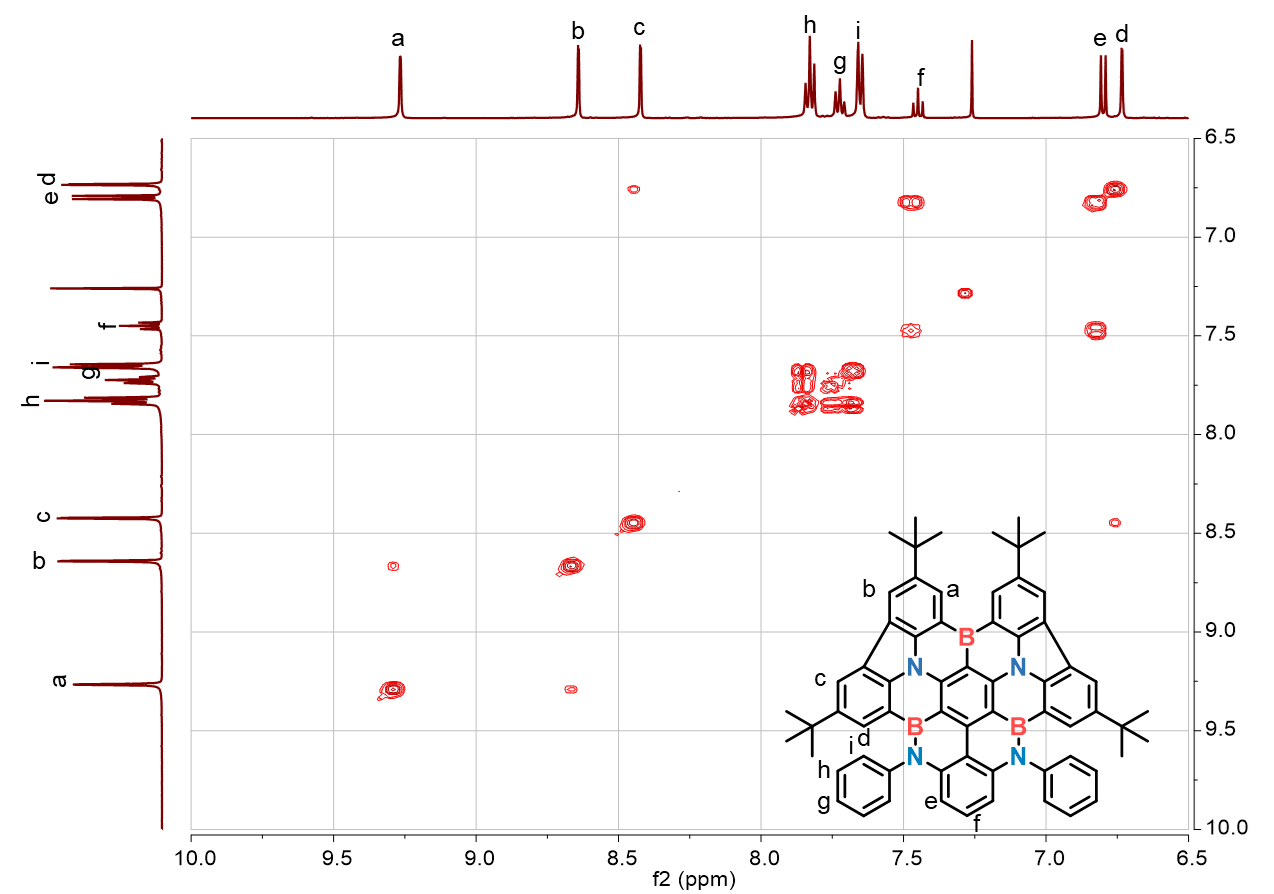


**Figure S19**. COSY spectrum of **BCzBN-3B** in CDCl_3_ (500 MHz, 25 °C).


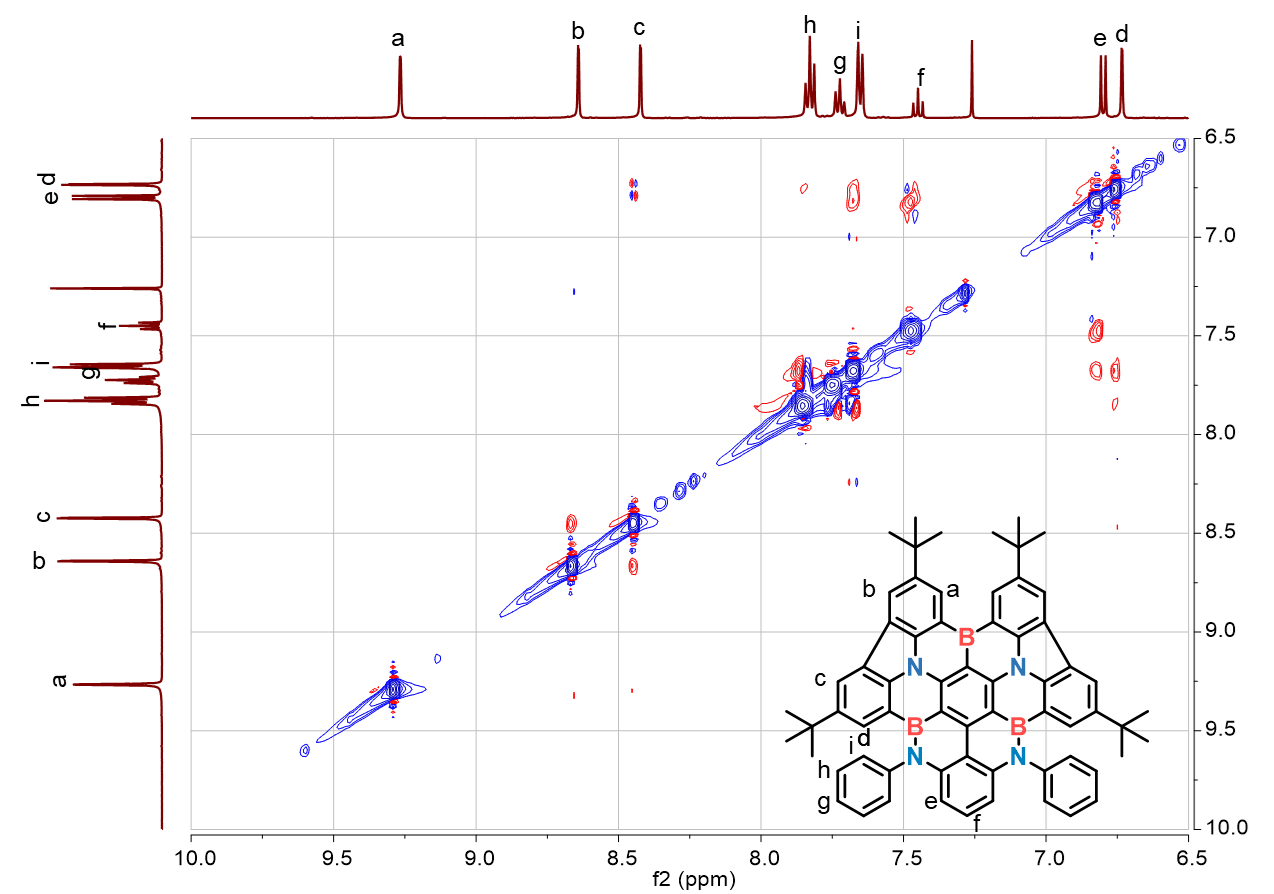


**Figure S20**. ROESY spectrum of **BCzBN-3B** in CDCl_3_ (500 MHz, 25 °C).


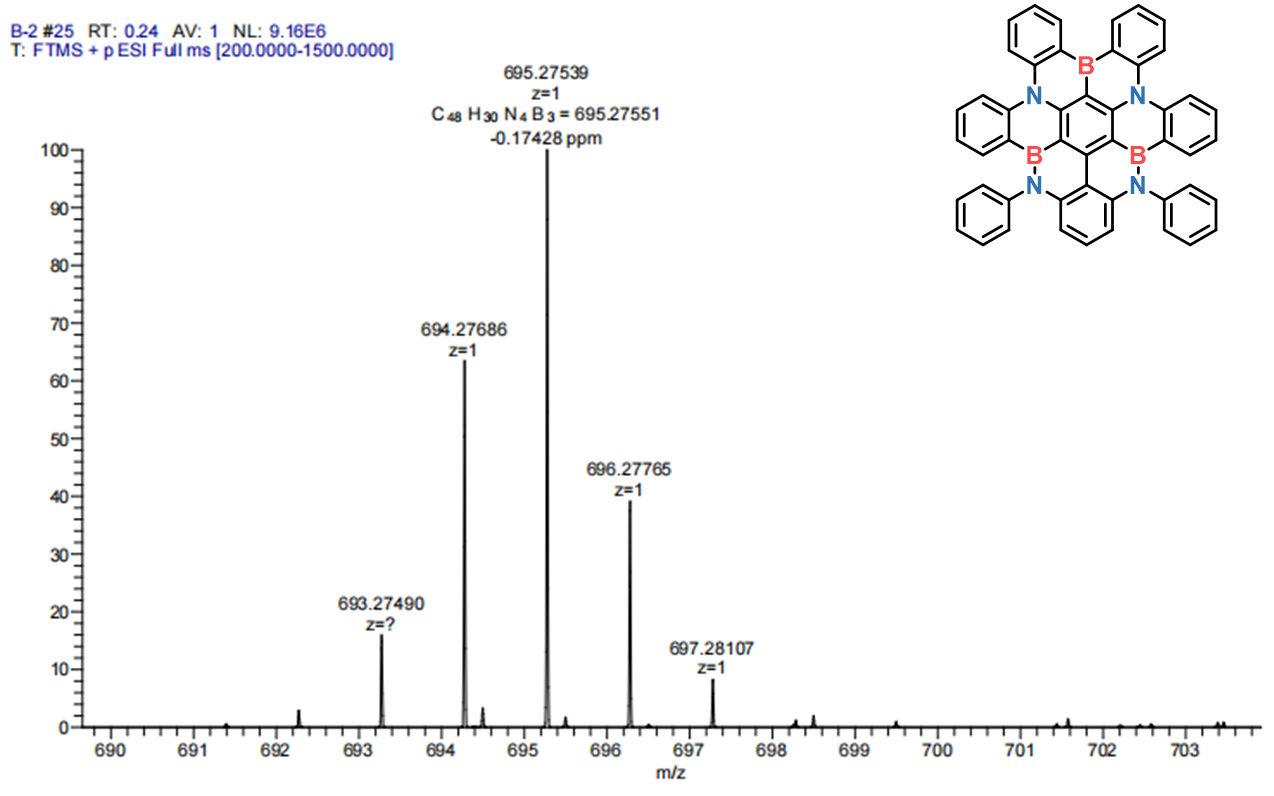


**Figure S21**. HRMS spectrum of **DABNA-3B**.


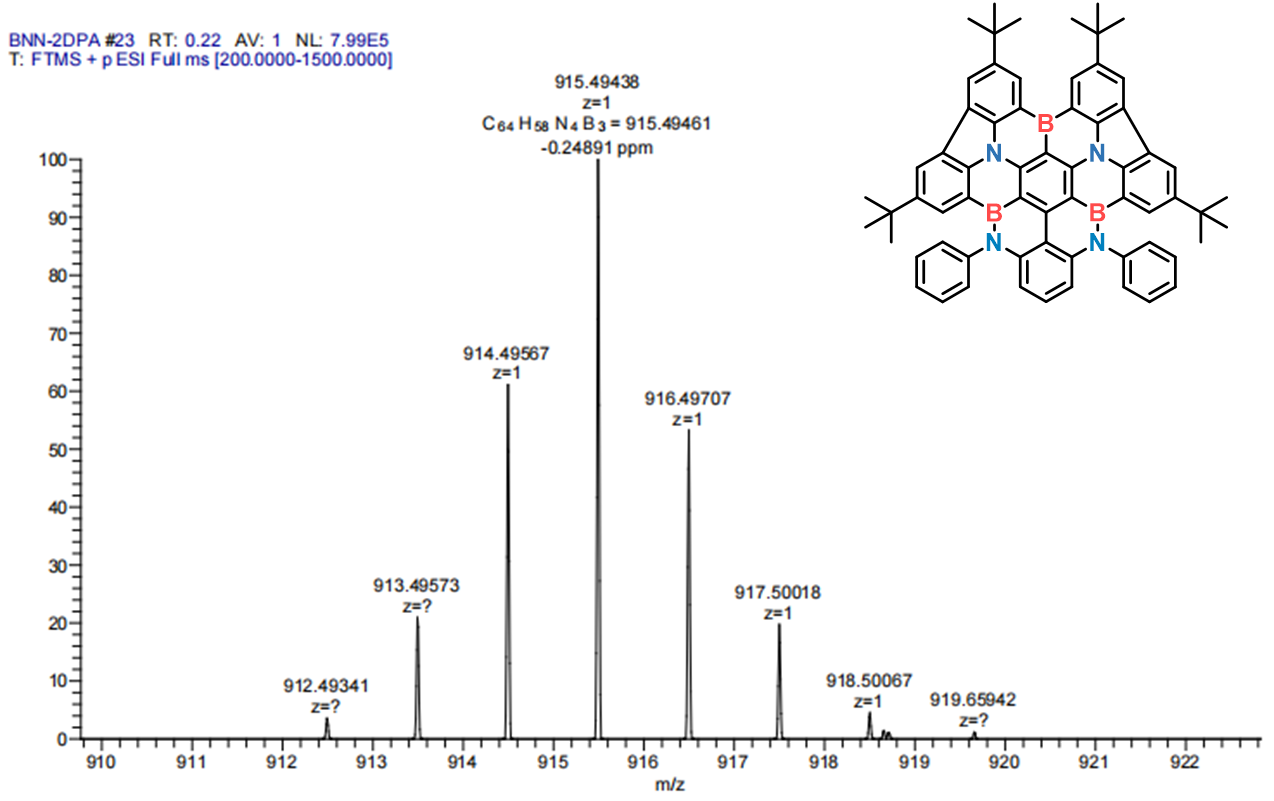


**Figure S22**. HRMS spectrum of **BCzBN-3B**.

**Figure S23**. Thermal gravimetric analysis (TGA) curves of (A) **DABNA-3B** and (B) **BCzBN-3B** at a heating rate of 10 °C min^-1^, inset: differential scanning calorimetry (DSC) traces at a heating rate of 10 °C min^-1^. The oxidation curves obtained from the cyclic voltammetry (CV) measurement for (C) **DABNA-3B** and (D) **BCzBN-3B**.

**Figure S24**. Natural transition orbital (NTO) distributions of S_1_, T_1_ and T_2_ states (hole/particle wave functions are shown in green/orange color).

**Figure S25**.The reorganization energies versus frequencies for S_1_→S_0_ transition of the emitters.

**Figure S26**. The photoluminescence spectra measured in different polar solvents (1 × 10^−5^ M, 300 K) of (A) **DABNA**, (B) **DABNA-3B**, (C) **BCzBN** and (D) **BCzBN-3B**.

**Figure S27**. Photoluminescence spectra of (A) **DABNA**, (B) **DABNA-3B**, (C) **BCzBN** and (D) **BCzBN-3B** in mCBP film (2 wt%) at 300 K.

**Figure S28**. Transient PL spectra of (A) **DABNA-3B** and (B) **BCzBN-3B** in mCBP film (2 wt%) at variable temperatures.

**Figure S29**. Photoluminescence spectra of (A) 2 wt% **DABNA-3B**: SiTRZCz2 doped film and (B) 2 wt% **BCzBN-3B**: DMIC-TRZ doped film at 300 K.

**
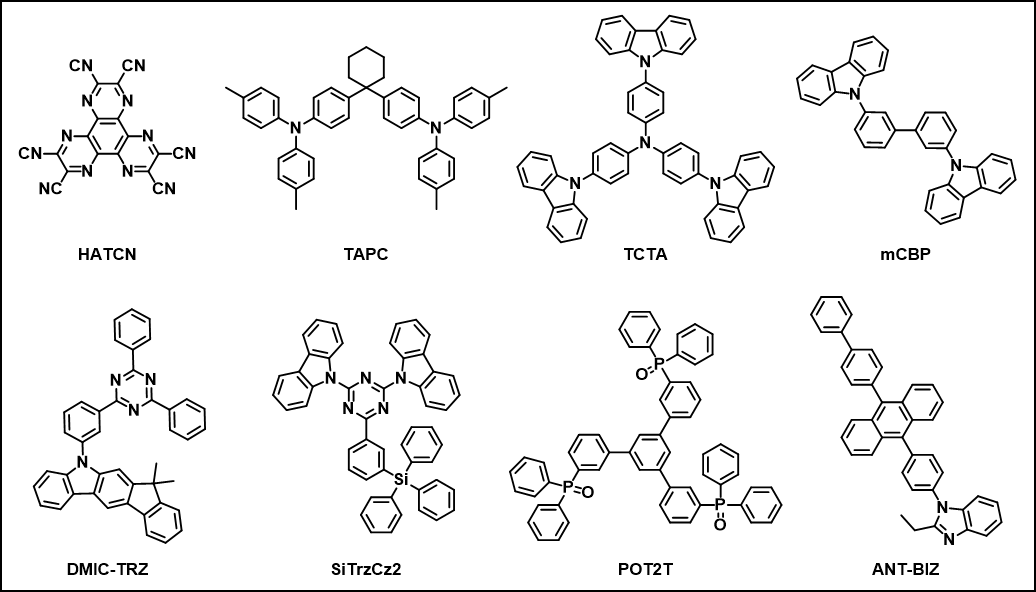
**

**Figure S30**. Chemical structures of materials utilized for device fabrication.

**Figure S31**. EL spectra of the devices based on (A) **DABNA**, (B) **DABNA-3B**, (C) **BCzBN** and (D) **BCzBN-3B** at different voltages.

**Figure S32**. The length, width, and height of (A) **DABNA**, (B) **DABNA-3B**, (C) **BCzBN** and (D) **BCzBN-3B** at the optimized S_0_ geometry.

**Figure S33**. Efficiency roll-off fitting results of EQE-current density curves of (A) **DABNA**, (B) **DABNA-3B**, (C) **BCzBN** and (D) **BCzBN-3B**.

**Table S1.** Crystal data for **DABNA-3B**.

| Identification code | **DABNA-3B** |
| --- | --- |
| Empirical formula | C_55_H_37_B_3_N_4_ |
| Formula weight | 786.31 |
| Temperature/K | 150.00 |
| Crystal system | triclinic |
| Space group | P-1 |
| a/Å | 13.9082(10) |
| b/Å | 14.1089(10) |
| c/Å | 22.5757(16) |
| α/° | 81.822(4) |
| β/° | 84.553(4) |
| γ/° | 65.787(4) |
| Volume/Å^3^ | 3995.9(5) |
| Z | 4 |
| ρcalcg/cm^3^ | 1.307 |
| μ/mm^‑1^ | 0.579 |
| F(000) | 1640.0 |
| Crystal size/mm^3^ | 0.3 × 0.05 × 0.05 |
| Radiation | CuKα (λ = 1.54178) |
| 2Θ range for data collection/° | 3.958 to 138.126 |
| Index ranges | -16 ≤ h ≤ 16, -16 ≤ k ≤ 17, 0 ≤ l ≤ 27 |
| Reflections collected | 14333 |
| Independent reflections | 14333 [R_int_ = ?, R_sigma_ = 0.0499] |
| Data/restraints/parameters | 14333/254/1202 |
| Goodness-of-fit on F^2^ | 1.063 |

**Table S2.** Crystal data for **BCzBN-3B**.

| Identification code | **BCzBN-3B** |
| --- | --- |
| Empirical formula | C_64_H_57_B_3_N_4_ |
| Formula weight | 914.56 |
| Temperature/K | 150.15 |
| Crystal system | triclinic |
| Space group | P-1 |
| a/Å | 5.8401(4) |
| b/Å | 13.0042(8) |
| c/Å | 32.651(2) |
| α/° | 83.571(3) |
| β/° | 87.606(3) |
| γ/° | 78.338(4) |
| Volume/Å^3^ | 2412.8(3) |
| Z | 2 |
| ρ_calc_g/cm^3^ | 1.259 |
| μ/mm^‑1^ | 0.548 |
| F(000) | 968.0 |
| Crystal size/mm^3^ | 0.15 × 0.05 × 0.05 |
| Radiation | CuKα (λ = 1.54178) |
| 2Θ range for data collection/° | 5.448 to 137.738 |
| Index ranges | -7 ≤ h ≤ 7, -14 ≤ k ≤ 15, -39 ≤ l ≤ 39 |
| Reflections collected | 50226 |
| Independent reflections | 8773 [R_int_ = 0.0647, R_sigma_ = 0.0663] |
| Data/restraints/parameters | 8773/0/683 |
| Goodness-of-fit on F^2^ | 1.041 |

**Table S3**. Summary of TD-DFT data for **DABNA**, **DABNA-DPA**, **BCzBN** and **BCzBN-3B** at the B3LYP/6-31G(d,p) level.

| Compound | Transition | Wavelength [nm] | Energy [eV] | Oscillator Strength | Coefficient of Orbital |
| --- | --- | --- | --- | --- | --- |
| **DABNA** | S_0_-S_1_ | 396 | 3.13 | 0.2033 | HOMO→LUMO (97.26%) |
|  | S_0_-T_1_ | 470 | 2.64 | 0.0000 | HOMO→LUMO (97.06%) |
|  | S_0_-T_2_ | 392 | 3.16 | 0.0000 | HOMO-3→LUMO+7 (3.41%)  HOMO-2→LUMO (3.17%)  HOMO-1→LUMO (58.46%)  HOMO→LUMO+5 (18.60%)  HOMO→LUMO+7 (5.35%) |
|  | S_0_-T_3_ | 368 | 3.37 | 0.0000 | HOMO-3→LUMO (19.25%)  HOMO-2→LUMO+5 (2.42%)  HOMO-1→LUMO+7 (5.67%)  HOMO→LUMO+1 (18.60%)  HOMO→LUMO+6 (25.79%) |
| **DABNA-3B** | S_0_-S_1_ | 433 | 2.86 | 0.1703 | HOMO-1→LUMO+1 (3.33%)  HOMO→LUMO (95.01%) |
|  | S_0_-T_1_ | 491 | 2.53 | 0.0000 | HOMO→LUMO (95.57%) |
|  | S_0_-T_2_ | 454 | 2.73 | 0.0000 | HOMO-1→LUMO+1 (80.92%)  HOMO→LUMO+1 (8.61%) |
|  | S_0_-T_3_ | 416 | 2.98 | 0.0000 | HOMO-3→LUMO (2.56%)  HOMO-1→LUMO (12.14%)  HOMO→LUMO+1 (74.08%) |
| **BCzBN** | S_0_-S_1_ | 434 | 2.86 | 0.4158 | HOMO→LUMO (98.37%) |
|  | S_0_-T_1_ | 508 | 2.44 | 0.0000 | HOMO→LUMO(93.73%) |
|  | S_0_-T_2_ | 437 | 2.84 | 0.0000 | HOMO-3→LUMO+1 (6.41%)  HOMO-2→LUMO (65.63%)  HOMO-2→LUMO+1 (5.28%)  HOMO-2→LUMO+2 (2.76%)  HOMO-1→LUMO+1 (5.16%)  HOMO→LUMO (2.74%) |
|  | S_0_-T_3_ | 432 | 2.87 | 0.0000 | HOMO-3→LUMO (32.25%)  HOMO-3→LUMO+1 (4.42%)  HOMO-2→LUMO+1 (13.05%)  HOMO-1→LUMO (34.02%) |
| **BCzBN-3B** | S_0_-S_1_ | 446 | 2.78 | 0.2031 | HOMO→LUMO (97.52%) |
|  | S_0_-T_1_ | 502 | 2.47 | 0.0000 | HOMO-2→LUMO (2.12%)  HOMO→LUMO (89.91%) |
|  | S_0_-T_2_ | 452 | 2.74 | 0.0000 | HOMO-3→LUMO (6.50%)  HOMO-2→LUMO+1 (2.35%)  HOMO-1→LUMO (50.02%)  HOMO→LUMO+1 (27.79%) |
|  | S_0_-T_3_ | 444 | 2.79 | 0.0000 | HOMO-4→LUMO (2.59%)  HOMO-3→LUMO+1 (9.95%)  HOMO-2→LUMO (49.32%)  HOMO-2→LUMO+1 (9.88%)  HOMO-2→LUMO+2 (5.24%)  HOMO→LUMO (2.54%) |

**Table S4**. Cartesian coordinates of **DABNA** at the optimized S_0_ geometry.

| \| -------------------------------------------------------------------- \| \| --- \| \| Center Atomic Atomic Coordinates (Angstroms) \| \| Number Number Type X Y Z \| \| ------------------------------------------------------------------- \| \| 1 6 0 -0.067207 -2.149580 2.540181 \| \| 2 6 0 -1.203922 -1.546708 2.163607 \| \| 3 6 0 -1.194174 -0.489942 1.323610 \| \| 4 6 0 0.025117 -0.073119 0.939523 \| \| 5 6 0 1.199642 -0.633232 1.279865 \| \| 6 6 0 1.117188 -1.699207 2.103080 \| \| 7 7 0 2.364949 -0.219329 0.935734 \| \| 8 7 0 -2.310720 0.046266 0.985919 \| \| 9 6 0 -2.374063 1.179777 0.380657 \| \| 10 6 0 -3.593974 1.761451 0.306888 \| \| 11 6 0 3.723517 -1.862450 0.160538 \| \| 12 6 0 2.701260 -0.997680 -0.037589 \| \| 13 6 0 -2.802634 -0.794990 0.137937 \| \| 14 6 0 -2.263574 -1.078234 -1.070647 \| \| 15 6 0 3.794775 1.444095 0.502843 \| \| 16 6 0 2.525604 0.975834 0.488923 \| \| 17 5 0 0.093961 1.229767 0.042136 \| \| 18 6 0 1.566480 1.829220 0.061694 \| \| 19 6 0 -1.552917 3.014492 -0.833854 \| \| 20 6 0 -2.776112 3.556195 -0.908051 \| \| 21 6 0 -3.801170 2.930467 -0.316822 \| \| 22 6 0 4.143165 -2.710917 -0.790608 \| \| 23 6 0 3.545732 -2.723162 -1.989744 \| \| 24 6 0 2.531108 -1.878773 -2.219399 \| \| 25 6 0 2.121628 -1.034455 -1.259294 \| \| 26 6 0 4.110706 2.680792 0.091790 \| \| 27 6 0 3.143561 3.504752 -0.329505 \| \| 28 6 0 1.874285 3.074491 -0.334337 \| \| 29 6 0 -1.346388 1.867961 -0.166930 \| \| 30 6 0 -3.938610 -1.458272 0.455707 \| \| 31 6 0 -4.505654 -2.347306 -0.374162 \| \| 32 6 0 -3.946903 -2.605846 -1.564249 \| \| 33 6 0 -2.821125 -1.966015 -1.909028 \| \| 34 1 0 -0.105737 -3.012705 3.226365 \| \| 35 1 0 -2.152366 -1.945445 2.563864 \| \| 36 1 0 2.027574 -2.211985 2.459431 \| \| 37 1 0 -4.477162 1.279703 0.763255 \| \| 38 1 0 4.249519 -1.889931 1.131862 \| \| 39 1 0 -1.337764 -0.589023 -1.416574 \| \| 40 1 0 4.624492 0.811608 0.866859 \| \| 41 1 0 -0.737557 3.522291 -1.363712 \| \| 42 1 0 -2.940656 4.498202 -1.457115 \| \| 43 1 0 -4.813980 3.364551 -0.365463 \| \| 44 1 0 4.980421 -3.399912 -0.587942 \| \| 45 1 0 3.884508 -3.417645 -2.776165 \| \| 46 1 0 2.032044 -1.880445 -3.203429 \| \| 47 1 0 1.282234 -0.371774 -1.522871 \| \| 48 1 0 5.157825 3.026309 0.116981 \| \| 49 1 0 3.389142 4.530676 -0.651104 \| \| 50 1 0 1.103003 3.784926 -0.653339 \| \| 51 1 0 -4.434897 -1.278542 1.426393 \| \| 52 1 0 -5.432204 -2.867616 -0.078462 \| \| 53 1 0 -4.407214 -3.335938 -2.250506 \| \| 54 1 0 -2.352685 -2.172543 -2.886316 \| \| --------------------------------------------------------------------- \| |
| --- | --- | --- | --- | --- | --- | --- | --- | --- | --- | --- | --- | --- | --- | --- | --- | --- | --- | --- | --- | --- | --- | --- | --- | --- | --- | --- | --- | --- | --- | --- | --- | --- | --- | --- | --- | --- | --- | --- | --- | --- | --- | --- | --- | --- | --- | --- | --- | --- | --- | --- | --- | --- | --- | --- | --- | --- | --- | --- | --- |

**Table S5**. Cartesian coordinates of **DABNA** at the optimized S_1_ geometry.

| -------------------------------------------------------------------- |
| --- |
| Center Atomic Atomic Coordinates (Angstroms) |
| Number Number Type X Y Z |
| ------------------------------------------------------------------- |
| 1 6 0 0.000078 3.000577 -0.000362 |
| 2 6 0 1.218475 2.331128 0.026572 |
| 3 6 0 1.220816 0.926876 0.011898 |
| 4 6 0 0.000020 0.200587 -0.000040 |
| 5 6 0 -1.220735 0.926929 -0.012084 |
| 6 6 0 -1.218358 2.331165 -0.027103 |
| 7 7 0 -2.432284 0.226461 -0.018204 |
| 8 7 0 2.432333 0.226384 0.018159 |
| 9 6 0 2.536746 -1.162142 -0.159088 |
| 10 6 0 3.818949 -1.740479 -0.283933 |
| 11 6 0 -4.267851 1.468027 1.032691 |
| 12 6 0 -3.643519 0.992542 -0.121403 |
| 13 6 0 3.643575 0.992482 0.121367 |
| 14 6 0 4.268504 1.467050 -1.032786 |
| 15 6 0 -3.818962 -1.740292 0.284200 |
| 16 6 0 -2.536736 -1.162042 0.159132 |
| 17 5 0 -0.000003 -1.313196 0.000012 |
| 18 6 0 -1.373589 -1.977964 0.227541 |
| 19 6 0 1.567554 -3.342585 -0.534515 |
| 20 6 0 2.823031 -3.906963 -0.693076 |
| 21 6 0 3.952798 -3.095163 -0.539958 |
| 22 6 0 -5.441155 2.213646 0.926034 |
| 23 6 0 -5.986839 2.483082 -0.330636 |
| 24 6 0 -5.357877 2.006179 -1.481786 |
| 25 6 0 -4.183993 1.259909 -1.378670 |
| 26 6 0 -3.952856 -3.094954 0.540305 |
| 27 6 0 -2.823117 -3.906810 0.693364 |
| 28 6 0 -1.567625 -3.342499 0.534709 |
| 29 6 0 1.373568 -1.978013 -0.227480 |
| 30 6 0 4.183376 1.260868 1.378699 |
| 31 6 0 5.357200 2.007254 1.481851 |
| 32 6 0 5.986762 2.483211 0.330664 |
| 33 6 0 5.441743 2.212734 -0.926095 |
| 34 1 0 0.000099 4.086852 -0.000565 |
| 35 1 0 2.142382 2.891614 0.044410 |
| 36 1 0 -2.142240 2.891683 -0.045128 |
| 37 1 0 4.704745 -1.125810 -0.198082 |
| 38 1 0 -3.827085 1.247521 1.999013 |
| 39 1 0 3.828258 1.245782 -1.999171 |
| 40 1 0 -4.704730 -1.125561 0.198494 |
| 41 1 0 0.694343 -3.966978 -0.677877 |
| 42 1 0 2.929352 -4.960220 -0.932361 |
| 43 1 0 4.948252 -3.517755 -0.641429 |
| 44 1 0 -5.928422 2.583753 1.822566 |
| 45 1 0 -6.900380 3.063648 -0.412276 |
| 46 1 0 -5.780198 2.214659 -2.459701 |
| 47 1 0 -3.678817 0.880537 -2.260435 |
| 48 1 0 -4.948322 -3.517494 0.641873 |
| 49 1 0 -2.929477 -4.960034 0.932772 |
| 50 1 0 -0.694430 -3.966899 0.678151 |
| 51 1 0 3.677718 0.882198 2.260490 |
| 52 1 0 5.778963 2.216521 2.459838 |
| 53 1 0 6.900252 3.063865 0.412252 |
| 54 1 0 5.929509 2.582136 -1.822647 |
| --------------------------------------------------------------------- |

**Table S6**. Cartesian coordinates of **DABNA-3B** at the optimized S_0_ geometry.

| --------------------------------------------------------------------- |
| --- |
| Center Atomic Atomic Coordinates (Angstroms) |
| Number Number Type X Y Z |
| --------------------------------------------------------------------- |
| 1 7 0 2.420932 -2.353808 -0.098483 |
| 2 7 0 -2.503196 -2.242251 -0.075974 |
| 3 7 0 -2.360858 2.037122 0.163560 |
| 4 7 0 2.455452 1.927018 -0.313528 |
| 5 6 0 3.593587 -3.199199 -0.041685 |
| 6 6 0 1.237493 -0.153085 -0.088977 |
| 7 6 0 1.237500 1.256830 -0.162933 |
| 8 6 0 -0.014045 -0.805846 -0.099929 |
| 9 6 0 -1.237290 -0.101553 -0.096543 |
| 10 6 0 -2.307823 3.328409 0.770326 |
| 11 6 0 2.456724 3.248536 -0.840303 |
| 12 6 0 3.773680 -0.071042 0.337965 |
| 13 6 0 4.935331 -0.531478 0.985216 |
| 14 1 0 5.052923 -1.467542 1.096749 |
| 15 6 0 -1.179495 1.296415 0.018426 |
| 16 6 0 1.171338 -3.013689 -0.159614 |
| 17 6 0 -3.603195 1.497136 -0.267291 |
| 18 6 0 -6.083085 0.557903 -1.127293 |
| 19 1 0 -6.940414 0.233736 -1.379332 |
| 20 6 0 -3.699896 -3.052233 0.016705 |
| 21 6 0 -5.080205 -0.327715 -0.749291 |
| 22 1 0 -5.262242 -1.259360 -0.775605 |
| 23 6 0 0.045988 1.992849 -0.049940 |
| 24 6 0 -0.037523 -2.250914 -0.121367 |
| 25 6 0 -4.258453 -3.604811 -1.134553 |
| 26 1 0 -3.836988 -3.479743 -1.976903 |
| 27 6 0 2.489842 5.805470 -1.970540 |
| 28 1 0 2.506478 6.679522 -2.341036 |
| 29 6 0 1.357311 4.103675 -0.644498 |
| 30 6 0 -3.287564 4.973482 2.250730 |
| 31 1 0 -4.007137 5.258489 2.801673 |
| 32 6 0 -1.147078 4.129092 0.636948 |
| 33 6 0 3.655492 1.337869 0.188623 |
| 34 6 0 3.555455 4.931197 -2.172342 |
| 35 1 0 4.294788 5.206637 -2.700271 |
| 36 6 0 -3.367093 3.750827 1.586654 |
| 37 1 0 -4.137731 3.205120 1.686681 |
| 38 6 0 3.548893 3.674746 -1.614119 |
| 39 1 0 4.285939 3.091244 -1.753536 |
| 40 6 0 -4.298567 -3.231275 1.233409 |
| 41 1 0 -3.920455 -2.841486 2.012356 |
| 42 6 0 -1.127276 5.363327 1.318309 |
| 43 1 0 -0.367753 5.927720 1.225618 |
| 44 6 0 5.908363 0.313977 1.463999 |
| 45 1 0 6.671925 -0.034889 1.909774 |
| 46 6 0 -1.275905 -2.958851 -0.100452 |
| 47 6 0 -5.820135 1.899894 -1.134379 |
| 48 1 0 -6.480722 2.508388 -1.442767 |
| 49 6 0 1.408547 5.383734 -1.221825 |
| 50 1 0 0.676824 5.975508 -1.095656 |
| 51 6 0 -1.285263 -4.348287 -0.092902 |
| 52 1 0 -2.107252 -4.816844 -0.004671 |
| 53 6 0 4.658817 2.194373 0.655105 |
| 54 1 0 4.573860 3.132668 0.531634 |
| 55 6 0 1.113864 -4.399428 -0.260693 |
| 56 1 0 1.915299 -4.899331 -0.361919 |
| 57 6 0 -4.586402 2.381738 -0.693439 |
| 58 1 0 -4.418495 3.316443 -0.683934 |
| 59 6 0 5.762595 1.688387 1.288325 |
| 60 1 0 6.434207 2.279697 1.610521 |
| 61 6 0 3.887079 -3.886948 1.136104 |
| 62 1 0 3.309625 -3.812031 1.886573 |
| 63 6 0 -3.811798 0.096598 -0.326792 |
| 64 6 0 -0.099191 -5.050008 -0.214741 |
| 65 1 0 -0.121922 -5.998521 -0.268083 |
| 66 6 0 -6.031824 -4.523685 0.186211 |
| 67 1 0 -6.837080 -5.023009 0.249314 |
| 68 6 0 5.016650 -4.675501 1.209729 |
| 69 1 0 5.204105 -5.166516 2.001274 |
| 70 6 0 -5.432362 -4.337108 -1.044395 |
| 71 1 0 -5.822621 -4.709166 -1.826446 |
| 72 6 0 4.432380 -3.290636 -1.127798 |
| 73 1 0 4.229544 -2.827858 -1.932303 |
| 74 6 0 -5.465816 -3.988423 1.325647 |
| 75 1 0 -5.871314 -4.134759 2.172474 |
| 76 6 0 -2.170273 5.776217 2.112661 |
| 77 1 0 -2.123793 6.609698 2.567041 |
| 78 6 0 5.583132 -4.070748 -1.033706 |
| 79 1 0 6.170244 -4.132049 -1.777338 |
| 80 5 0 -2.569320 -0.818086 -0.141828 |
| 81 5 0 0.074774 3.498080 -0.029944 |
| 82 6 0 5.878532 -4.747191 0.120045 |
| 83 1 0 6.671341 -5.267175 0.176083 |
| 84 5 0 2.527806 -0.941855 0.005818 |
| --------------------------------------------------------------------- |

**Table S7**. Cartesian coordinates of **DABNA-3B** at the optimized S_1_ geometry.

| --------------------------------------------------------------------- |
| --- |
| Center Atomic Atomic Coordinates (Angstroms) |
| Number Number Type X Y Z |
| --------------------------------------------------------------------- |
| 1 7 0 2.461673 -2.321086 -0.017943 |
| 2 7 0 -2.461825 -2.320955 0.017779 |
| 3 7 0 -2.415617 1.981875 0.173129 |
| 4 7 0 2.415695 1.981734 -0.173207 |
| 5 6 0 3.647805 -3.128215 -0.031148 |
| 6 6 0 1.238139 -0.134510 -0.003657 |
| 7 6 0 1.213158 1.273131 -0.062647 |
| 8 6 0 -0.000039 -0.822939 -0.000075 |
| 9 6 0 -1.238187 -0.134439 0.003542 |
| 10 6 0 -2.414090 3.302323 0.709160 |
| 11 6 0 2.414284 3.302167 -0.709211 |
| 12 6 0 3.814037 -0.017534 0.287039 |
| 13 6 0 5.075624 -0.490734 0.713711 |
| 14 1 0 5.256502 -1.554510 0.739315 |
| 15 6 0 -1.213138 1.273192 0.062555 |
| 16 6 0 1.228166 -3.001482 -0.028852 |
| 17 6 0 -3.637863 1.391815 -0.261740 |
| 18 6 0 -6.093077 0.354059 -1.130654 |
| 19 1 0 -7.045355 -0.056602 -1.451118 |
| 20 6 0 -3.647952 -3.128090 0.031132 |
| 21 6 0 -5.075806 -0.490385 -0.713495 |
| 22 1 0 -5.256787 -1.554145 -0.739073 |
| 23 6 0 0.000034 1.997236 -0.000058 |
| 24 6 0 -0.000078 -2.269523 -0.000114 |
| 25 6 0 -4.084936 -3.755863 -1.138905 |
| 26 1 0 -3.497626 -3.648508 -2.044573 |
| 27 6 0 2.446049 5.890916 -1.807499 |
| 28 1 0 2.461016 6.889888 -2.231670 |
| 29 6 0 1.280403 4.138559 -0.559036 |
| 30 6 0 -3.534386 5.029503 1.988249 |
| 31 1 0 -4.393885 5.350639 2.569024 |
| 32 6 0 -1.280166 4.138643 0.558905 |
| 33 6 0 3.637885 1.391584 0.261777 |
| 34 6 0 3.534799 5.029279 -1.988202 |
| 35 1 0 4.394366 5.350361 -2.568910 |
| 36 6 0 -3.519410 3.748229 1.456138 |
| 37 1 0 -4.357362 3.085309 1.630297 |
| 38 6 0 3.519698 3.748014 -1.456096 |
| 39 1 0 4.357625 3.085036 -1.630165 |
| 40 6 0 -4.384813 -3.250542 1.208312 |
| 41 1 0 -4.027606 -2.756741 2.105538 |
| 42 6 0 -1.334915 5.433110 1.115568 |
| 43 1 0 -0.458689 6.067753 1.027941 |
| 44 6 0 6.092900 0.353636 1.130984 |
| 45 1 0 7.045092 -0.057096 1.451618 |
| 46 6 0 -1.228354 -3.001413 0.028608 |
| 47 6 0 -5.863534 1.731422 -1.171455 |
| 48 1 0 -6.624526 2.408176 -1.548290 |
| 49 6 0 1.335276 5.433026 -1.115697 |
| 50 1 0 0.459080 6.067716 -1.028138 |
| 51 6 0 -1.207990 -4.402769 0.061520 |
| 52 1 0 -2.133115 -4.957312 0.118677 |
| 53 6 0 4.647396 2.242765 0.749877 |
| 54 1 0 4.467202 3.307717 0.810850 |
| 55 6 0 1.207725 -4.402837 -0.061866 |
| 56 1 0 2.132821 -4.957428 -0.119036 |
| 57 6 0 -4.647362 2.243059 -0.749761 |
| 58 1 0 -4.467072 3.307994 -0.810792 |
| 59 6 0 5.863465 1.731030 1.171719 |
| 60 1 0 6.624468 2.407726 1.548634 |
| 61 6 0 4.084724 -3.755901 1.138962 |
| 62 1 0 3.497329 -3.648541 2.044574 |
| 63 6 0 -3.814119 -0.017297 -0.286997 |
| 64 6 0 -0.000153 -5.082050 -0.000199 |
| 65 1 0 -0.000188 -6.167815 -0.000224 |
| 66 6 0 -6.019702 -4.597752 0.042299 |
| 67 1 0 -6.946419 -5.162844 0.044606 |
| 68 6 0 5.269430 -4.489092 1.131717 |
| 69 1 0 5.612390 -4.967497 2.043781 |
| 70 6 0 -5.269620 -4.489096 -1.131530 |
| 71 1 0 -5.612634 -4.967567 -2.043540 |
| 72 6 0 4.384771 -3.250705 -1.208262 |
| 73 1 0 4.027614 -2.756978 -2.105548 |
| 74 6 0 -5.574478 -3.979729 1.210883 |
| 75 1 0 -6.153166 -4.062152 2.125500 |
| 76 6 0 -2.445607 5.891075 1.807451 |
| 77 1 0 -2.460466 6.890044 2.231636 |
| 78 6 0 5.574459 -3.979856 -1.210699 |
| 79 1 0 6.153217 -4.062323 -2.125268 |
| 80 5 0 -2.549406 -0.886563 -0.039382 |
| 81 5 0 0.000085 3.502917 -0.000084 |
| 82 6 0 6.019611 -4.597795 -0.042047 |
| 83 1 0 6.946352 -5.162848 -0.044244 |
| 84 5 0 2.549311 -0.886708 0.039277 |
| --------------------------------------------------------------------- |

**Table S8**. Cartesian coordinates of **BCzBN** at the optimized S_0_ geometry.

| -------------------------------------------------------------------- |
| --- |
| Center Atomic Atomic Coordinates (Angstroms) |
| Number Number Type X Y Z |
| ------------------------------------------------------------------- |
| 1 6 0 -0.019314 -3.993408 -0.049284 |
| 2 6 0 -1.173777 -3.322161 -0.028698 |
| 3 6 0 -1.237217 -1.978152 -0.003823 |
| 4 6 0 -0.006351 -1.394640 -0.032496 |
| 5 6 0 1.219074 -1.989726 -0.063490 |
| 6 6 0 1.141848 -3.333264 -0.057218 |
| 7 7 0 2.339907 -1.340453 -0.049579 |
| 8 7 0 -2.351826 -1.318383 0.002281 |
| 9 6 0 2.403583 -0.064140 0.087199 |
| 10 6 0 3.649397 0.412513 0.148984 |
| 11 6 0 4.413017 -0.667170 -0.003652 |
| 12 6 0 3.575817 -1.715239 -0.132077 |
| 13 6 0 -3.589448 -1.682870 0.101854 |
| 14 6 0 -4.420545 -0.627210 -0.004327 |
| 15 6 0 -3.649215 0.446627 -0.161621 |
| 16 6 0 -2.406465 -0.040725 -0.123751 |
| 17 5 0 0.001233 0.192618 -0.041694 |
| 18 6 0 1.426799 0.840714 0.197833 |
| 19 6 0 1.693953 2.137174 0.416260 |
| 20 6 0 2.955237 2.606680 0.490456 |
| 21 6 0 3.944665 1.704876 0.335808 |
| 22 6 0 5.750485 -0.729222 -0.028803 |
| 23 6 0 6.371799 -1.907555 -0.208393 |
| 24 6 0 5.562781 -2.969597 -0.372657 |
| 25 6 0 4.220469 -2.879545 -0.341551 |
| 26 6 0 -3.934817 1.743426 -0.333055 |
| 27 6 0 -2.939870 2.637207 -0.499037 |
| 28 6 0 -1.681873 2.155714 -0.451306 |
| 29 6 0 -1.423156 0.855548 -0.245255 |
| 30 6 0 -4.237922 -2.843824 0.316593 |
| 31 6 0 -5.579631 -2.919863 0.370813 |
| 32 6 0 -6.384353 -1.850886 0.228849 |
| 33 6 0 -5.758521 -0.676071 0.044686 |
| 34 6 0 -3.256537 4.122278 -0.715911 |
| 35 6 0 -4.087640 4.280969 -2.007857 |
| 36 6 0 -4.062098 4.655140 0.490015 |
| 37 6 0 -2.004603 5.019104 -0.858251 |
| 38 6 0 -7.908439 -1.993910 0.304004 |
| 39 6 0 -8.302512 -2.544340 1.692352 |
| 40 6 0 -8.670371 -0.663998 0.102241 |
| 41 6 0 -8.386737 -2.967043 -0.796249 |
| 42 6 0 3.281651 4.087112 0.723932 |
| 43 6 0 2.035696 4.996390 0.837127 |
| 44 6 0 4.078549 4.228305 2.039160 |
| 45 6 0 4.126082 4.619435 -0.455538 |
| 46 6 0 7.901869 -1.989553 -0.241210 |
| 47 6 0 8.434656 -1.124929 -1.404954 |
| 48 6 0 8.469851 -1.465642 1.096658 |
| 49 6 0 8.448330 -3.421385 -0.441789 |
| 50 1 0 -0.024787 -5.098516 -0.057762 |
| 51 1 0 -2.038202 -3.983453 -0.074094 |
| 52 1 0 1.998690 -4.004520 -0.020848 |
| 53 1 0 0.848840 2.821196 0.534535 |
| 54 1 0 4.997652 2.017662 0.380794 |
| 55 1 0 6.338679 0.191628 0.093962 |
| 56 1 0 5.991759 -3.967574 -0.554041 |
| 57 1 0 3.724528 -3.835531 -0.532755 |
| 58 1 0 -4.984989 2.067445 -0.356582 |
| 59 1 0 -0.832390 2.831967 -0.582726 |
| 60 1 0 -3.747975 -3.805077 0.496222 |
| 61 1 0 -6.027978 -3.910433 0.555292 |
| 62 1 0 -6.327620 0.256907 -0.061576 |
| 63 1 0 -4.317908 5.350828 -2.213342 |
| 64 1 0 -3.534472 3.880566 -2.888040 |
| 65 1 0 -5.064597 3.751847 -1.956735 |
| 66 1 0 -4.283994 5.740965 0.382116 |
| 67 1 0 -3.493351 4.520835 1.438367 |
| 68 1 0 -5.043351 4.146203 0.612098 |
| 69 1 0 -1.385276 4.735330 -1.739095 |
| 70 1 0 -2.286543 6.085793 -1.008705 |
| 71 1 0 -1.366449 4.991277 0.054156 |
| 72 1 0 -9.407791 -2.634507 1.794076 |
| 73 1 0 -7.884572 -3.556379 1.887191 |
| 74 1 0 -7.943830 -1.870886 2.503895 |
| 75 1 0 -9.772161 -0.817252 0.154622 |
| 76 1 0 -8.463334 -0.214596 -0.895361 |
| 77 1 0 -8.422690 0.081840 0.891023 |
| 78 1 0 -8.088636 -2.603183 -1.806001 |
| 79 1 0 -7.973291 -3.992193 -0.673348 |
| 80 1 0 -9.495204 -3.072704 -0.790615 |
| 81 1 0 2.324439 6.059617 0.999410 |
| 82 1 0 1.421538 4.978165 -0.091857 |
| 83 1 0 1.390587 4.716194 1.700412 |
| 84 1 0 4.316358 5.294066 2.256998 |
| 85 1 0 5.049672 3.686517 2.011567 |
| 86 1 0 3.496295 3.830106 2.901337 |
| 87 1 0 5.106200 4.102982 -0.552485 |
| 88 1 0 3.583506 4.495262 -1.420521 |
| 89 1 0 4.354177 5.702566 -0.335011 |
| 90 1 0 9.544395 -1.181133 -1.476799 |
| 91 1 0 8.016556 -1.467501 -2.378908 |
| 92 1 0 8.180275 -0.048135 -1.291039 |
| 93 1 0 9.580782 -1.536868 1.121786 |
| 94 1 0 8.075504 -2.056491 1.954801 |
| 95 1 0 8.219956 -0.397790 1.281762 |
| 96 1 0 8.128255 -3.856815 -1.415372 |
| 97 1 0 9.561892 -3.431860 -0.447932 |
| 98 1 0 8.132949 -4.104467 0.379240 |
| --------------------------------------------------------------------- |

**Table S9**. Cartesian coordinates of **BCzBN** at the optimized S_1_ geometry.

| -------------------------------------------------------------------- |
| --- |
| Center Atomic Atomic Coordinates (Angstroms) |
| Number Number Type X Y Z |
| ------------------------------------------------------------------- |
| 1 6 0 -0.022591 -4.068066 -0.015885 |
| 2 6 0 -1.231720 -3.387121 -0.085504 |
| 3 6 0 -1.233340 -1.988580 -0.021212 |
| 4 6 0 -0.007988 -1.252959 -0.008863 |
| 5 6 0 1.209753 -2.001236 0.000458 |
| 6 6 0 1.193460 -3.400153 0.057266 |
| 7 7 0 2.419653 -1.291896 -0.024241 |
| 8 7 0 -2.435835 -1.266515 0.008088 |
| 9 6 0 2.463856 0.089189 0.172281 |
| 10 6 0 3.800986 0.524217 0.274299 |
| 11 6 0 4.631357 -0.649721 0.076694 |
| 12 6 0 3.758014 -1.751515 -0.138624 |
| 13 6 0 -3.778735 -1.711352 0.123298 |
| 14 6 0 -4.639944 -0.602728 -0.084827 |
| 15 6 0 -3.799324 0.563447 -0.280595 |
| 16 6 0 -2.465944 0.115239 -0.183538 |
| 17 5 0 -0.000152 0.288011 -0.006459 |
| 18 6 0 1.365991 0.948561 0.228147 |
| 19 6 0 1.675358 2.305510 0.467263 |
| 20 6 0 2.982951 2.785867 0.604110 |
| 21 6 0 4.051832 1.872415 0.492693 |
| 22 6 0 6.010774 -0.815468 0.018301 |
| 23 6 0 6.568187 -2.066710 -0.275219 |
| 24 6 0 5.685889 -3.126690 -0.535393 |
| 25 6 0 4.295725 -2.990592 -0.480399 |
| 26 6 0 -4.036821 1.915330 -0.493570 |
| 27 6 0 -2.959024 2.818052 -0.604013 |
| 28 6 0 -1.655658 2.324279 -0.471680 |
| 29 6 0 -1.359470 0.963595 -0.238267 |
| 30 6 0 -4.328642 -2.949169 0.463955 |
| 31 6 0 -5.715610 -3.066364 0.522603 |
| 32 6 0 -6.591784 -1.993578 0.268048 |
| 33 6 0 -6.025915 -0.750818 -0.023381 |
| 34 6 0 -3.252125 4.305587 -0.852569 |
| 35 6 0 -4.014043 4.461063 -2.186377 |
| 36 6 0 -4.118452 4.856398 0.300851 |
| 37 6 0 -1.969159 5.150227 -0.931217 |
| 38 6 0 -8.107807 -2.220260 0.341149 |
| 39 6 0 -8.489171 -2.700740 1.758118 |
| 40 6 0 -8.900989 -0.938733 0.038729 |
| 41 6 0 -8.513290 -3.294123 -0.691517 |
| 42 6 0 3.290610 4.269365 0.858711 |
| 43 6 0 2.016045 5.126505 0.938278 |
| 44 6 0 4.051766 4.412161 2.194377 |
| 45 6 0 4.164479 4.815571 -0.291217 |
| 46 6 0 8.094414 -2.225023 -0.323633 |
| 47 6 0 8.672919 -1.287103 -1.405176 |
| 48 6 0 8.688487 -1.849781 1.051236 |
| 49 6 0 8.522771 -3.663512 -0.655635 |
| 50 1 0 -0.028210 -5.153877 -0.018798 |
| 51 1 0 -2.146108 -3.941756 -0.215414 |
| 52 1 0 2.102031 -3.964883 0.183995 |
| 53 1 0 0.851656 2.998769 0.561631 |
| 54 1 0 5.076760 2.219944 0.578502 |
| 55 1 0 6.651062 0.043999 0.190395 |
| 56 1 0 6.079337 -4.099772 -0.801342 |
| 57 1 0 3.680441 -3.836634 -0.746917 |
| 58 1 0 -5.058332 2.273644 -0.575684 |
| 59 1 0 -0.825433 3.009844 -0.565028 |
| 60 1 0 -3.720590 -3.801924 0.725819 |
| 61 1 0 -6.126442 -4.034814 0.788217 |
| 62 1 0 -6.650551 0.118082 -0.192113 |
| 63 1 0 -4.238122 5.515978 -2.379058 |
| 64 1 0 -3.416912 4.079455 -3.020332 |
| 65 1 0 -4.961402 3.914644 -2.176560 |
| 66 1 0 -4.340393 5.916949 0.139399 |
| 67 1 0 -3.597443 4.756937 1.258135 |
| 68 1 0 -5.070988 4.325338 0.382315 |
| 69 1 0 -1.321021 4.827931 -1.752154 |
| 70 1 0 -2.230024 6.198396 -1.106527 |
| 71 1 0 -1.394035 5.102747 -0.001080 |
| 72 1 0 -9.569561 -2.868627 1.826084 |
| 73 1 0 -7.988979 -3.638083 2.016792 |
| 74 1 0 -8.210333 -1.954645 2.508612 |
| 75 1 0 -9.973635 -1.147797 0.093626 |
| 76 1 0 -8.687506 -0.558556 -0.965201 |
| 77 1 0 -8.680581 -0.146343 0.760912 |
| 78 1 0 -8.255797 -2.973660 -1.705773 |
| 79 1 0 -8.008970 -4.246072 -0.503794 |
| 80 1 0 -9.593283 -3.473418 -0.652094 |
| 81 1 0 2.287078 6.171359 1.117867 |
| 82 1 0 1.442111 5.088133 0.006995 |
| 83 1 0 1.363290 4.807695 1.756912 |
| 84 1 0 4.286218 5.464017 2.391347 |
| 85 1 0 4.993579 3.856221 2.184169 |
| 86 1 0 3.449375 4.033549 3.025906 |
| 87 1 0 5.111664 4.275062 -0.373161 |
| 88 1 0 3.644078 4.725081 -1.249714 |
| 89 1 0 4.397052 5.873162 -0.125398 |
| 90 1 0 9.763157 -1.383772 -1.451219 |
| 91 1 0 8.264928 -1.532985 -2.390431 |
| 92 1 0 8.438872 -0.239007 -1.198883 |
| 93 1 0 9.779297 -1.949722 1.036495 |
| 94 1 0 8.294859 -2.503688 1.835552 |
| 95 1 0 8.450645 -0.818386 1.326089 |
| 96 1 0 8.161303 -3.977417 -1.639857 |
| 97 1 0 9.615030 -3.728216 -0.668340 |
| 98 1 0 8.156408 -4.376952 0.089148 |
| --------------------------------------------------------------------- |

**Table S10**. Cartesian coordinates of **BCzBN-3B** at the optimized S_0_ geometry.

| -------------------------------------------------------------------- |
| --- |
| Center Atomic Atomic Coordinates (Angstroms) |
| Number Number Type X Y Z |
| -------------------------------------------------------------------- |
| 1 7 0 2.339638 0.884736 0.058071 |
| 2 7 0 -2.371909 0.798958 -0.347042 |
| 3 7 0 -2.420811 -3.450049 -0.000535 |
| 4 7 0 2.501294 -3.376415 0.188405 |
| 5 6 0 -0.010340 0.930947 -0.178021 |
| 6 6 0 -1.172647 0.138360 -0.187290 |
| 7 6 0 -1.216171 -1.258785 -0.037612 |
| 8 6 0 0.023204 -1.934326 0.086856 |
| 9 6 0 0.039066 -3.376146 0.147943 |
| 10 6 0 -1.173983 -4.121042 0.093136 |
| 11 6 0 -1.130627 -5.521128 0.113449 |
| 12 1 0 -1.935934 -6.022765 0.066762 |
| 13 6 0 0.090685 -6.174670 0.201473 |
| 14 1 0 0.106196 -7.123406 0.235648 |
| 15 6 0 1.280598 -5.482266 0.240958 |
| 16 1 0 2.102949 -5.954914 0.294984 |
| 17 6 0 1.279889 -4.087443 0.201070 |
| 18 6 0 1.240121 -1.221827 0.140402 |
| 19 6 0 1.165239 0.180842 0.016614 |
| 20 6 0 3.605499 0.321521 0.190963 |
| 21 6 0 3.873808 -1.043314 0.257972 |
| 22 6 0 5.251574 -1.339051 0.306688 |
| 23 1 0 5.516622 -2.249792 0.360310 |
| 24 6 0 6.256755 -0.347669 0.279819 |
| 25 6 0 5.895222 1.002910 0.230484 |
| 26 1 0 6.567583 1.673953 0.231146 |
| 27 6 0 4.555957 1.364528 0.180609 |
| 28 6 0 3.798353 2.619469 0.067291 |
| 29 6 0 4.097819 3.977417 0.086540 |
| 30 1 0 5.000378 4.262847 0.164864 |
| 31 6 0 3.062762 4.932893 -0.010049 |
| 32 6 0 1.745405 4.498020 -0.176172 |
| 33 1 0 1.066512 5.153171 -0.286494 |
| 34 6 0 1.369110 3.142200 -0.188421 |
| 35 6 0 2.444086 2.260359 -0.028176 |
| 36 6 0 3.401225 6.419624 0.174299 |
| 37 6 0 4.594471 6.827451 -0.691446 |
| 38 1 0 5.372904 6.281937 -0.452155 |
| 39 1 0 4.799747 7.773547 -0.539836 |
| 40 1 0 4.375606 6.687606 -1.636148 |
| 41 6 0 3.746943 6.644417 1.658610 |
| 42 1 0 2.981590 6.388396 2.214800 |
| 43 1 0 3.956063 7.590279 1.806036 |
| 44 1 0 4.523292 6.096774 1.899875 |
| 45 6 0 2.223460 7.341556 -0.186004 |
| 46 1 0 1.948332 7.174288 -1.111473 |
| 47 1 0 2.500508 8.277383 -0.091933 |
| 48 1 0 1.471440 7.162032 0.416135 |
| 49 6 0 7.734130 -0.764348 0.273305 |
| 50 6 0 8.695213 0.367011 0.010385 |
| 51 1 0 8.437082 0.830189 -0.813791 |
| 52 1 0 9.602515 0.009627 -0.086229 |
| 53 1 0 8.670651 0.997171 0.760616 |
| 54 6 0 8.055594 -1.653675 1.317377 |
| 55 1 0 8.035384 -1.174275 2.172026 |
| 56 1 0 8.952454 -2.020183 1.169881 |
| 57 1 0 7.403124 -2.384412 1.337116 |
| 58 6 0 7.900375 -1.671776 -1.161302 |
| 59 1 0 7.286057 -2.435118 -1.138278 |
| 60 1 0 8.821871 -1.996508 -1.237699 |
| 61 1 0 7.688563 -1.105310 -1.932429 |
| 62 6 0 -1.456222 3.085301 -0.449928 |
| 63 6 0 -2.512201 2.175134 -0.446176 |
| 64 6 0 -3.883244 2.495329 -0.422611 |
| 65 6 0 -4.600246 1.219281 -0.263352 |
| 66 6 0 -5.914963 0.844083 -0.043534 |
| 67 1 0 -6.606849 1.494720 -0.070068 |
| 68 6 0 -6.224275 -0.507678 0.219901 |
| 69 6 0 -5.196928 -1.465797 0.213588 |
| 70 1 0 -5.428721 -2.372796 0.374504 |
| 71 6 0 -3.842512 -1.156373 -0.018682 |
| 72 6 0 -3.615970 0.210915 -0.219848 |
| 73 6 0 -7.656036 -0.889662 0.627078 |
| 74 6 0 -7.967013 -2.365518 0.396097 |
| 75 1 0 -8.896679 -2.547527 0.647796 |
| 76 1 0 -7.367452 -2.915780 0.942594 |
| 77 1 0 -7.836813 -2.583101 -0.550683 |
| 78 6 0 -7.791240 -0.570398 2.119527 |
| 79 1 0 -8.708940 -0.753008 2.410815 |
| 80 1 0 -7.581880 0.374619 2.271590 |
| 81 1 0 -7.169046 -1.129257 2.630641 |
| 82 6 0 -8.698174 -0.081163 -0.130243 |
| 83 1 0 -8.534510 -0.157843 -1.093268 |
| 84 1 0 -8.639052 0.859882 0.137238 |
| 85 1 0 -9.592264 -0.424153 0.077679 |
| 86 6 0 -4.224127 3.840151 -0.418491 |
| 87 1 0 -5.138634 4.097320 -0.419997 |
| 88 6 0 -3.217206 4.824442 -0.412066 |
| 89 6 0 -1.880229 4.430739 -0.448152 |
| 90 1 0 -1.215117 5.108753 -0.472912 |
| 91 6 0 -3.596516 6.304266 -0.195567 |
| 92 6 0 -3.831129 6.497214 1.303230 |
| 93 1 0 -4.557789 5.910553 1.598808 |
| 94 1 0 -4.073579 7.431139 1.478166 |
| 95 1 0 -3.011867 6.275910 1.793493 |
| 96 6 0 -4.875375 6.675942 -0.943339 |
| 97 1 0 -4.754015 6.508941 -1.901413 |
| 98 1 0 -5.073264 7.625288 -0.800032 |
| 99 1 0 -5.619245 6.133195 -0.608663 |
| 100 6 0 -2.492588 7.266631 -0.637959 |
| 101 1 0 -1.696661 7.127773 -0.083705 |
| 102 1 0 -2.804446 8.189919 -0.536997 |
| 103 1 0 -2.269831 7.097994 -1.577565 |
| 104 6 0 -3.611946 -4.234995 -0.195209 |
| 105 6 0 -4.207610 -4.260583 -1.448537 |
| 106 1 0 -3.784254 -3.833252 -2.183860 |
| 107 6 0 -5.417403 -4.907581 -1.628963 |
| 108 1 0 -5.831003 -4.906003 -2.483990 |
| 109 6 0 -6.028923 -5.558415 -0.569522 |
| 110 1 0 -6.862161 -5.997252 -0.693911 |
| 111 6 0 -5.418738 -5.562936 0.664907 |
| 112 1 0 -5.824422 -6.025133 1.389009 |
| 113 6 0 -4.214218 -4.897819 0.860942 |
| 114 1 0 -3.805180 -4.897497 1.717996 |
| 115 6 0 3.718802 -4.126210 -0.045132 |
| 116 6 0 4.178390 -4.251415 -1.339943 |
| 117 1 0 3.647798 -3.944573 -2.065599 |
| 118 6 0 5.416064 -4.826514 -1.578558 |
| 119 1 0 5.735637 -4.904428 -2.469658 |
| 120 6 0 6.185505 -5.284443 -0.536537 |
| 121 1 0 7.039811 -5.662959 -0.706008 |
| 122 6 0 5.713827 -5.191590 0.761337 |
| 123 1 0 6.236254 -5.519447 1.483748 |
| 124 6 0 4.468743 -4.616919 1.001161 |
| 125 1 0 4.135710 -4.562392 1.889585 |
| 126 5 0 -0.030836 2.467561 -0.307256 |
| 127 5 0 -2.529900 -2.019344 -0.013376 |
| 128 5 0 2.585723 -1.949337 0.227759 |
| --------------------------------------------------------------------- |

**Table S11**. Cartesian coordinates of **BCzBN-3B** at the optimized S_1_ geometry.

| -------------------------------------------------------------------- |
| --- |
| Center Atomic Atomic Coordinates (Angstroms) |
| Number Number Type X Y Z |
| ------------------------------------------------------------------- |
| 1 7 0 2.357054 0.900477 0.000041 |
| 2 7 0 -2.375043 0.850256 -0.000354 |
| 3 7 0 -2.428133 -3.419633 -0.000217 |
| 4 7 0 2.498402 -3.366172 0.000037 |
| 5 6 0 -0.010028 0.966574 -0.000126 |
| 6 6 0 -1.183338 0.181190 -0.000218 |
| 7 6 0 -1.220840 -1.228834 -0.000197 |
| 8 6 0 0.019448 -1.912363 -0.000117 |
| 9 6 0 0.035276 -3.354237 -0.000121 |
| 10 6 0 -1.189741 -4.093122 -0.000200 |
| 11 6 0 -1.153665 -5.494374 -0.000259 |
| 12 1 0 -2.075606 -6.059003 -0.000330 |
| 13 6 0 0.066773 -6.158022 -0.000208 |
| 14 1 0 0.078339 -7.243793 -0.000266 |
| 15 6 0 1.272420 -5.467950 -0.000104 |
| 16 1 0 2.207709 -6.010656 -0.000020 |
| 17 6 0 1.276086 -4.066797 -0.000061 |
| 18 6 0 1.244518 -1.202316 -0.000048 |
| 19 6 0 1.179195 0.206655 -0.000046 |
| 20 6 0 3.620007 0.328116 0.000020 |
| 21 6 0 3.876656 -1.048783 0.000061 |
| 22 6 0 5.251596 -1.357397 0.000061 |
| 23 1 0 5.550508 -2.395565 0.000100 |
| 24 6 0 6.267564 -0.382857 0.000018 |
| 25 6 0 5.926469 0.983281 -0.000040 |
| 26 1 0 6.698849 1.742824 -0.000116 |
| 27 6 0 4.584677 1.360031 -0.000063 |
| 28 6 0 3.836917 2.626781 -0.000090 |
| 29 6 0 4.145936 3.980424 -0.000237 |
| 30 1 0 5.183532 4.299156 -0.000261 |
| 31 6 0 3.112241 4.948005 -0.000335 |
| 32 6 0 1.774275 4.529837 -0.000304 |
| 33 1 0 0.996141 5.280675 -0.000479 |
| 34 6 0 1.384821 3.170136 -0.000144 |
| 35 6 0 2.463388 2.279877 -0.000043 |
| 36 6 0 3.499734 6.436278 -0.000364 |
| 37 6 0 4.335352 6.748637 -1.260723 |
| 38 1 0 5.251507 6.152703 -1.298270 |
| 39 1 0 4.623605 7.805449 -1.276301 |
| 40 1 0 3.761430 6.536347 -2.168053 |
| 41 6 0 4.334947 6.748614 1.260300 |
| 42 1 0 3.760693 6.536325 2.167415 |
| 43 1 0 4.623248 7.805413 1.275986 |
| 44 1 0 5.251040 6.152596 1.298110 |
| 45 6 0 2.272627 7.363583 -0.000584 |
| 46 1 0 1.649434 7.212503 -0.887553 |
| 47 1 0 2.601953 8.407158 -0.000764 |
| 48 1 0 1.649362 7.212872 0.886402 |
| 49 6 0 7.729821 -0.860814 0.000068 |
| 50 6 0 8.728019 0.307937 0.000076 |
| 51 1 0 8.613902 0.938668 -0.887364 |
| 52 1 0 9.750770 -0.081266 0.000117 |
| 53 1 0 8.613854 0.938720 0.887459 |
| 54 6 0 7.988230 -1.717280 1.259325 |
| 55 1 0 7.806612 -1.134813 2.168111 |
| 56 1 0 9.027554 -2.064123 1.277046 |
| 57 1 0 7.340591 -2.597471 1.288298 |
| 58 6 0 7.988336 -1.717311 -1.259156 |
| 59 1 0 7.340553 -2.597396 -1.288291 |
| 60 1 0 9.027603 -2.064339 -1.276674 |
| 61 1 0 7.807005 -1.134786 -2.167971 |
| 62 6 0 -1.449342 3.140264 -0.000072 |
| 63 6 0 -2.509272 2.227945 -0.000166 |
| 64 6 0 -3.888678 2.547965 -0.000026 |
| 65 6 0 -4.611435 1.266554 -0.000100 |
| 66 6 0 -5.939526 0.862075 0.000055 |
| 67 1 0 -6.732550 1.603173 0.000197 |
| 68 6 0 -6.258695 -0.514528 0.000038 |
| 69 6 0 -5.229230 -1.468213 -0.000072 |
| 70 1 0 -5.497672 -2.512706 -0.000002 |
| 71 6 0 -3.855722 -1.128310 -0.000224 |
| 72 6 0 -3.625235 0.250586 -0.000267 |
| 73 6 0 -7.741065 -0.925259 0.000085 |
| 74 6 0 -7.927638 -2.451394 0.000089 |
| 75 1 0 -8.996162 -2.689060 0.000297 |
| 76 1 0 -7.481427 -2.916980 0.883475 |
| 77 1 0 -7.481790 -2.916957 -0.883483 |
| 78 6 0 -8.429559 -0.358048 1.260389 |
| 79 1 0 -9.488740 -0.637650 1.276290 |
| 80 1 0 -8.372384 0.733448 1.298776 |
| 81 1 0 -7.958301 -0.749302 2.167391 |
| 82 6 0 -8.429459 -0.358039 -1.260317 |
| 83 1 0 -7.957850 -0.749001 -2.167246 |
| 84 1 0 -8.372563 0.733485 -1.298454 |
| 85 1 0 -9.488556 -0.637947 -1.276498 |
| 86 6 0 -4.225852 3.894528 0.000150 |
| 87 1 0 -5.269859 4.191597 0.000267 |
| 88 6 0 -3.212255 4.883195 0.000219 |
| 89 6 0 -1.866413 4.491827 0.000147 |
| 90 1 0 -1.103390 5.257995 0.000315 |
| 91 6 0 -3.629957 6.363194 0.000478 |
| 92 6 0 -4.471659 6.658020 1.261048 |
| 93 1 0 -5.375436 6.043445 1.298455 |
| 94 1 0 -4.781472 7.708697 1.276948 |
| 95 1 0 -3.893437 6.457239 2.168257 |
| 96 6 0 -4.471602 6.658535 -1.259989 |
| 97 1 0 -3.893310 6.458262 -2.167268 |
| 98 1 0 -4.781533 7.709183 -1.275414 |
| 99 1 0 -5.375314 6.043887 -1.297778 |
| 100 6 0 -2.421967 7.315226 0.000693 |
| 101 1 0 -1.795913 7.176923 0.887729 |
| 102 1 0 -2.772443 8.351877 0.000752 |
| 103 1 0 -1.795719 7.177072 -0.886222 |
| 104 6 0 -3.618594 -4.221815 -0.000173 |
| 105 6 0 -4.209464 -4.590003 -1.209881 |
| 106 1 0 -3.736007 -4.290466 -2.138742 |
| 107 6 0 -5.400519 -5.315845 -1.208390 |
| 108 1 0 -5.863630 -5.593278 -2.150110 |
| 109 6 0 -5.998586 -5.677954 -0.000047 |
| 110 1 0 -6.929193 -6.236427 0.000007 |
| 111 6 0 -5.400362 -5.315891 1.208235 |
| 112 1 0 -5.863367 -5.593355 2.149998 |
| 113 6 0 -4.209309 -4.590052 1.209601 |
| 114 1 0 -3.735726 -4.290557 2.138412 |
| 115 6 0 3.712824 -4.131039 0.000217 |
| 116 6 0 4.321589 -4.468815 -1.209533 |
| 117 1 0 3.834208 -4.192802 -2.138426 |
| 118 6 0 5.552671 -5.124666 -1.208150 |
| 119 1 0 6.031131 -5.374752 -2.149782 |
| 120 6 0 6.172695 -5.448011 0.000569 |
| 121 1 0 7.135171 -5.949533 0.000704 |
| 122 6 0 5.552486 -5.124367 1.209109 |
| 123 1 0 6.030798 -5.374217 2.150879 |
| 124 6 0 4.321399 -4.468517 1.210139 |
| 125 1 0 3.833876 -4.192280 2.138891 |
| 126 5 0 -0.025495 2.510021 -0.000114 |
| 127 5 0 -2.544359 -1.986118 -0.000219 |
| 128 5 0 2.582352 -1.931422 0.000038 |
| --------------------------------------------------------------------- |

**Table S12.** Summary of photoluminescence spectra data in different polar solvents.

| Solvent | **DABNA** | | **DABNA-3B** | | **BCzBN** | | **BCzBN-3B** | |
| --- | --- | --- | --- | --- | --- | --- | --- | --- |
|  | *λ*_em_  [nm] | FWHM  [nm/eV] | *λ*_em_  [nm] | FWHM  [nm/eV] | *λ*_em_  [nm] | FWHM  [nm/eV] | *λ*_em_  [nm] | FWHM  [nm/eV] |
| *n*-hexane | 459 | 29/0.17 | 459 | 15/0.09 | 474 | 18/0.10 | 468 | 8/0.05 |
| toluene | 458 | 29/0.17 | 470 | 19/0.11 | 484 | 23/0.12 | 482 | 16/0.09 |
| THF | 460 | 29/0.17 | 470 | 22/0.13 | 483 | 25/0.13 | 482 | 22/0.12 |
| MeCN | 465 | 32/0.18 | 474 | 32/0.18 | 490 | 32/0.16 | 494 | 35/0.18 |

**Table S13.** Summary of transient PL performance for emitters doped in mCBP film (2 wt%) at 300 K.

| Compound | *τ*_p_^[a]^  [ns] | *τ*_d_^[a]^  [μs] | *Φ*_p_^[b]^  [%] | *Φ*_d_^[b]^  [%] | *k*_r_^[c]^  [10^7^ s^-1^] | *k*_nr_^[c]^  [10^6^ s^-1^] | *k*_ISC_^[c]^  [10^7^ s^-1^] | *k*_RISC_^[c]^  [10^5^ s^-1^] |
| --- | --- | --- | --- | --- | --- | --- | --- | --- |
| **DABNA** | 8.80 | 93.7 | 84 | 4 | 9.60 | 13.09 | 0.45 | 0.11 |
| **DABNA-3B** | 1.79 | 6.10 | 18 | 76 | 9.98 | 6.37 | 45.25 | 8.63 |
| **BCzBN** | 4.96 | 67.0 | 73 | 23 | 14.79 | 6.16 | 4.76 | 0.20 |
| **BCzBN-3B** | 7.04 | 25.4 | 27 | 72 | 3.80 | 0.38 | 10.37 | 1.46 |

[a] prompt lifetime (*τ*_p_) and delayed lifetime (*τ*_d_); [b] prompt fluorescence quantum yield (*Φ*_p_) and delayed fluorescence quantum yield (*Φ*_d_); [c] rate constants of singlet radiative decay (*k*_r_), non-radiative decay (*k*_nr_), intersystem crossing (*k*_ISC_) and reverse intersystem crossing (*k*_RISC_)

**Table S14.** Summary of heavy-atom-free MR-TADF emitters with *k*_RISC_ over 1 × 10^5^ s^-1^.

| Emitter | *λ*_em_  [nm] | FWHM  [nm] | *k*_r_  [× 10^8^ s^-1^] | *k*_RISC_  [× 10^5^ s^-1^] | Ref. |
| --- | --- | --- | --- | --- | --- |
| ***ν*-DABNA** | 468 | 14 | 2.0 | 2.0 | *Nat. Photonics* **2019**, *13*, 678. |
| **BBCZ-G** | 517 | 34 | 0.58 | 1.8 | *J. Am. Chem. Soc.* **2020**, *142*, 19468. |
| **BBCZ-Y** | 549 | 42 | 0.47 | 1.0 |  |
| ***m*-Cz-BNCZ** | 519 | 38 | 0.6 | 10.8 | *Angew. Chem. Int. Ed*. **2020**, *59*, 17442. |
| ***ν*-DABNA-O-Me** | 461 | 19 | 1.4 | 1.6 | *Angew. Chem. Int. Ed.* **2021**, *60*, 17910. |
| **CNCz-BNCZ** | 581 | 42 | 0.22 | 4.2 | *Chem. Sci.* **2021**, *12*, 9408. |
| **BN-DPAC** | 490 | 30 | 1.3 | 1.26 | *Adv. Opt. Mater.* **2021**, *9*, 2100825. |
| **V-DABNA-Mes** | 486 | 13 | 1.1 | 4.4 | *J. Am. Chem. Soc.* **2022**, *144*, 106. |
| ***ω*-DABNA** | 509 | 18 | 1.4 | 1.2 | *J. Am. Chem. Soc*. **2022**, *145*, 1505. |
| **BN3** | 456 | 17 | 1.7 | 2.55 | *Angew. Chem. Int. Ed.* **2022**, *61*, e202201588. |
| **BN2** | 567 | 97 | 0.013 | 2.10 | *Chem. Sci.* *13*, **2022**, 1665. |
| **TCz-BN2** | 584 | 108 | 0.015 | 2.44 |  |
| **TRZCzPh-BNCZ** | 514 | 34 | 2.45 | 21.3 | *Angew. Chem. Int. Ed*. **2022**, *61*, e202210210. |
| **TRZTPh-BNCz** | 513 | 29 | 2.42 | 15.5 |  |
| **VTCzBN** | 496 | 34 | 0.30 | 10.0 | *Angew. Chem. Int. Ed.* **2022**, *61*, e202209984. |
| **TCz-VTCzBN** | 521 | 29 | 0.11 | 9.0 |  |
| ***ν*-DABNA-CN-Me** | 496 | 17 | 1.7 | 1.0 | *Adv. Mater.* **2022**, *34*, 2201778. |
| **V-DABNA** | 483 | 14 | 1.2 | 5.7 | *Adv. Sci.* **2022**, *10*, 2205070. |
| **V-DABNA-F** | 467 | 13 | 1.1 | 6.5 |  |
| **m-*ν*-DABNA** | 464 | 14 | 0.93 | 2.30 | *Chem. Eng. J.* **2022**, *432*, 134381. |
| **4F-*ν*-DABNA** | 457 | 14 | 0.89 | 2.28 |  |
| **4F-m-*ν*-DABNA** | 455 | 14 | 0.94 | 2.10 |  |
| **DPXZCZBN** | 500 | 32 | 1.0 | 1.11 | *J. Mater. Chem. C.* **2022**, *10*, 768. |
| **NBO** | 487 | 27 | 0.25 | 9.3 | *Adv. Opt. Mater.* **2022**, *10*, 2102513. |
| **NBNP** | 500 | 29 | 0.85 | 3.0 |  |
| **DBTN-2** | 512 | 20 | 2.7 | 1.7 | *Nat. Photonics* **2023**, *17*, 280. |
| **CzB4** | 483 | 14 | 1.6 | 1.8 | *J. Am. Chem. Soc.* **2023**, *145*, 11504. |
| **CzB6** | 488 | 12 | 2.6 | 3.0 |  |
| **CzB8** | 491 | 12 | 2.6 | 6.5 |  |
| **DTBA-BN2** | 490 | 41 | 1.3 | 2.3 | *Angew. Chem. Int. Ed.* **2023**, *62*, e202218405. |
| **DTBA-B2N3** | 471 | 23 | 2.2 | 16 |  |
| **NO-DBMR** | 458 | 14 | 1.09 | 1.01 | *Angew. Chem., Int. Ed.* **2023**, e202306768. |
| **Cz-DBMR** | 480 | 14 | 0.87 | 3.72 |  |
| ***m*-DBCz** | 541 | 32 | 1.04 | 1.65 | *Angew. Chem., Int. Ed.* **2023**,  *62*, e202304104. |
| **TPD4PA** | 445 | 19 | 0.76 | 2.51 | *Chem. Eng. J*. **2023**, *451*, 138498. |
| **tBu-TPD4PA** | 451 | 19 | 0.71 | 2.44 |  |
| **DABNA-3B** | 470 | 19 | 1.0 | 8.63 | **This work** |
| **BCzBN-3B** | 482 | 16 | 0.38 | 1.46 | **This work** |

**Table S15**. Summary of binary-EML TADF-OLED performances with EQE_max_ over 30%.

| Emitter | Host | EL_peak_  [nm] | FWHM  [nm] | EQE_max/100/1000_  [%] | Ref. |
| --- | --- | --- | --- | --- | --- |
| **OBOtSAc** | DPEPO | 452 | 50 | 31.2/-/- | *Adv. Opt. Mater.* **2021**, *9*, 2100406. |
| **TPD4PA** | mCBP-CN | 455 | 29 | 30.7/30.6/17.8 | *Chem. Eng. J.* **2023**, *451*, 138498. |
| **tBu-TPD4PA** | mCBP-CN | 460 | 29 | 32.5/30.9/20.5 | *Chem. Eng. J.* **2023**, *451*, 138498. |
| **4F-m-*ν*-DABNA** | DBFPO | 461 | 18 | 33.7/-/- | *Chem. Eng. J.* **2022**, *432*, 134381. |
| **4F-*ν*-DABNA** | DBFPO | 464 | 18 | 35.8/-/- | *Chem. Eng. J.* **2022**, *432*, 134381. |
| **3DPyM-pDTC** | mCBP | 464 | 62 | 31.9/-/- | *J. Am. Chem. Soc.* **2017**, *139*, 10948-10951. |
| **c-NN-MeTRZ** | mCPCN | 467 | - | 32.2/-/- | *Adv. Opt. Mater.* **2023**, *11*, 2202292. |
| **OBO-II** | PPF | 468 | 59 | 33.8/32.7/26.2 | *Adv. Opt. Mater.* **2021**, *9*, 2101282. |
| ***ν*-DABNA** | DOBNA-OAr | 469 | 18 | 34.4/32.8/26.0 | *Nat. Photonics* **2019**, *13*, 678-682. |
| **2PCzBN-FPh** | PPF | 469 | - | 35.7/33.0/24.8 | *Chem.* **2022**, *8*, 1705-1719. |
| **NO-DBMR** | DBFPO | 469 | 26 | 33.7/-/- | *Angew. Chem. Int. Ed.* **2023**, *62*, e202306768. |
| **m-*ν*-DABNA** | DBFPO | 471 | 18 | 36.2/-/- | *Chem. Eng. J.* **2022**, *432*, 134381. |
| **sAC-BAsBP** | DPEPO | 472 | 57 | 36.4/32.4/26.3 | *Chem. Eng. J.* **2023**, *452*, 139387. |
| **tCBNDADPO** | DBFDPO | 472 | 28 | 30.8/-/- | *Adv. Mater.* **2022**, *34*, 2110547. |
| **mMDBA-DI** | DBFPO | 474 | 60 | 32.8/-/28.4 | *Adv. Funct. Mater.* **2022**, *32*, 2110356. |
| **TBN-TPA** | 2,6-DCzppy | 474 | 27 | 31.1/27.1/13.9 | *Angew. Chem. Int. Ed.* **2018**, *57*, 11316-11320. |
| **DTBA-B2N3** | 2,6-DCzppy | 475 | 28 | 30.9/27.4/20.5 | *Angew. Chem. Int. Ed.* **2023**, *135*, e202218045. |
| **DBA–BFICz** | DBFPO | 476 | 64 | 33.2/-/23.6 | *Adv. Funct. Mater.* **2021**, *31*, 2105805. |
| **DBA–BTICz** | DBFPO | 476 | 64 | 32.8/-/23.4 | *Adv. Funct. Mater.* **2021**, *31*, 2105805. |
| **M3CzB** | DBFPO | 478 | - | 30.7/-/21.6 | *J. Mater. Chem. C* **2020**, *8*, 2272-2279. |
| **BuCzMeoB** | DPEPO | 479 | - | 32.8/29.7/23.2 | *Adv. Opt. Mater.* **2018**, *6*, 1800385. |
| **SpiroAC-TRZ** | mCPCN | 480 | - | 36.7/34.9/30.5 | *Adv. Mater.* **2016**, *28*, 6976-6983. |
| **CzBN2** | DMIC-TRZ | 481 | 75 | 32.4/-/30.2 | *J. Am. Chem. Soc.* **2023**, *145*, 12550-12560. |
| **TspiroS-TRZ** | DPEPO | 481 | - | 33.3/31.1/23.3 | *Angew. Chem. Int. Ed.* **2019**, *131*, 11423-11427. |
| **PTZBN2** | 2,6-DCzppy | 483 | 43 | 30.5/29.7/23.0 | *Adv. Funct. Mater.* **2022**, *32*, 2201032. |
| **CzBN3** | DMIC-TRZ | 483 | 25 | 36.4/-/30.3 | *J. Am. Chem. Soc.* **2023**, *145*, 12550-12560. |
| **BO-3DPAC** | DPEPO | 484 | 71 | 38.7/-/20.3 | *Aggregate.* **2023**, *00*, e382. |
| **PhCzSpiroS-TRZ** | PPF | 490 | 75 | 33.6/-/- | *Adv. Opt. Mater.* **2023**, *11*, 2300017. |
| **SF1BN** | mCBP | 492 | 27 | 35.9/26.9/14.0 | *Angew. Chem. Int. Ed.* **2022**, *61*, e202201886. |
| **QAC-TRZ** | PPF | 494 | - | 37.3/36.6/31.7 | *Angew. Chem. Int. Ed.* **2023**, *135*, e202217080. |
| **3PyBN** | DMIC-TRZ | 494 | 27 | 33.0/-/15.3 | *Chem. Sci.* **2023**, *14*, 3326-3331. |
| **Π-CzBN** | mCP | 495 | 21 | 38.0/-/18.4 | *Angew. Chem. Int. Ed.* **2023**, *135*, e202306413. |
| **BN-CP1** | DMIC-TRZ | 496 | 25 | 40.0/34.0/18.5 | *Adv. Mater.* **2022**, *34*, 2106954. |
| **TCzTrz** | DPEPO | 496 | - | 31.8/-/11.3 | *Adv. Opt. Mater.* **2018**, *6*, 1701340. |
| **8FDM-B** | PPF | 496 | 73 | 31.7/30.1/26.1 | *Adv. Funct. Mater.* **2023**, *33*, 2209708. |
| **SF3BN** | mCBP | 496 | 30 | 32.2/23.9/10.3 | *Angew. Chem. Int. Ed.* **2022**, *61*, e202201886. |
| **BN-CP2** | DMIC-TRZ | 497 | 26 | 36.4/32.6/19.2 | *Adv. Mater.* **2022**, *34*, 2106954. |
| **DTBA-BN2** | 2,6-DCzppy | 497 | 47 | 31.2/28.2/25.6 | *Angew. Chem. Int. Ed.* **2023**, *135*, e202218045. |
| **4PyBN** | DMIC-TRZ | 501 | 27 | 35.5/-/16.6 | *Chem. Sci.* **2023**, *14*, 3326-3331. |
| **(S)-BN-MeIAc** | DMIC-TRZ | 503 | 33 | 36.1/35.2/25.1 | *Angew. Chem. Int. Ed.* **2022**, *134*, e202202227. |
| **(R)-BN-MeIAc** | DMIC-TRZ | 504 | 33 | 37.2/36.1/26.1 | *Angew. Chem. Int. Ed.* **2022**, *134*, e202202227. |
| **2tDMG** | DPEPO | 504 | - | 30.8/28.5/24.6 | *Adv. Mater.* **2020**, *32*, 2003885. |
| **4CzIPN** | o-3CbzBz | 504 | - | 31.8/-/27.7 | *Adv. Opt. Mater.* **2021**, *9*, 2100587. |
| **ν-DABNA-CN-Me** | DOBNA-Ph | 504 | 23 | 31.9/31.5/28.5 | *Adv. Mater.* **2022**, *34*, 2201778. |
| **2PyBN** | DMIC-TRZ | 505 | 26 | 37.1/-/18.0 | *Chem. Sci.* **2023**, *14*, 3326-3331. |
| **BN-Se** | DMIC-TRZ | 506 | 45 | 32.6/-/32.2 | *J. Am. Chem. Soc.* **2022**, *144*, 22976-22984. |
| **3DMAC-TB** | DPEPO | 508 | - | 38.8/-/30.2 | *Chem. Eng. J.* **2022**, *450*, 137805. |
| **BNB-m** | PPF | 508 | 74 | 34.9/-/27.4 | *Adv. Opt. Mater.* **2022**, *10*, 2201071. |
| **DCzBN–Au** | DMIC-TRZ | 510 | 34 | 35.8/-/35.7 | *Adv. Mater.* **2023**, *35*, 2208378. |
| **ω-DABNA** | DOBNA-Ph | 512 | 25 | 31.1/30.8/29.4 | *J. Am. Chem. Soc.* **2022**, *145*, 1505-1511. |
| **TCzBN-BP** | SF3TRZ | 512 | 35 | 35.6/33.2/16.0 | *Adv. Opt. Mater.* **2023**, *11*, 2202950. |
| **TRZCzPh-BNCz** | CBP | 513 | 37 | 32.5/30.5/22.9 | *Angew. Chem. Int. Ed.* **2022**, *134*, e202210210. |
| **TRZTPh-BNCz** | CBP | 513 | 33 | 31.4/29.5/23.1 | *Angew. Chem. Int. Ed.* **2022**, *134*, e202210210. |
| **2Cz-PTZ-BN** | PhCzBCz | 516 | 56 | 32.8/30.8/23.5 | *Angew. Chem. Int. Ed.* **2022**, **61**, e202116297. |
| **BN-XTO** | DMIC-TRZ | 516 | 34 | 37.3/34.1/18.6 | *Angew. Chem. Int. Ed.* **2022**, *62*, e202302478. |
| **BN-STO** | DMIC-TRZ | 517 | 34 | 40.1/39.0/28.1 | *Angew. Chem. Int. Ed.* **2022**, *62*, e202302478. |
| **DBTN-2** | SF3-TRZ | 520 | 29 | 35.2/33.6/20.4 | *Nat. Photon.* **2023**, *17*, 280-285. |
| **PFDMAC-TRZ** | DPEPO | 521 | - | 35.1/24.9/19.4 | *Adv. Opt. Mater.* **2022**, *10*, 2102441. |
| **CzBN1** | DMIC-TRZ | 523 | 75 | 31.7/-/30.5 | *J. Am. Chem. Soc.* **2023**, *145*, 12550-12560. |
| **DPFDMAC-TRZ** | DPEPO | 524 | - | 37.0/33.5/23.7 | *Adv. Opt. Mater.* **2022**, *10*, 2102441. |
| **2BOICz** | mCPBC | 528 | 91 | 40.4/40.3/35.9 | *Nat. Commun.* **2023**, *14*, 2394. |
| **DQBC** | mCPBC | 528 | 100 | 39.1/36.1/29.1 | *Adv. Mater.* **2021**, *33*, 2103293. |
| **m-Cz-BNCz** | PhCzBCz | 528 | 45 | 31.4/29.0/17.5 | *Angew. Chem. Int. Ed.* **2020**, *59*, 17442-17446. |
| **(P)-BN-Py** | PhCbBCz | 532 | 37 | 30.6/23.7/10.0 | *Adv. Mater.* **2023**, *35*, 2305125. |
| **1BOICz** | mCPBC | 534 | 101 | 34.6/33.8/31.7 | *Nat. Commun.* **2023**, *14*, 2394. |
| **TPAm2NPC** | mCPCN | 542 | - | 37.1/-/- | *ACS Appl. Mater. Interfaces* **2023**, *62*, e202306768. |
| **TPAmbPPC** | mCPCN | 545 | - | 37.9/-/- | *ACS Appl. Mater. Interfaces* **2023**, *62*, e202306768. |
| **3TPA-DiKTa** | mCP | 551 | 62 | 30.8/18.1/7.3 | *Angew. Chem. Int. Ed.* **2022**, *61*, e202213697. |
| **BNIP-tBuDPAC** | DMIC-TRZ | 554 | 58 | 39.4/37.1/23.3 | *Adv. Funct. Mater.* **2023**, *33*, 2213056. |
| **BNIP-tBuCz** | DMIC-TRZ | 566 | 69 | 32.8/29.6/15.5 | *Adv. Funct. Mater.* **2023**, *33*, 2213056. |
| **SpAcDBA** | CBP | 567 | 96 | 30.0/-/- | *ACS Appl. Mater. Interfaces* **2020**, *12*, 23199-23206. |
| **3,6,11-triTPA-BPQ** | CBP | 576 | - | 30.3/-/- | *J. Mater. Chem. C* **2023**, *11*, 6018-6025. |
| **BNDIP** | DMIC-TRZ | 582 | 67 | 34.1/33.5/25.5 | *Adv. Funct. Mater.* **2023**, *33*, 2213056. |
| **BNIP-CzDPA** | DMIC-TRZ | 584 | 62 | 32.4/27.1/15.2 | *Adv. Funct. Mater.* **2023**, *33*, 2213056. |
| **TCZ-F-DABNA** | PhCzBCz | 588 | - | 39.2/-/- | *Angew. Chem. Int. Ed.* **2022**, *134*, e202212575. |
| **DCN-PhTPA** | CBP | 596 | - | 33.8/15.1/7.1 | *Adv. Funct. Mater.* **2023**, *33*, 2304398. |
| **BPSPXZ** | CBP | 604 | - | 33.4/-/21.0 | *Small*. **2022**, *18*, 2201548. |
| **DCPPr-a-NDPA** | CBP | 606 | - | 31.5/-/- | *Angew. Chem. Int. Ed.* **2022**, *133*, 23827-23832. |
| **TPA-APQDCN-C** | CBP | 610 | - | 34.3/-/- | *Adv. Opt. Mater.* **2022**, *11*, 2102441. |
| **DCN-SP-DPA** | CBP | 612 | - | 36.9/16.0/- | *Adv. Opt. Mater.* **2023**, *11*, 2201191. |
| **DCN-SPTPA** | CBP | 656 | - | 36.1/13.2/4.7 | *Adv. Funct. Mater.* **2023**, *33*, 2304398. |
| **DABNA-3B** | SiTrzCz2 | 475 | 25 | 33.8/30.6/25.1 | **This work** |
| **BCzBN-3B** | DMIC-TRZ | 493 | 22 | 42.6/40.4/30.5 | **This work** |

# 3. References

1. V. Dolomanov, L. J. Bourhis, R. J. Gildea, J. A. Howard, H. Puschmann, *J. Appl. Cryst.*, **2009**, *42*, 339-341.
2. G. M. Sheldrick, *Acta Cryst.*, **2015**, *A71*, 3-8.
3. G. M. Sheldrick, *Acta Cryst.*, **2015**, *C71*, 3-8.
4. X. Gao, S. Bai, D. Fazzi, T. Niehaus, M. Barbatti, W. Thiel, *J. Chem. Theory Comput.*, **2017**, *13*, 515-524.
5. Q. Zhang, H. Kuwabara, W. J. Potscavage, S. Huang, Y. Hatae, T. Shibata, C. Adachi, *J. Am. Chem. Soc.* **2014**, *136*, 18070-18081.
6. Q. Zhang, B. Li, S. Huang, H. Nomura, H. Tanaka, C. Adachi, *Nat. Photonics* **2014**, *8*, 326-332.
7. T.-L. Wu, M.-J. Huang, C.-C. Lin, P.-Y. Huang, T.-Y. Chou, R.-W. Chen-Cheng, H.-W. Lin, R.-S. Liu, C.-H. Cheng, *Nat. Photonics* **2018**, *12*, 235-240.
8. S.-F. Wu, S.-H. Li, Y.-K. Wang, C.-C. Huang, Q. Sun, J.-J. Liang, L.-S. Liao, M.-K. Fung, *Adv. Funct. Mater.* **2017**, *27*, 1701314.
9. S. Reineke, K. Walzer, K. Leo, *Phys. Rev. B* **2007**, *75*, 125328.
10. Q. Zhang, S. Sun, W. Liu, P. Leng, X. Lv, Y. Wang, H. Chen, S. Ye, S. Zhuang, L. Wang, *J. Mater. Chem. C* **2019**, *7*, 9487-9495.
